# Pyridine-type alkaloid composition affects bacterial community composition of floral nectar

**DOI:** 10.1038/srep11536

**Published:** 2015-06-30

**Authors:** Yana Aizenberg-Gershtein, Ido Izhaki, Rakesh Santhanam, Pavan Kumar, Ian T. Baldwin, Malka Halpern

**Affiliations:** 1 Department of Evolutionary and Environmental Biology, Faculty of Natural Sciences, University of Haifa, Mount Carmel, Haifa, Israel; 2 Department of Molecular Ecology, Max Planck Institute for Chemical Ecology, Jena, Germany; 3 Department of Biology and Environment, Faculty of Natural Sciences, University of Haifa, Oranim, Tivon, Israel

## Abstract

Pyridine-type alkaloids are most common in *Nicotiana* species. To study the effect of alkaloid composition on bacterial community composition in floral nectar, we compared the nicotine-rich wild type (WT) *N. attenuata*, the nicotine biosynthesis-silenced *N. attenuata* that was rich in anatabine and the anabasine-rich WT *N. glauca* plants. We found that the composition of these secondary metabolites in the floral nectar drastically affected the bacterial community richness, diversity and composition. Significant differences were found between the bacterial community compositions in the nectar of the three plants with a much greater species richness and diversity in the nectar from the transgenic plant. The highest community composition similarity index was detected between the two wild type plants. The different microbiome composition and diversity, caused by the different pyridine-type alkaloid composition, could modify the nutritional content of the nectar and consequently, may contribute to the change in the nectar consumption and visitation. These may indirectly have an effect on plant fitness.

Most of the plant species on earth are animal-pollinated^1^ and the most important calorific and nutritional reward which plants offer to attract pollinators is the floral nectar^2^. The nutritional and energetic content of floral nectar plays an important role in attracting legitimate pollinators and in deterring nectar robbers^3^,^4^. The role of nectar’s bacterial communities in attracting pollinators to this “nutritional and energetic content” have been overlooked until recently, when a few studies demonstrated that bacterial communities in nectar are abundant and diverse^5^,^6^,^7^,^8^,^9^. This is in contrast to the assumption that nectar is not suitable as bacterial habitat and even has antimicrobial properties^10^.

Despite the dominance of sugar (>90% dry weight), non-sugar compounds such as secondary metabolites are also present in floral nectar^11^,^12^,^13^. Non-protein amino acids, phenolic compounds, and alkaloids are extremely common in floral nectar^14^,^15^,^16^. Secondary metabolites were found to be important in plant-bacteria interactions^17^; however, their impact on bacterial community composition in floral nectar is unknown. The presence of a secondary metabolite in the nectar may promote bacterial species that can utilize it or exclude bacteria that are inhibited by it^18^. Here we examined the effect of the composition of nicotine, anatabine and anabasine, three main alkaloids in the floral nectar of *Nicotiana* species on their bacterial community’s composition, richness and diversity. We hypothesized that pyridine-type alkaloids play a role in shaping the bacterial community in floral nectar.

Kessler and Baldwin^19^ raised an evolutionary dilemma about the presence of nicotine in nectar. Is the presence of nicotine in nectar an unavoidable consequence of a plant’s defense mechanism^20^, or does it have a different adaptive function in the nectar? Kessler and Baldwin^21^ demonstrated that individual metabolites (in experimental nectars presented in artificial flowers) directly affected the interaction between the plant and its floral visitors^21^ and thus may affect pollination efficiency. Another hypothesis for why plants produce secondary metabolites in nectar relates to the possible role that these metabolites play in shaping the bacterial community composition in nectar, which, in turn, may have an indirect effect on the attraction or deterrence of plant visitors.

Recently, we showed that honeybees (*Apis mellifera*) and other insects [*Capsodes infuscatus* (Hemiptera: Miridae)] are factors in shaping bacterial community composition in floral nectar^7^,^9^. Belisle *et al*.^22^ demonstrated that microfungi are transported to flowers by hummingbirds. The presence of a single secondary metabolite in the nectar modifies the assemblage of nectar pollinators and consumers that may act as vectors of microorganisms^21^,^23^,^24^ and, thus, is likely to affect the transfer of bacteria into the nectar. For example, nicotine and caffeine elicited a significant feeding preference of bees in *Nicotiana* spp. and *Citrus* plants^12^, respectively, whereas sunbirds were found to be deterred by nicotine and anabasine in *Nicotiana glauca* nectar^24^.

*Nicotiana attenuata* is a diploid tobacco that is native to the Great Basin Desert, Utah, USA. This plant species was developed as a model system for studying the genetic basis of important traits for ecological interactions^25^. Nicotine is the most abundant alkaloid of *N. attenuata*, with an average concentration of 27.6 ppm in nectar^26^. It is synthesized in the roots and is transported throughout the plant, where it is known to be an efficient defense against herbivores^20^. Steppuhn *et al*.^27^ transformed native *N. attenuata* to down-regulate nicotine expression, by silencing putrescine-N-methyl transferase (to produce irPMT plants), and demonstrated that nicotine protects the plant from herbivores. They also found that anatabine (another pyridine-alkaloid), was undetectable in the WT plants, while in the irPMT plants its concentrations increased. No other differences were found between the transformed and the WT plants, either in nectar secondary metabolites, floral scent, nectar volume, sugar concentrations, or growth parameters.

*Nicotiana glauca* is a South American native plant which has invaded semi-arid parts of the world^27^. The nectar of this species contains a relatively high concentration in of anabasine (5.0 ± 0.8 ppm^24^) and much lower concentration of nicotine (0.5 ppm). Nicotine concentration in *N. glauca* nectar is about 55 times lower compared to its concentration in the nectar of *N. attenuata* plants^24^,^26^. The important alkaloid in the nectar of irPMT plants which lacks nicotine - is anatabine (41.35 ± 1.75 ppm, n = 10 plants, Ian T. Baldwin, unpublished data). Thus, the nectar of each of these three plants has a different pyridine-alkaloid composition.

The aim of the current study was to examine whether the pyridine-alkaloid composition in the floral nectar affects the bacterial community composition in nectar. To achieve this goal, we first compared the bacterial community composition in the nectar of irPMT *N. attenuata* plants (with silenced nicotine expression) with that in the nectar of *N. attenuata* WT plants. In addition, we compared the bacterial community composition in the nectar of a different *Nicotiana* species (*N. glauca*) with that of both *N. attenuata* WT and irPMT plants. We used *N. glauca* as an outlier, given that, among the *Nicotiana* species, its nectar is characterized by relatively low nicotine and high anabasine concentrations. We were able to demonstrate that the elimination of a single metabolite - nicotine, which resulted in anatabine accumulation, altered the bacterial communities in the floral nectar of *N. attenuata* and also that the bacterial community in the outliner species (*N. glauca*) was more similar to that of *N. attenuata* WT than to the irPMT plants.

## Methods

### Study system - nectar chemical composition

irPMT *N. attenuata* plants are unable to synthesize nicotine, due to the silencing of the key nicotine biosynthetic enzyme, putrescine-N-methyl transferase, in an inverted repeat orientation. Full characterization of the irPMT plants can be found in Steppuhn *et al*.^27^. irPMT plants have drastically reduced transcripts of both PMT genes, and they do not produce any detectable quantities of nicotine. All compounds found in the floral scent of WT plants, with the expected exception of nicotine (t-test, t_14_= −4.86, P < 0.001), can be detected in statistically equivalent amounts in the floral scent of the transformed plants. Sugar concentrations in nectar did not differ among WT and irPMT plants (t-test, t_24_ = 0.32, P = 0.75)^21^. As for nectar metabolites, irPMT plants accumulate the alkaloid anatabine up to the concentration of 41.35 ± 1.75 ppm (Ian T. Baldwin, unpublished data). Anatabine is not detectable in the WT plants. Elevated anatabine levels did not affect transcript levels of other gene-encoding enzymes involved in alkaloid metabolism^28^. irPMT plants did not differ from WT plants in any other measured secondary metabolite or growth parameter.

### *Nicotiana* plants and nectar collection

No specific permissions were required for plant samplings, since the field studies did not involve endangered or protected species. Floral nectar was collected from two *Nicotiana* species: *N. attenuata* and *N. glauca*. The *N. attenuata* WT and irPMT plants were sampled at the glasshouse of Max Planck Institute for Chemical Ecology, Jena, Germany, in October - November 2011 and January - February 2014, for culture-independent and culture-dependent techniques, respectively. *N. glauca* was sampled in open areas near Be’er Sheba, Israel, in November 2013. *N. attenuata* seeds (irPMT and WT plants) were germinated as previously described by Kruegel *et al*.^29^ and grown in individual pots, side by side in the same environmental conditions in a glasshouse. These plants were not exposed to any floral visitors. Pollinator visitation is known to differ between the WT and irPMT^22^ and because pollinators can act as vectors, this would have been a confounding variable. Using greenhouse-grown plants allowed a clearer test of the effects of nicotine alone.

All plants were sampled between 07:00 am–08:00 am. For each plant sample, nectar was collected from 20–30 flowers (at least 100 μl), using sterile tips (under sterile conditions to avoid contamination).

For the culture-dependent approach, the nectar was collected from five *N. attenuata* (WT) and five irPMT plants. For the culture-independent approach, nectar was collected from six *N. attenuata* WT, five *N. attenuata* irPMT, and five *N. glauca* WT (ISWT) plants.

### Culture-dependent approach

One nectar sample from each plant got split onto four different isolation media: (i) tap water-yeast extract agar [TWYE; 0.25 g of yeast extract (Roth), 0.5 g of K_2_HPO_4_, and 16 g of agar (Roth) per L of tap water]; (ii) *Streptomycetes* isolation media (SIM; 0.4 g casein, 1.0 g starch, 0.5 g KNO3, 0.2 g K_2_HPO4, 0.1 g MgSO4, 0.1 g CaCO_3_ and 16 g agar in 1L distilled water); (iii) R2A agar (Fluka Analytical, Germany) and (iv) Nutrient agar (NA, Fluka Analytical, Germany). No colonies were observed in SIM and TWYE media. After incubation, colonies were picked from R2A and NA plates, sub-cultured and purified. For long-term use, each culture was suspended in 50% glycerol solution and stored at −80 °C.

#### Species identification procedure

A total of 83 16S rRNA amplifications were performed for the bacterial isolates, using Ready Mix Taq PCR reaction mix (Sigma-Aldrich), template DNA and 50 μM primers [27F (5'-AGAGTTTGATCCTGGCTCAG-3') and 1492R (5'-GGTTACCTTGTTACGACTT-3')]^29^. The reaction conditions were as follows: 95 °C for 1 min, followed by 30 cycles of denaturation at 95 °C for 30 s, annealing at 53 °C for 30 s and primer extension at 72 °C for 30 s, followed by a final extension at 72 °C for 5 min. Direct sequencing using the primer 783R (5'-CTACCAGGGTAT C TAATCCTG-3') 30 was conducted in Big Dye Mix (Applied Biosystems, Foster City, CA, USA), and purification of the sequencing reactions was performed using the Nucleo-SEQ Kit (Macherey-Nagel, Düren, Germany). Analysis of all sequences was carried out in EzTaxon server (http://eztaxon-e.ezbiocloud.net/)^31^. Based on the genera level, a phylogenetic tree was generated for all the 16S rRNA sequences, using neighbor-joining algorithms drawn from the MEGA 5 package^32^. An evolutionary distance matrix for the neighbor-joining analysis was prepared using the Jukes and Cantor model^33^. The robustness of the inferred tree topologies was evaluated after 1000 bootstrap replicates of the neighbor-joining data using MEGA 5 software. Nucleotide sequence accession numbers of the partial 16S rRNA sequences determined in this study were deposited in the GenBank (LK020716- LK020798).

#### Statistical analyses of the cultured communities

To determine whether the cultured bacterial communities in WT and irPMT nectar were significantly different, we used phylogenetic indices such as UniFrac^34^ and P-test significance test^35^.

### Culture-independent approach

DNA was extracted from the nectar samples using a DNA isolation kit (DNeasy Blood and Tissue, Qiagene, Germany) according to the manufacturer’s instructions.

#### Generation of the 16S rRNA gene library

Genomic DNA was PCR-amplified using primers targeting the V4 variable region of the bacterial small subunit (SSU) ribosomal RNA (rRNA) gene. The primers contained 5’ common sequence tags, as described previously (e.g., Moonsamy *et al*. 2013)^36^. The primers CS1_515F (ACACTGACGACATGGTTCTACAGTGCCAGCMGCCGCGGTAA) and CS2_806R (TACGGTAGCAGAGACTTGGTCTGGACTACHVGGGTWTCTAAT) (e.g. Caporaso *et al*. 2012)^37^ were synthesized by Sigma-Aldrich (Israel) as standard oligonucleotides.

PCR amplifications were performed in 25 μl reactions in PCR tubes. A mastermix was made using the EmeraldAmp MAX HS PCR Master Mix (Takara bio Inc). Final concentration of CS1_515F and CS2_806R primers was 0.5 ng/μl. Genomic DNA in the amount of 10–100 ng was added to each PCR reaction. Cycling conditions were as follows: 95 °C for 5 minutes, followed by 28 cycles at 95 °C for 30”, 55 °C for 45” and 68 °C for 30”. A final 7 minute elongation step was performed at 68 °C. Each sample was analyzed using agarose gel electrophoresis. Reactions were verified to contain visible amplification, in addition to no visible amplification in the no-template control, prior to the second stage of PCR amplification.

#### Illumina MiSeq sequencing

*MiSeq sequencing* was performed at DNA Services (DNAS) Facility – University of Illinois at Chicago (UIC). Before sequencing, a second PCR amplification was performed in a 10μl reaction in 96-well plates. A mastermix for the entire plate was made using the 2X AccuPrime SuperMix II. Each well received a separate primer pair, obtained from the Access Array Barcode Library for Illumina Sequences. The final concentration of each primer was 400 nM, and each well received a separate primer set with a unique 10-base barcode (Fluidigm, South San Francisco, CA; Item# 100-4876). Separate reactions with unique barcodes were included for positive control, no-template control (reaction 1), and a second no-template control reaction containing only Access Array Barcode library primers. Cycling conditions were as follows: 95 °C for 5 minutes, followed by 28 cycles at 95 °C for 30”, 60 °C for 30” and 68 °C for 30”. A final, 7 minute elongation step was performed at 68 °C. PCR yields of positive and negative controls and select samples were validated using Qubit fluorometric quantitation with the Qubit 2.0 fluorometer (Life Technologies) and with size and quantification employing an Agilent TapeStation2200 device with D1000 ScreenTape (Agilent Technologies, Santa Clara, California). After assessing no amplification in the negative controls, samples were pooled in equal volume and purified using solid phase reversible immobilization (SPRI) cleanup, implemented with AMPure XP beads at a ratio of 0.6X (v:v) SPRI solution to sample. This ratio removes DNA fragments shorter than 300 bp from the pooled libraries. The final quality control was performed using TapeStation2200 and Qubit analysis, prior to dilution to 6 pM for emulsion PCR.

Pooled, diluted libraries were sequenced on an Illumina MiSeq instrument, using a MiSeq 600-cycle sequencing kit version 3, and analyzed with Casava1.8 (pipeline 1.8). Reads were 200 nucleotides in length (paired end, 2 × 200). PhiX DNA was used as a spike-in control. Barcode sequences from Fluidigm were provided to the MiSeq server, and sequences were automatically binned according to 10-base multiplex identifier (MID) sequences. Raw reads were recovered as FASTQ files.

#### Sequence analysis

Bioinformatics were performed using MOTHUR v.1.33.3^38^. The operational taxonomic unit (OTU)-based approach of the MiSeq Standard Operating Procedure (SOP) was followed (Kozich *et al*. 2013^39^). Briefly, any sequences with ambiguities or homopolymers longer than 8 bases were removed from the data set. Sequences were aligned using the SILVA-compatible alignment database available within MOTHUR. Sequences were trimmed to a uniform length of 295 base pairs, and chimeric sequences were removed using Uchime (Edgar *et al*. 2011^40^). We classified sequences using the MOTHUR-formatted version of the RDP training set (v.9), and any unknown, chloroplast, mitochondrial, archaeal, or eukaryotic sequences were removed. Sequences were clustered into OTUs, based on 97% sequence identity. To avoid biases associated with uneven numbers of sequences across samples, the entire dataset was randomly subsampled to the minimum number of sequences per sample (lowest number of sequences that were obtained in a sample): 2146 sequences per sample.

All of the sequence data analyses reported in this paper can be downloaded from the National Center for Biotechnology Information (NCBI) Sequence Read Archive (SRA) with accession number SRP049451.

#### Microbial richness and similarity estimations

##### *Alpha Diversity*.

We calculated microbial richness in the floral nectar of each of the three plants (WT, irPMT, ISWT) on the subsampled OTUs and genera tables. The expected taxon (either OTU or genera) richness for the complete collection of each of the three plants was calculated by the abundance-based Chao 1. The Chao 1, a nonparametric, abundance-based richness estimator that predicts the true number of taxa based on the proportion of rare taxa in a sample^41^, was found to be suitable for microbial diversity analysis^42^. To remove the effect of sample order, the sample order was randomized 100 times and the mean richness estimate was computed for each sample accumulation level.

##### *Beta-Diversity*.

To evaluate the similarities of the bacterial community composition between each pair of the three plant types, we calculated the observed shared subsampled OTUs (Sobs) and the expected number of shared OTUs, using Chao’s coverage-based estimator^43^. In addition, we calculated Chao’s Estimated Jaccard and Sørensen incidence-based similarity indices, based on the OTU analysis. The two latter indices are incidence-based estimates of the probability that, if one individual is chosen from each of two plant types, both belong to OTUs that are shared between the plants^44^. This model also accounts for the probability that some OTUs were not sampled when actually present. This method allows for a bootstrapping approach (n = 200) to generate SD for the estimated similarities. All above calculations were performed using Estimate S9.1.0

To visualize beta-diversity among the three plant types (WT, irPMT, ISWT), we processed the data using the MOTHUR program (version 1.33.3; Schloss *et al*^38^), to generate the following: (a) A Venn diagram was generated to show the number of unique and overlapping taxon memberships between the three plant types at a distance of 0.03. (b) A dendrogram describing the similarity of the samples to each other was generated using the jclass calculator within the *tree.shared* command, which returns the traditional Jaccard index measuring the dissimilarity between two communities. Parsimony analysis and the UniFrac weighted algorithm were used to determine whether the clustering within the resulting dendrogram was statistically significant. (c) Ordination plots based on the jclass distance matrix between samples were plotted using the *dist.shared* command with Principal Coordinates Analysis (PCoA) in 2 dimensions. Two methods were used to statistically analyze the plots: (1) analysis of molecular variance (AMOVA), which tests whether the centers of the clouds of samples representing each plant type are more separated than the variation among the samples of the same plant species, and (2) Bartlett’s test for homogeneity of variance, which determines whether the variation in each of the plant type samples is different.

## Results

### Culture-dependent approach

Isolates belonging to a total of 33 different species and three classes (Table 1, total of 83 isolates; 43-WT, 40-irPMT) were identified in all of the nectar samples (WT and irPMT). The isolates were recovered only from two bacterial media R2A (23-WT, 20-irPMT) and NA (19-WT, 21-irPMT). Only five species (17 isolates) were found in both the WT and the irPMT nectar samples. Sixteen species (35 isolates) were recovered only from the WT nectar, and 12 species (31 isolates) were recovered only from the irPMT nectar. Five novel bacterial species (with 97–98% similarities in the 16S rRNA gene sequences to known species) were isolated (Table 1).

Bacterial communities in the WT nectar were evenly dominated by three different bacterial classes (*Actinobacteria, Bacilli* and *Alphaproteobacteria*, 32–35%). In contrast, the irPMT nectar was highly dominated by *Actinobacteria* (70%), followed by *Alphaproteobacteria* (20%), and *Bacilli* (10%). At the genus level, the relative position and diversity found among nectar bacterial communities of the WT and the irPMT plants was also reflected in the 16S rRNA phylogenetic tree (Fig. S1).

A phylogenetic tree was constructed based on species level and subjected to UniFrac and P-test significance test (http://unifrac.colorado.edu/). The bacterial communities from the WT and the irPMT nectar samples were significantly different [UniFrac (*P* = 0.01) and P-test (*P* = 0.03)], demonstrating that nicotine has a direct effect on nectar’s bacterial community composition.

### Culture-independent approach

#### General characteristics of nectar communities

Bacterial communities of nectar from six *N. attenuata* wild type (WT), five *N*. attenuata irPMT (irPMT), and five *N.glauca* wild type (ISWT) plants were studied using illumine MiSeq technique. Across all nectar samples (16 plants in total), we obtained 91,445 quality sequences (after removal of 691,006 sequences that were identified as chloroplast, mitochondrial, archaeal, or eukaryotic sequences). Those sequences were classified in a total of 1101 unique OTUs at the 97% sequence similarity level across all samples. After subsampling all the 16 samples to the smallest sample (2146 sequences), the total of 34,336 sequences were classified into 578 OTUs (97% sequence similarity). OTU data with taxonomic classifications and abundance within each plant type are shown in Table S1. The abundance-based coverage value at the 3% criteria for irPMT, WT, and ISWT plants were 96.2%, 99.3%, and 99.5% respectively. At 3% sequence divergence, most rarefaction curves, describing the number of OTUs observed as a function of sampling effort, asymptote [full data (Fig. S2a) and subsampled data (Fig. S2b)]. These indicate that the surveying effort covered almost the full extent of taxonomic diversity at this genetic distance (Fig. S2).

#### Composition and richness of bacterial communities in the nectar

**Figure 1:**
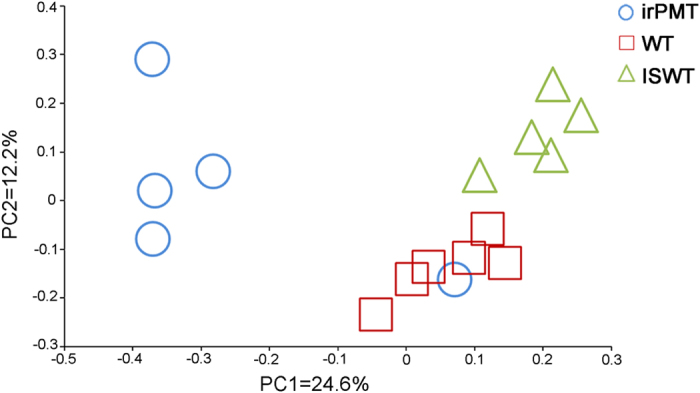
Principal coordinates analysis (PCoA) based on OTU relative abundances of the bacterial community composition in the floral nectar of *N*. *attenuata* WT, transgenic *N. attenuata* irPMT and *N*. *glauca* ISWT samples. The first two principal coordinates (PC1 and PC2) from the principal coordinate analysis of unweighted UniFrac are plotted for each sample. The variance explained by the PCs is indicated on the axes.

Bacterial isolates found in *N. attenuata* WT nectar were predominantly of the class *Bacilli* (38.5%), followed by *Actinobacteria* (22.8%), and *Gammaproteobacteria* (20.3%). In the irPMT nectar samples, bacterial isolates were predominantly of the *Bacilli* class (36.4%), followed by *Actinobacteria* (20.5%), and *Gammaproteobacteria* (11.8%). Sequence analysis showed significant differences between the bacterial communities from the nectar of the wild type (*N. atttenuata*) and the transgenic *N. attenuata* (AMOVA, *F*_1,9_ = 2.34; *P* = 0.01), as was also demonstrated by the PCoA ordination that was based on OTU relative abundances (Fig. 1).

We further compared the bacterial communities from nectar of *N. attenuata* WT and transgenic plants with nectar that was obtained from another *Nicotiana* plant species (*N. glauca* - ISWT) as an outlier. The majority of the bacterial sequences from nectar of ISWT plants were classified as *Gammaproteobacteria* (61.8%), followed by *Bacilli* (14.9%) and *Actinobacteria* (9.8%). The nectar of the three plant types differed in their bacterial taxonomic richness, both at the OTU and at the genus levels. The nectar of the transgenic *N. attenuata* (irPMT) had 4–5 times and 7–9 times higher observed and expected bacterial OTU richness compared to the *N. attenuata* WT and *N. glauca* nectar, respectively (Sobs Mean and Chao1 estimators, Table 2). ISWT had the lowest number of taxa, whereas the *N. attenuata* WT was intermediate. Thus, the transgenic *N. attenuata* (irPMT) nectar harbored a much richer bacterial community than did the two wild-type plants.

The two incidence-based similarity indices, Chao-Sørensen and Chao-Jaccard, which were used to compare bacterial community composition among the three plant types, demonstrated that the bacterial community composition in the nectar of the irPMT plants was about as different from the composition in the WT nectar as it was from that in the ISWT nectar (Table 3). The highest similarity values were found between the nectar samples of the two wild type plants (WT and ISWT, Table 3).

The principal coordinates analysis (PCoA) of the sequenced data showed a clear clustering of the various samples from each plant type in a distinct region of the ordination (with the exception of one sample from the irPMT plant that was clustered with the WT samples, Fig. 1). The AMOVA that was used to assess the statistical significance of the spatial separation observed among the three plant types in the PCoA ordination plots showed statistically significant dissimilarities across the three groups (*F*_2,6_ = 2.98, *P* < 0.001). Bartlett’s test for homogeneity of variances demonstrated that there was a significant difference in the variation of the samples obtained from the three plant types (*P* = 0.005).

**Figure 2:**
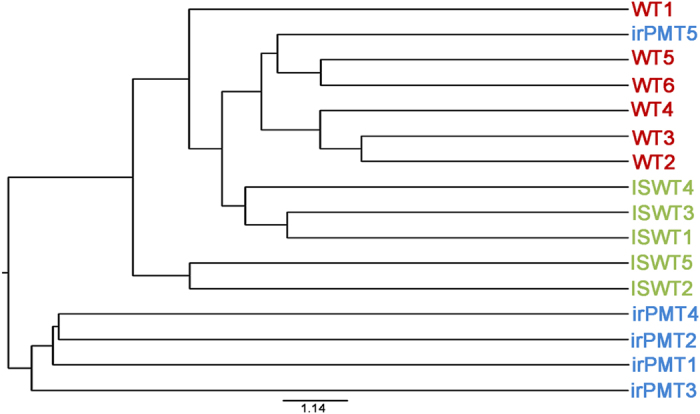
UniFrac dendrogram showing the similarity of the floral nectar samples obtained from each plant type (WT- *N. attenuata* wild type, irPMT - transgenic *N. attenuata*, and ISWT *N. glauca*), using Jaccard coefficient (jclass) structural diversity measure.

The dendrogram of the community cluster analysis under the Jaccard coefficient (jclass) structural diversity measure showed separated positions of the bacterial community in the floral nectar of each of the three plant types (Fig. 2). *N. attenuata* WT samples were closer to *N. glauca* ISWT samples than to the samples obtained from *N. attenuata* irPMT (Fig. 2). Parsimony analysis of this UniFrac dendrogram showed a significant difference for the data corresponding to the clustering of the samples between *N. glauca* ISWT and *N. attenuata* WT (*P* < 0.05), between irPMT and WT (*P* < 0.05), and marginally significant between irPMT and ISWT plants (*P* = 0.08). UniFrac weighted analysis, which determines whether two or more communities have statistically significant structural dissimilarities, indicated statistically significant dissimilarities among the samples of each pair from the three studied plant types (*P* < 0.001).

**Figure 3:**
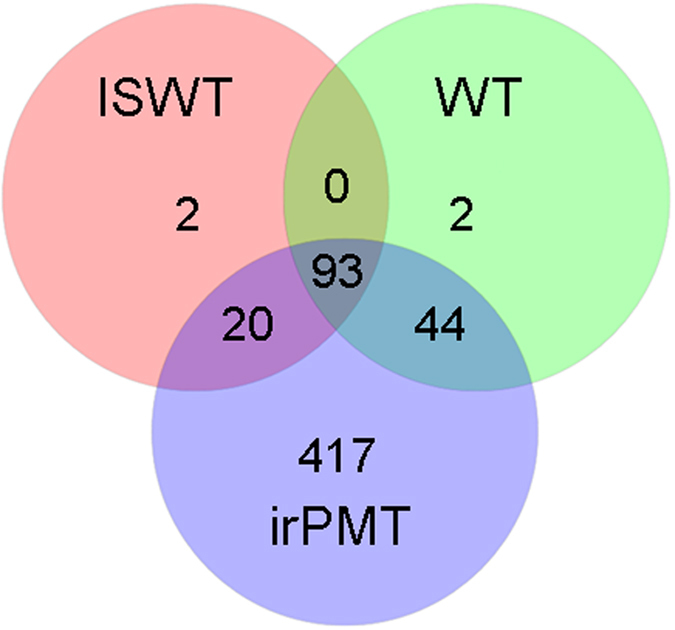
Venn Diagram at distance 0.03, illustrating the number of unique and shared subsampled OTUs (97% sequence similarity), within the libraries of the bacterial communities in the nectar of the three studied plant types, *N. attenuata* WT, *N. attenuata* irPMT, and *N. glauca* ISWT.

The Venn diagram (Fig. 3) showed that *N. attenuata* irPMT had the highest number of unique OTUs (417/578 = 72.1%). The two wild types had only two (0.3%) unique OTUs. It was also demonstrated that the irPMT plants shared a low fraction of OTUs with the WT plants (7.6%) and with the ISWT plants (3.4%). Only 93 (16.1%) OTUs overlapped among the three plant types (Fig. 3).

Species belonging to the genera *Gardnerella* and *Lactobacillus* were detected only in irPMT plants, *Enterococcus* was found only in the WT plants, and *Enhydrobacter* and *Vibrio* were detected only in the ISWT plants (Table S2). Full taxonomic classifications in the genera level are shown in Table S2.

## Discussion

Here we have demonstrated that the elimination of a single secondary metabolite - nicotine, which caused an elevation of another secondary metabolite - anatabine, drastically affected the composition and diversity of bacterial communities in floral nectar. This was shown both by culturable and as well as by unculturable methods. The culture-dependent method demonstrated that only five out of 33 different bacterial species were isolated from both *N. attenuata* WT and the transgenic irPMT plants (Table 1). By using the MiSeq illumina technique, we found that only 23.7% of the total observed OTUs (137/578) were identified in both the *N. attenuata* WT and the transgenic irPMT plant. The bacterial community composition in the nectar of the outlier plant species within the genus (*N. glauca*) was different from both *N. attenuata* WT and the transgenic irPMT plants. However, the similarity indices between the two wild type plants were much higher than between each one of them and the irPMT plants.

A possible explanation for this observation is a differential response of the bacterial community to the different pyridine-type composition of the nectar of these three plants. Studies showed that nicotine interferes with the growth of microorganisms^45^. Nicotine is undetectable in irPMT plants while both wild type plants contain nicotine, although about 55 times lower in *N. glauca* compared to its concentration in *N. attenuata* WT plants. Yet, the effect of nicotine on the bacterial community might be crucial even in relatively low concentrations and thus leads to a relatively similar community composition of the two wild type plants. Furthermore, anabasine which is the most dominant alkaloid in *N. glauca* is known to possess pronounced antibacterial properties^46^,^47^. Thus, anabasine may have a similar prominent impact on the bacterial community as nicotine.

Furthermore, the taxonomic richness of the bacterial communities in the floral nectar of the irPMT plants was 574 and 1134 for Sobs and Chao1 expected OTUs, respectively (Table 2). These values were much higher than in the two wild type plants. Such high richness and diversity are probably due to the elimination of nicotine, which is considered an extremely toxic alkaloid^25^,^48^, from the irPMT plants and/or due to the presence of high anatabine concentrations. This suggests that anatabine, which is characterized only in *N. attenuata* irPMT plants, enabled the inhabitation of many more bacteria than nicotine and anabasine. Indeed, Steppuhn *et al*. (2004)^27^ demonstrated strong evidence that nicotine but not anatabine efficiently protects plants against herbivorous insects. There are no parallel studies that compare the antibacterial effects of nicotine and anatabin. However, we suggest that while the presence of nicotine and anabasine inhibited some bacterial species proliferation in the nectar, the presence of anatabine supported it.

Studies showed that some bacteria have developed nicotine resistance^18^. For example, a study on bacterial communities inhabiting *Nicotiana* leaves demonstrated that some bacterial species were adapted to nicotine as a growth substrate, and developed biochemical strategies to decompose this organic heterocyclic compound (reviewed by Brandsch^49^). In the current study, we observed a relatively low bacterial OTU numbers in the nectar of the WT and the ISWT plants. Some of these OTUs are probably adapted to nicotine as a growth substrate. However, as far as we know, the influence of anatabine on bacterial growth was never studied before.

The nectar from *N. attenuata* WT and from the transgenic irPMT plants that were grown in a closed greenhouse had significantly different bacterial community composition. Secondary metabolism is known to be sensitive to environmental parameters that differ between glasshouse and field conditions (e.g., UV-B influence^50^). However, in a previous study^27^, no significant difference was found between plants that were grown in the field and in the glasshouse in nicotine and anatabine levels (either in the WT or in the irPMT plants). Recently, we showed that nectar consumers may act in a natural environment as bacterial vectors^7^,^9^ and affect the transfer of bacteria into the nectar. Although WT and irPMT plants in the current study were not exposed to visitors, as they both were grown in a greenhouse with no access to nectar consumers or pests, they nevertheless possessed diverse bacterial communities. This indicates that bacteria are probably transferred to the nectar also by other means, such as soil, irrigation, pesticides, and air and not only through insects or other nectar consumers.

Nectar of *N. attenuata* with all its constituents has the potential to filter flower visitors, favoring some and deterring others. Here we demonstrated how the pyridine-alkaloids (nicotine, anatabine and anabasine) influence the bacterial community composition in the floral nectar of *N. attenuata*. The differences in the nectar bacterial communities between the three plants may also have an effect on nectar consumption by floral visitors. This is because different bacterial species may degrade different nutritional substrates into different products; thus, these secondary metabolites, through their effect on the bacterial community composition, may have also an indirect effect on the nectar’s nutritional composition and consequently, on the floral visitors and pollination.

Little is known about how specific microbial communities are biostimulated by an individual plant’s secondary metabolites. Uhlik *et al*.^51^ showed that plant secondary metabolites have a strong effect on the bacterial communities’ activity and degradative ability. Yuan *et al*.^48^ demonstrated that nectar odors directly impact plant reproductive fitness through pollinator attraction or deterrence of nectar robbers and florivores. Kessler and Baldwin^21^ tried to understand the purpose of nicotine presence in nectar. The results of the current study indicate that secondary metabolites play a role in shaping the composition of the bacterial communities in nectar. These may indirectly have an effect on attracting or deterring plant visitors and thus, may explain the evolutionary role of nicotine (or other secondary metabolites) in the nectar. Furthermore, bacterial nectar could provide direct benefit to insect pollinators, for example, by assisting in the insects’ digestion, especially because insects lose their gut microbes each time they molt^52^.

## Conclusion

The aim of this study was to examine whether the bacterial community composition in nectar varied with an artificially-induced change in a single metabolite (nicotine). This change led to a different pyridine-alkaloid composition among the studied *Nicotiana* species. By using both a culture-dependent approach and MiSeq illumine sequencing, we showed that bacterial community composition in floral nectar varies between different *Nicotiana* plant types and especially between *N. attenuata* WT and the genetically transformed irPMT plants, which are unable to produce nicotine. Yeast metabolism has been shown to significantly contribute to floral scent in other plant genera^53^; therefore, bacteria in nectar might contribute to variation in plant signaling and eventually, even to pollination success and plant fitness. This study is the first step toward examining our hypothesis that different bacterial communities in nectar that are formed by a change in a single metabolite (which increased another metabolite) and by the pyridine-alkaloid composition, may affect nectar consumption, plant pollination and plant fitness.

## Additional Information

**How to cite this article**: Aizenberg-Gershtein, Y. *et al*. Pyridine-type alkaloid composition affects bacterial community composition of floral nectar. *Sci. Rep*. **5**, 11536; doi: 10.1038/srep11536 (2015).

## Supplementary Material

Supplementary Information

## Figures and Tables

**Table 1:**
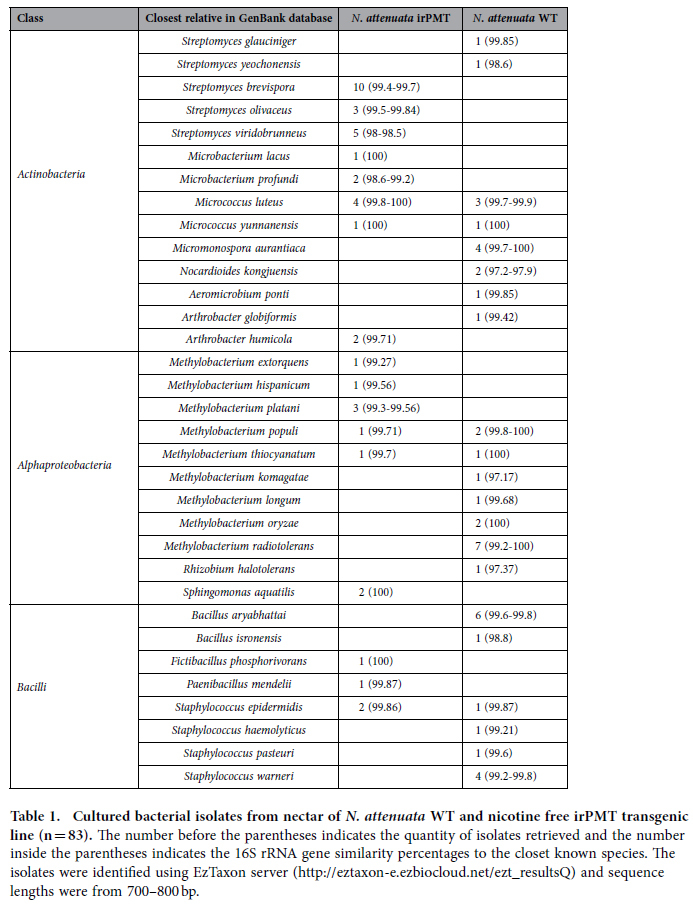
Cultured bacterial isolates from nectar of *N. attenuata* WT and nicotine free irPMT transgenic line (n = 83).

**Table 2:** Microbial richness of the three plants’ nectar of the subsampled OTU’s and genera levels.

Phylogenetic level		*N. attenuata* WT	*N. attenuata* irPMT	*N. glauca* ISWT
OTU’s	Sobs Mean	139	574	115
	Chao1	160	1134	122
	(95% CI)	(147–196)	(976–1355)	(117–139)
Genera	Sobs Mean	104	315	89
	Chao1	111	372	90
	(95% CI)	(106–130)	(343–428)	(89–96)

Sobs Mean is the average number of different taxonomic units in the samples and Chao1 is an estimator of the expected taxonomic richness for the complete collection of each of the three plants (see details in the Methods).

**Table 3:** Pairwise similarity indices of bacterial community composition in the floral nectar of the three plant types, based on subsample of the OTU table.

	*N. attenuata* WT - *N. glauca* ISWT	*N. attenuata* WT - *N.attenuata* irPMT	*N.attenuata* irPMT - *N. glauca* ISWT
Shared OTU’s	93	137	113
Chao OTU Shared Estimated	103	150	133
Chao-Sørensen-Est similarity index	0.883	0.694	0.649
Chao-Sørensen SD	0.033	0.012	0.017
Chao-Jaccard similarity index	0.791	0.532	0.480
Jaccard SD	0.054	0.015	0.019
