# Supplementary material for: Pyridine-type alkaloid composition affects bacterial community composition of floral nectar
Source: Sci Rep. 2015 Jun 30;5:11536. doi: 10.1038/srep11536 (PMC4650603; doi:10.1038/srep11536)
Supplement: Supplementary Information [file srep11536-s1.pdf]

Pyridine-type alkaloid composition affects bacterial community composition of floral nectar

Yana Aizenberg-Gershtein, Ido Izhaki,Rakesh Santhanam, Pavan Kumar, Ian T. Baldwinand Malka Halpern

Supplementary data

Fig. S1. Neighbor-joining tree of bacterial communities isolated from nectar of *N. attenuata*WT **(A)** and nicotine free irPMT **(B)** plants. The tree was generated based on partial 16S rRNA gene sequences (~700 bp). Numbers at the nodes are percentage bootstrap values based on 1,000 resampled datasets. Bar - 0.05 substitutions per nucleotide position.

Fig. S2. Rarefaction curves indicating the observed number of operational taxonomic units (OTUs) at a genetic distance of 3% in different plant types. **(A)** Including all data sequencing **(B)** including subsample data only.

Table S1. Taxonomic classifications and abundances of all the OTUs within each plant type.

Table S2. Full taxonomic classifications in the genera level within each plant type.

Figure S1

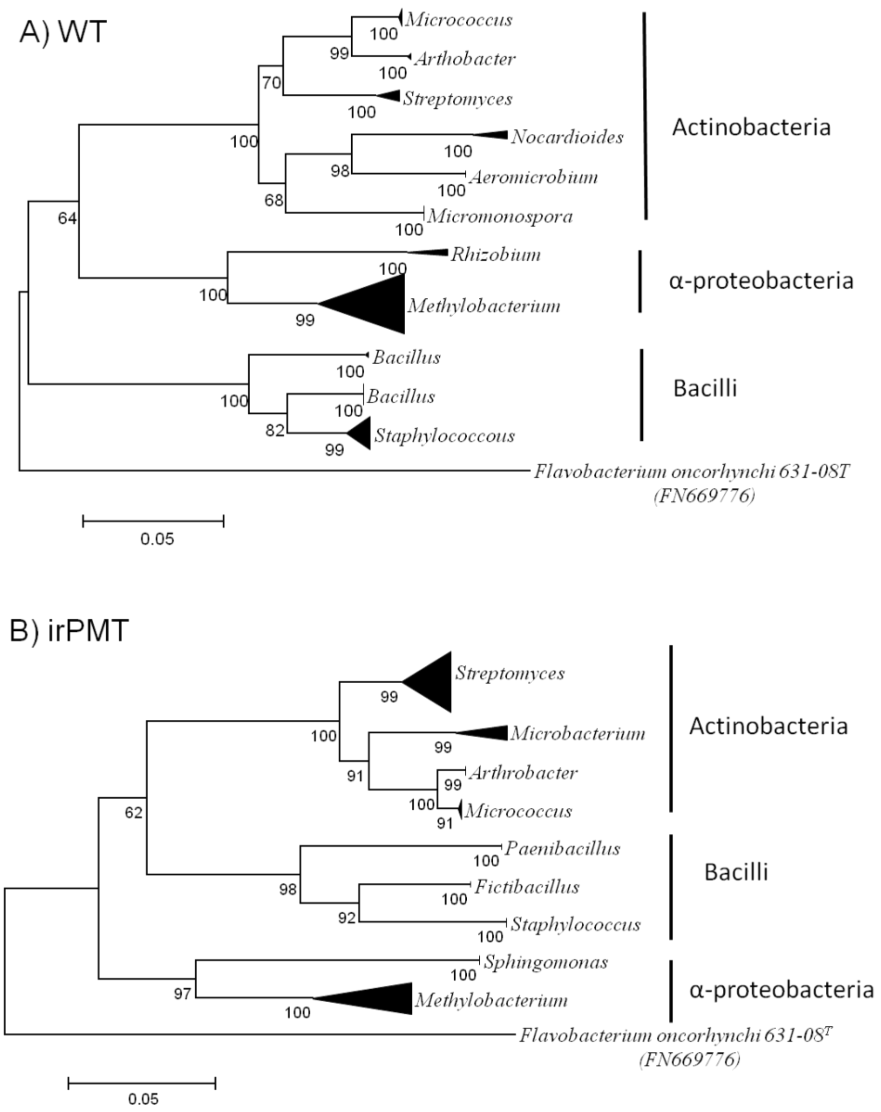
A) WT
Micrococcus
100
99
Arthobacter
100
70
Streptomyces
100
100
Nocardioides
100
98
Aeromicrobium
100
68
Micromonospora
100
64
Rhizobium
100
100
Methylobacterium
99
Bacillus
100
100
Bacillus
100
82
Staphylococcus
99
Flavobacterium oncorhynchi 631-08T
(FN669776)
0.05
Actinobacteria
α-proteobacteria
Bacilli
B) irPMT
Streptomyces
99
100
Microbacterium
99
91
Arthrobacter
99
100
Micrococcus
91
62
Paenibacillus
100
98
Fictibacillus
100
92
Staphylococcus
100
Sphingomonas
100
97
Methylobacterium
100
Flavobacterium oncorhynchi 631-08T
(FN669776)
0.05
Actinobacteria
Bacilli
α-proteobacteria

Figure S2

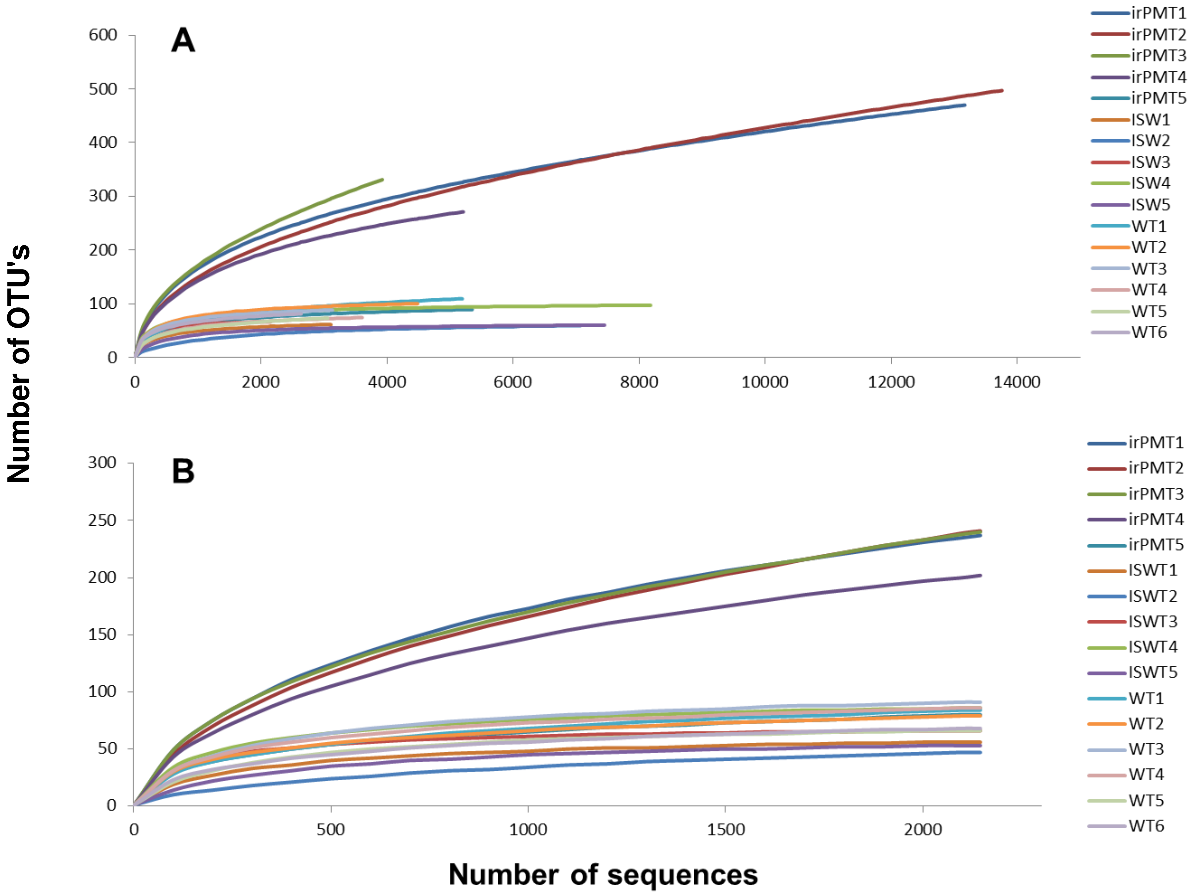
A
B
Number of OTU's
Number of sequences
0
100
200
300
400
500
600
0
2000
4000
6000
8000
10000
12000
14000
irPMT1
irPMT2
irPMT3
irPMT4
irPMT5
ISW1
ISW2
ISW3
ISW4
ISW5
WT1
WT2
WT3
WT4
WT5
WT6
0
50
100
150
200
250
300
0
500
1000
1500
2000
irPMT1
irPMT2
irPMT3
irPMT4
irPMT5
ISWT1
ISWT2
ISWT3
ISWT4
ISWT5
WT1
WT2
WT3
WT4
WT5
WT6

Table S1: OTUs taxonomic classifications and their abundance within each plant type

Legend: irPMT represent transformed *Nicotiana attenuata* samples, WT represent *Nicotiana attenuata* normal samples, ISWT represent *N. glauca* samples.

Sample name		irPMT	WT	ISWT	Taxonomy Classification
OTU No.	OTU Size				
1	5856	2004	2974	878	Bacteria(100);Firmicutes(100);Bacilli(100);Lactobacillales(100);Streptococcaceae(100);Streptococcus(100);
2	1126	522	464	140	Bacteria(100);"Proteobacteria"(100);Betaproteobacteria(100);Neisseriales(100);Neisseriaceae(100);Neisseria(100);
3	2511	1065	1122	324	Bacteria(100);Firmicutes(100);Bacilli(100);Bacillales(100);Staphylococcaceae(100);Staphylococcus(100);
4	684	246	311	127	Bacteria(100);"Fusobacteria"(100);"Fusobacteria"(100);"Fusobacteriales"(100);"Fusobacteriaceae"(100);Fusobacterium(100);
5	908	321	488	99	Bacteria(100);"Actinobacteria"(100);Actinobacteria(100);Actinomycetales(100);Actinomycetaceae(100);Actinomyces(100);
6	1368	285	1004	79	Bacteria(100);"Actinobacteria"(100);Actinobacteria(100);Actinomycetales(100);Micrococcaceae(100);Rothia(100);
7	662	192	434	36	Bacteria(100);"Actinobacteria"(100);Actinobacteria(100);Actinomycetales(100);Corynebacteriaceae(100);Corynebacterium(99);
8	567	191	292	84	Bacteria(100);"Actinobacteria"(100);Actinobacteria(100);Actinomycetales(100);Corynebacteriaceae(100);Corynebacterium(100);
9	682	167	164	351	Bacteria(100);"Actinobacteria"(100);Actinobacteria(100);Actinomycetales(100);Corynebacteriaceae(100);Corynebacterium(100);
10	458	207	170	81	Bacteria(100);"Proteobacteria"(100);Gammaproteobacteria(100);Pasteurellales(100);Pasteurellaceae(100);unclassified(92);
11	289	100	114	75	Bacteria(100);"Proteobacteria"(100);Gammaproteobacteria(100);Pseudomonadales(100);Moraxellaceae(100);Acinetobacter(100);
12	669	134	81	454	Bacteria(100);"Proteobacteria"(100);Gammaproteobacteria(100);"Enterobacteriales"(100);Enterobacteriaceae(100);unclassified(96);
13	98	65	33	0	Bacteria(100);"Proteobacteria"(100);Gammaproteobacteria(100);unclassified(83);unclassified(83);unclassified(83);
14	334	124	194	16	Bacteria(100);Firmicutes(100);Clostridia(100);Clostridiales(100);unclassified(100);unclassified(100);
15	401	136	143	122	Bacteria(100);"Actinobacteria"(100);Actinobacteria(100);Actinomycetales(100);Micrococcaceae(100);Micrococcus(98);
16	176	85	51	40	Bacteria(100);Firmicutes(100);Negativicutes(100);Selenomonadales(100);Veillonellaceae(100);Veillonella(100);
17	214	172	42	0	Bacteria(100);"Actinobacteria"(100);Actinobacteria(100);Bifidobacteriales(100);Bifidobacteriaceae(100);Gardnerella(100);
18	802	239	205	358	Bacteria(100);Firmicutes(100);Clostridia(100);Clostridiales(100);Clostridiales_Incertae_Sedis_XI(100);Finegoldia(100);

19	220	110	39	71	Bacteria(100);Firmicutes(100);Clostridia(100);Clostridiales(100);Clostridiales_Incertae_Sedis_XI(100);Anaerococcus(100);
20	455	225	223	7	Bacteria(100);"Proteobacteria"(100);Gammaproteobacteria(100);"Enterobacteriales"(100);Enterobacteriaceae(100);unclassified(100);
21	489	159	192	138	Bacteria(100);Firmicutes(100);Bacilli(100);Bacillales(100);Bacillales_Incertae_Sedis_XI(100);Gemella(100);
22	287	106	131	50	Bacteria(100);Firmicutes(100);Bacilli(100);Lactobacillales(100);Carnobacteriaceae(78);unclassified(56);
23	242	129	85	28	Bacteria(100);"Actinobacteria"(100);Actinobacteria(100);Actinomycetales(100);Micrococcaceae(100);Rothia(100);
24	233	76	65	92	Bacteria(100);Firmicutes(100);Clostridia(100);Clostridiales(100);Clostridiales_Incertae_Sedis_XI(100);Peptoniphilus(100);
25	219	111	75	33	Bacteria(100);"Proteobacteria"(100);Betaproteobacteria(100);Neisseriales(100);Neisseriaceae(100);unclassified(55);
26	113	40	48	25	Bacteria(100);"Actinobacteria"(100);Actinobacteria(100);Actinomycetales(100);Actinomycetaceae(100);Actinomyces(100);
27	176	46	40	90	Bacteria(100);"Proteobacteria"(100);Gammaproteobacteria(100);Pseudomonadales(100);Pseudomonadaceae(100);Pseudomonas(91);
28	204	117	18	69	Bacteria(100);"Proteobacteria"(100);Gammaproteobacteria(100);Pseudomonadales(100);Pseudomonadaceae(100);Pseudomonas(97);
29	190	96	40	54	Bacteria(100);"Actinobacteria"(100);Actinobacteria(100);Actinomycetales(100);Corynebacteriaceae(100);Corynebacterium(99);
30	180	58	79	43	Bacteria(100);"Bacteroidetes"(100);"Bacteroidia"(100);"Bacteroidales"(100);"Porphyromonadaceae"(100);Porphyromonas(93);
31	151	73	25	53	Bacteria(100);Firmicutes(100);Clostridia(100);Clostridiales(100);Clostridiales_Incertae_Sedis_XI(100);Anaerococcus(100);
32	98	33	60	5	Bacteria(100);Firmicutes(100);Negativicutes(100);Selenomonadales(100);Veillonellaceae(100);Selenomonas(66);
33	224	70	111	43	Bacteria(100);"Actinobacteria"(100);Actinobacteria(100);Actinomycetales(100);unclassified(100);unclassified(100);
34	166	125	41	0	Bacteria(100);Firmicutes(100);Bacilli(100);Lactobacillales(100);Lactobacillaceae(100);Lactobacillus(100);
35	66	34	13	19	Bacteria(100);"Actinobacteria"(100);Actinobacteria(100);Actinomycetales(100);Corynebacteriaceae(100);Corynebacterium(100);
36	75	40	25	10	Bacteria(100);"Fusobacteria"(100);"Fusobacteria"(100);"Fusobacteriales"(100);"Leptotrichiaceae"(100);Leptotrichia(100);
37	115	56	59	0	Bacteria(100);unclassified(100);unclassified(100);unclassified(100);unclassified(100);unclassified(100);
38	128	38	34	56	Bacteria(100);"Proteobacteria"(100);Gammaproteobacteria(100);Pseudomonadales(100);Moraxellaceae(100);Acinetobacter(100);
39	1586	35	1543	8	Bacteria(100);"Proteobacteria"(100);Gammaproteobacteria(100);"Enterobacteriales"(100);Enterobacteriaceae(100);unclassified(100);
40	159	44	47	68	Bacteria(100);"Bacteroidetes"(100);unclassified(71);unclassified(71);unclassified(71);unclassified(71);
41	127	39	31	57	Bacteria(100);"Proteobacteria"(100);Gammaproteobacteria(100);Oceanospirillales(100);Halomonadaceae(100);Halomonas(99);
42	148	77	61	10	Bacteria(100);Firmicutes(100);Bacilli(100);Lactobacillales(100);Streptococcaceae(100);Streptococcus(100);

43	191	49	34	108	Bacteria(100);"Proteobacteria"(100);Gammaproteobacteria(100);Pasteurellales(100);Pasteurellaceae(100);unclassified(100);
44	72	42	28	2	Bacteria(100);"Fusobacteria"(100);"Fusobacteria"(100);"Fusobacteriales"(100);"Leptotrichiaceae"(100);Leptotrichia(100);
45	112	39	53	20	Bacteria(100);"Actinobacteria"(100);Actinobacteria(100);Actinomycetales(100);Propionibacteriaceae(100);Propionibacterium(100);
46	63	28	32	3	Bacteria(100);"Bacteroidetes"(100);Flavobacteria(100);"Flavobacteriales"(100);Flavobacteriaceae(100);Capnocytophaga(100);
47	186	21	150	15	Bacteria(100);"Proteobacteria"(100);Alphaproteobacteria(100);Rhodobacterales(100);Rhodobacteraceae(100);Paracoccus(81);
48	63	41	22	0	Bacteria(100);"Proteobacteria"(100);Betaproteobacteria(100);Neisseriales(100);Neisseriaceae(100);unclassified(95);
49	139	66	45	28	Bacteria(100);Firmicutes(100);Bacilli(100);Lactobacillales(100);Carnobacteriaceae(100);Granulicatella(100);
50	72	29	23	20	Bacteria(100);Firmicutes(100);Clostridia(100);Clostridiales(100);Clostridiales_Incertae_Sedis_XI(100);Anaerococcus(100);
51	173	49	40	84	Bacteria(100);"Proteobacteria"(100);Betaproteobacteria(100);Burkholderiales(100);Comamonadaceae(100);Pelomonas(98);
52	142	89	31	22	Bacteria(100);Firmicutes(100);Bacilli(100);Lactobacillales(100);Streptococcaceae(100);Lactococcus(100);
53	56	27	17	12	Bacteria(100);"Proteobacteria"(100);Gammaproteobacteria(100);Oceanospirillales(100);Halomonadaceae(100);Halomonas(89);
54	115	31	36	48	Bacteria(100);unclassified(55);unclassified(55);unclassified(55);unclassified(55);unclassified(55);
55	84	33	11	40	Bacteria(100);"Actinobacteria"(100);Actinobacteria(100);Actinomycetales(100);Corynebacteriaceae(81);Corynebacterium(81);
56	60	33	23	4	Bacteria(100);"Bacteroidetes"(100);"Bacteroidia"(100);"Bacteroidales"(100);"Porphyromonadaceae"(100);Porphyromonas(100);
57	39	23	16	0	Bacteria(100);"Proteobacteria"(100);Epsilonproteobacteria(100);Campylobacterales(100);Campylobacteraceae(100);Campylobacter(100);
58	89	44	17	28	Bacteria(100);"Bacteroidetes"(100);Flavobacteria(100);"Flavobacteriales"(100);Flavobacteriaceae(100);Capnocytophaga(100);
59	43	30	12	1	Bacteria(100);"Proteobacteria"(100);Gammaproteobacteria(100);Cardiobacteriales(100);Cardiobacteriaceae(100);Cardiobacterium(100);
60	46	20	24	2	Bacteria(100);"Bacteroidetes"(100);"Bacteroidia"(100);"Bacteroidales"(100);"Prevotellaceae"(100);Prevotella(100);
61	37	23	13	1	Bacteria(100);"Bacteroidetes"(100);"Bacteroidia"(100);"Bacteroidales"(100);"Prevotellaceae"(100);Prevotella(100);
62	50	31	19	0	Bacteria(100);"Bacteroidetes"(100);"Bacteroidia"(100);"Bacteroidales"(100);"Prevotellaceae"(100);Prevotella(100);
63	101	41	26	34	Bacteria(100);Firmicutes(100);Clostridia(100);Clostridiales(100);Peptostreptococcaceae(100);Peptostreptococcus(100);
64	27	17	10	0	Bacteria(100);unclassified(100);unclassified(100);unclassified(100);unclassified(100);unclassified(100);
65	100	90	0	10	Bacteria(100);"Bacteroidetes"(100);Flavobacteria(100);"Flavobacteriales"(100);Flavobacteriaceae(100);Chryseobacterium(100);
66	52	38	5	9	Bacteria(100);"Actinobacteria"(100);Actinobacteria(100);Actinomycetales(100);Micrococcaceae(98);unclassified(60);
67	215	17	198	0	Bacteria(100);Firmicutes(100);Bacilli(100);Lactobacillales(100);Enterococcaceae(99);Enterococcus(98);

68	40	16	20	4	Bacteria(100);Firmicutes(100);Negativicutes(100);Selenomonadales(100);Veillonellaceae(100);Dialister(100);
69	56	35	18	3	Bacteria(100);Firmicutes(100);Clostridia(100);Clostridiales(100);Clostridiales_Incertae_Sedis_XI(100);Peptoniphilus(100);
70	45	21	22	2	Bacteria(100);Firmicutes(100);Clostridia(100);Clostridiales(100);unclassified(100);unclassified(100);
71	19	10	2	7	Bacteria(100);"Proteobacteria"(100);Alphaproteobacteria(100);Caulobacterales(100);Caulobacteraceae(100);Brevundimonas(100);
72	31	19	6	6	Bacteria(100);"Bacteroidetes"(100);"Bacteroidia"(100);"Bacteroidales"(100);"Porphyromonadaceae"(100);Porphyromonas(100);
73	36	18	13	5	Bacteria(100);Firmicutes(100);Bacilli(100);Lactobacillales(100);Aerococcaceae(95);Abiotrophia(95);
74	77	16	5	56	Bacteria(100);Firmicutes(100);Bacilli(100);Lactobacillales(100);Lactobacillaceae(100);Lactobacillus(97);
75	149	20	28	101	Bacteria(100);"Proteobacteria"(100);Gammaproteobacteria(100);Pseudomonadales(100);Moraxellaceae(100);Enhydrobacter(100);
76	36	21	15	0	Bacteria(100);"Proteobacteria"(100);Betaproteobacteria(100);Neisseriales(100);Neisseriaceae(100);Kingella(100);
77	5435	11	4	###	Bacteria(100);"Proteobacteria"(100);Gammaproteobacteria(100);"Enterobacteriales"(100);Enterobacteriaceae(100);unclassified(100<br>);
78	17	14	2	1	Bacteria(100);"Bacteroidetes"(100);"Bacteroidia"(100);"Bacteroidales"(100);"Prevotellaceae"(100);Prevotella(100);
79	19	13	6	0	Bacteria(100);"Actinobacteria"(100);Actinobacteria(100);Actinomycetales(100);Actinomycetaceae(100);Actinomyces(100);
80	10	8	2	0	Bacteria(100);"Fusobacteria"(100);"Fusobacteria"(100);"Fusobacteriales"(100);"Leptotrichiaceae"(100);Leptotrichia(100);
81	29	20	9	0	Bacteria(100);Firmicutes(100);Clostridia(100);Clostridiales(100);Lachnospiraceae(100);unclassified(100);
82	74	15	18	41	Bacteria(100);Firmicutes(100);Negativicutes(100);Selenomonadales(100);Veillonellaceae(100);Veillonella(92);
83	10	9	0	1	Bacteria(100);"Proteobacteria"(100);Alphaproteobacteria(100);Sphingomonadales(100);Sphingomonadaceae(100);Sphingomonas(9<br>1);
84	291	46	198	47	Bacteria(100);"Proteobacteria"(100);Gammaproteobacteria(100);Aeromonadales(100);Aeromonadaceae(100);Aeromonas(100);
85	24	10	14	0	Bacteria(100);"Actinobacteria"(100);Actinobacteria(100);Actinomycetales(100);Actinomycetaceae(100);Actinomyces(100);
86	26	10	4	12	Bacteria(100);"Proteobacteria"(100);Gammaproteobacteria(100);Alteromonadales(100);Alteromonadaceae(100);Marinobacter(100)<br>;
87	20	8	2	10	Bacteria(100);"Proteobacteria"(100);Betaproteobacteria(100);Burkholderiales(100);Oxalobacteraceae(100);Naxibacter(52);
88	54	24	25	5	Bacteria(100);"Actinobacteria"(100);Actinobacteria(100);Actinomycetales(100);Dietziaceae(100);Dietzia(100);
89	29	22	6	1	Bacteria(100);Firmicutes(100);Clostridia(100);Clostridiales(100);unclassified(100);unclassified(100);
90	14	14	0	0	Bacteria(100);"Proteobacteria"(100);Gammaproteobacteria(100);Cardiobacteriales(100);Cardiobacteriaceae(100);Cardiobacterium(1<br>00);
91	32	19	10	3	Bacteria(100);Firmicutes(100);Clostridia(100);Clostridiales(100);Clostridiales_Incertae_Sedis_XI(100);Anaerococcus(100);

92	25	11	2	12	Bacteria(100);"Bacteroidetes"(100);Flavobacteria(100);"Flavobacteriales"(100);Flavobacteriaceae(100);Capnocytophaga(100);
93	15	12	3	0	Bacteria(100);"Actinobacteria"(100);Actinobacteria(100);Actinomycetales(100);Intrasporangiaceae(100);Janibacter(59);
94	41	28	4	9	Bacteria(100);Firmicutes(96);unclassified(96);unclassified(96);unclassified(96);unclassified(96);
95	9	9	0	0	Bacteria(100);Firmicutes(100);Clostridia(100);Clostridiales(100);unclassified(100);unclassified(100);
96	33	20	7	6	Bacteria(100);Firmicutes(100);Clostridia(100);Clostridiales(100);Clostridiales_Incertae_Sedis_XI(100);Peptoniphilus(100);
97	19	9	0	10	Bacteria(100);"Proteobacteria"(100);Alphaproteobacteria(100);Rhizobiales(100);Bradyrhizobiaceae(100);Bosea(100);
98	8	8	0	0	Bacteria(100);Firmicutes(100);Clostridia(100);Clostridiales(100);unclassified(70);unclassified(70);
99	11	10	1	0	Bacteria(100);Firmicutes(100);Clostridia(100);Clostridiales(100);Lachnospiraceae(100);unclassified(100);
100	27	17	7	3	Bacteria(100);"Bacteroidetes"(100);"Bacteroidia"(100);"Bacteroidales"(100);"Porphyromonadaceae"(100);Porphyromonas(100);
101	8	7	1	0	Bacteria(100);"Bacteroidetes"(100);"Bacteroidia"(100);"Bacteroidales"(100);"Prevotellaceae"(100);Prevotella(100);
102	24	13	5	6	Bacteria(100);"Actinobacteria"(100);Actinobacteria(100);Actinomycetales(100);Brevibacteriaceae(100);Brevibacterium(100);
103	30	18	10	2	Bacteria(100);"Bacteroidetes"(100);"Bacteroidia"(100);"Bacteroidales"(100);"Prevotellaceae"(100);Prevotella(100);
104	5	5	0	0	Bacteria(100);TM7(100);TM7_class_incertae_sedis(100);TM7_order_incertae_sedis(100);TM7_family_incertae_sedis(100);TM7_genus_incertae_sedis(100);
105	38	6	32	0	Bacteria(100);Firmicutes(100);Bacilli(100);Lactobacillales(100);Leuconostocaceae(100);Weissella(100);
106	12	11	1	0	Bacteria(100);Firmicutes(100);Bacilli(100);Lactobacillales(100);Leuconostocaceae(100);Leuconostoc(100);
107	7	6	0	1	Bacteria(100);SR1(100);SR1_class_incertae_sedis(100);SR1_order_incertae_sedis(100);SR1_family_incertae_sedis(100);SR1_genus_incertae_sedis(100);
108	16	9	4	3	Bacteria(100);"Fusobacteria"(100);"Fusobacteria"(100);"Fusobacteriales"(100);"Leptotrichiaceae"(100);Leptotrichia(100);
109	10	5	3	2	Bacteria(100);"Bacteroidetes"(100);"Bacteroidia"(70);"Bacteroidales"(70);unclassified(70);unclassified(70);
110	14	6	7	1	Bacteria(100);Firmicutes(100);Clostridia(100);Clostridiales(100);unclassified(100);unclassified(100);
111	12	9	3	0	Bacteria(100);Firmicutes(100);Clostridia(100);Clostridiales(100);Peptostreptococcaceae(100);Peptostreptococcaceae_incertae_sedis(100);
112	3	3	0	0	Bacteria(100);"Fusobacteria"(100);"Fusobacteria"(100);"Fusobacteriales"(100);"Fusobacteriaceae"(100);unclassified(95);
113	15	8	7	0	Bacteria(100);Firmicutes(100);Clostridia(100);Clostridiales(100);Incertae_Sedis_XI(100);Murdochiella(100);
114	5	5	0	0	Bacteria(100);"Bacteroidetes"(100);Flavobacteria(100);"Flavobacteriales"(100);Flavobacteriaceae(100);Capnocytophaga(100);
115	8	8	0	0	Bacteria(100);"Proteobacteria"(100);Epsilonproteobacteria(100);Campylobacterales(100);Campylobacteraceae(100);Campylobacter(100);
116	18	8	1	9	Bacteria(100);"Proteobacteria"(100);Betaproteobacteria(100);Burkholderiales(100);unclassified(100);unclassified(100);
117	10	9	0	1	Bacteria(100);"Proteobacteria"(100);Betaproteobacteria(100);Neisseriales(100);Neisseriaceae(100);Neisseria(100);

118	8	4	4	0	Bacteria(100);"Actinobacteria"(100);Actinobacteria(100);Actinomycetales(100);Corynebacteriaceae(52);Corynebacterium(52);
119	7	7	0	0	Bacteria(100);Firmicutes(100);Clostridia(100);Clostridiales(100);unclassified(79);unclassified(79);
120	13	6	2	5	Bacteria(100);"Proteobacteria"(100);Gammaproteobacteria(100);Pseudomonadales(100);Pseudomonadaceae(100);unclassified;
121	10	7	1	2	Bacteria(100);"Proteobacteria"(100);Alphaproteobacteria(100);Caulobacterales(100);Caulobacteraceae(100);Brevundimonas(100);
122	12	11	1	0	Bacteria(100);"Proteobacteria"(100);Betaproteobacteria(100);Burkholderiales(100);Comamonadaceae(100);unclassified;
123	12	9	3	0	Bacteria(100);TM7(100);TM7_class_incertae_sedis(100);TM7_order_incertae_sedis(100);TM7_family_incertae_sedis(100);TM7_genus_incertae_sedis(100);
124	6	6	0	0	Bacteria(100);Firmicutes(100);Clostridia(100);Clostridiales(100);Lachnospiraceae(100);unclassified(100);
125	10	6	1	3	Bacteria(100);"Proteobacteria"(100);Alphaproteobacteria(100);Sphingomonadales(100);Sphingomonadaceae(100);Sphingomonas(100);
126	11	7	0	4	Bacteria(100);Firmicutes(100);Negativicutes(100);Selenomonadales(100);Veillonellaceae(100);Negativicoccus(100);
127	8	4	4	0	Bacteria(100);"Bacteroidetes"(100);"Bacteroidia"(100);"Bacteroidales"(100);"Porphyromonadaceae"(100);Tannerella(100);
128	6	6	0	0	Bacteria(100);Firmicutes(100);Clostridia(100);Clostridiales(100);Ruminococcaceae(100);Butyricicoccus(100);
129	4	3	1	0	Bacteria(100);"Actinobacteria"(100);Actinobacteria(100);Actinomycetales(100);Dermabacteraceae(100);Brachybacterium(100);
130	11	10	1	0	Bacteria(100);"Bacteroidetes"(100);"Bacteroidia"(100);"Bacteroidales"(100);"Porphyromonadaceae"(100);unclassified(86);
131	8	8	0	0	Bacteria(100);Firmicutes(100);Clostridia(100);Clostridiales(100);Clostridiales_Incertae_Sedis_XI(100);Anaerococcus(100);
132	151	19	37	95	Bacteria(100);"Proteobacteria"(100);Gammaproteobacteria(100);"Vibrionales"(100);Vibrionaceae(100);Vibrio(94);
133	8	8	0	0	Bacteria(100);"Actinobacteria"(100);Actinobacteria(100);Actinomycetales(100);Corynebacteriaceae(100);Corynebacterium(95);
134	2	2	0	0	Bacteria(100);"Proteobacteria"(100);Alphaproteobacteria(100);Rhizobiales(100);Rhizobiaceae(100);Rhizobium(100);
135	4	2	2	0	Bacteria(100);"Proteobacteria"(100);Gammaproteobacteria(100);Pseudomonadales(100);Moraxellaceae(100);Moraxella(100);
136	5	5	0	0	Bacteria(100);"Bacteroidetes"(100);unclassified(100);unclassified(100);unclassified(100);unclassified(100);
137	5	5	0	0	Bacteria(100);Firmicutes(100);Clostridia(100);Clostridiales(100);Eubacteriaceae(100);Eubacterium(100);
138	9	9	0	0	Bacteria(100);"Bacteroidetes"(82);unclassified(82);unclassified(82);unclassified(82);unclassified(82);
139	6	6	0	0	Bacteria(100);"Bacteroidetes"(100);"Bacteroidia"(100);"Bacteroidales"(100);"Prevotellaceae"(100);Prevotella(100);
140	14	10	4	0	Bacteria(100);"Bacteroidetes"(100);"Bacteroidia"(100);"Bacteroidales"(100);"Prevotellaceae"(100);Prevotella(100);
141	4	4	0	0	Bacteria(100);"Actinobacteria"(100);Actinobacteria(100);Actinomycetales(100);Nocardiaceae(100);Gordonia(100);
142	6	6	0	0	Bacteria(100);"Proteobacteria"(100);Betaproteobacteria(100);Neisseriales(100);Neisseriaceae(100);Neisseria(81);
143	6	6	0	0	Bacteria(100);Firmicutes(100);Bacilli(100);Bacillales(100);Planococcaceae(100);unclassified(100);

144	5	4	0	1	Bacteria(100);"Actinobacteria"(100);Actinobacteria(100);Actinomycetales(100);Nocardioidaceae(100);unclassified(52);
145	6	4	0	2	Bacteria(100);"Proteobacteria"(100);Betaproteobacteria(100);Burkholderiales(100);Comamonadaceae(100);unclassified(57);
146	1	1	0	0	Bacteria(100);TM7(100);TM7_class_incertae_sedis(100);TM7_order_incertae_sedis(100);TM7_family_incertae_sedis(100);TM7_genus_incertae_sedis(100);
147	5	5	0	0	Bacteria(100);Firmicutes(100);Clostridia(100);Clostridiales(100);unclassified(100);unclassified(100);
148	2	2	0	0	Bacteria(100);unclassified(100);unclassified(100);unclassified(100);unclassified(100);unclassified(100);
149	8	4	4	0	Bacteria(100);Firmicutes(100);Clostridia(100);Clostridiales(100);Clostridiales_Incertae_Sedis_XI(100);Parvimonas(100);
150	5	4	0	1	Bacteria(100);"Bacteroidetes"(100);"Bacteroidia"(100);"Bacteroidales"(100);"Prevotellaceae"(100);Prevotella(100);
151	3	2	0	1	Bacteria(100);"Bacteroidetes"(100);"Bacteroidia"(100);"Bacteroidales"(100);"Prevotellaceae"(67);unclassified(67);
152	1	1	0	0	Bacteria(100);"Bacteroidetes"(100);"Sphingobacteria"(100);"Sphingobacteriales"(100);Sphingobacteriaceae(100);Pedobacter(100);
153	6	6	0	0	Bacteria(100);Firmicutes(100);Bacilli(100);Lactobacillales(100);Aerococcaceae(94);Facklamia(94);
154	3	3	0	0	Bacteria(100);"Bacteroidetes"(100);Flavobacteria(100);"Flavobacteriales"(100);Flavobacteriaceae(100);Chryseobacterium(94);
155	2	2	0	0	Bacteria(100);Firmicutes(91);unclassified;unclassified;unclassified;unclassified;
156	2	2	0	0	Bacteria(100);Firmicutes(100);Negativicutes(100);Selenomonadales(100);Veillonellaceae(100);unclassified(100);
157	2	2	0	0	Bacteria(100);"Proteobacteria"(100);Alphaproteobacteria(100);Rhizobiales(100);Hyphomicrobiaceae(100);Devosia(100);
158	6	4	2	0	Bacteria(100);Firmicutes(100);Bacilli(100);Lactobacillales(100);Streptococcaceae(100);Streptococcus(100);
159	7	5	0	2	Bacteria(100);Firmicutes(100);Bacilli(100);Lactobacillales(100);Lactobacillaceae(100);unclassified(70);
160	3	3	0	0	Bacteria(100);Firmicutes(100);Clostridia(100);Clostridiales(100);Lachnospiraceae(100);Johnsonella(100);
161	9	7	0	2	Bacteria(100);"Proteobacteria"(100);Alphaproteobacteria(100);Rhodobacterales(100);Rhodobacteraceae(100);Paracoccus(70);
162	6	5	1	0	Bacteria(100);Firmicutes(100);Bacilli(100);Bacillales(89);unclassified;unclassified;
163	5	5	0	0	Bacteria(100);"Bacteroidetes"(100);"Sphingobacteria"(100);"Sphingobacteriales"(100);Sphingobacteriaceae(100);Sphingobacterium(100);
164	7	7	0	0	Bacteria(100);"Actinobacteria"(100);Actinobacteria(100);Actinomycetales(100);Geodermatophilaceae(100);Modestobacter(100);
165	7	6	1	0	Bacteria(100);"Actinobacteria"(100);Actinobacteria(100);Coriobacteriales(100);Coriobacteriaceae(100);Atopobium(100);
166	6	6	0	0	Bacteria(100);"Bacteroidetes"(100);unclassified(100);unclassified(100);unclassified(100);unclassified(100);
167	2	2	0	0	Bacteria(100);"Proteobacteria"(100);Gammaproteobacteria(100);Alteromonadales(100);Alteromonadaceae(100);Marinobacter(100);
168	2	2	0	0	Bacteria(100);"Fusobacteria"(100);"Fusobacteria"(100);"Fusobacteriales"(100);"Fusobacteriaceae"(100);Fusobacterium(100);
169	11	6	1	4	Bacteria(100);Firmicutes(100);Clostridia(100);Clostridiales(100);Lachnospiraceae(100);unclassified(100);
170	5	4	1	0	Bacteria(100);"Bacteroidetes"(100);"Bacteroidia"(100);"Bacteroidales"(100);"Porphyromonadaceae"(100);Porphyromonas(100);

171	4	4	0	0	Bacteria(100);"Actinobacteria"(100);Actinobacteria(100);Actinomycetales(100);Actinomycetaceae(100);Mobiluncus(100);
172	4	4	0	0	Bacteria(100);"Bacteroidetes"(100);"Sphingobacteria"(100);"Sphingobacteriales"(100);Chitinophagaceae(100);Sediminibacterium(100);
173	7	3	1	3	Bacteria(100);Firmicutes(100);Clostridia(100);Clostridiales(100);Clostridiaceae_1(100);Clostridium_sensu_stricto(83);
174	6	5	1	0	Bacteria(100);"Bacteroidetes"(100);"Bacteroidia"(100);"Bacteroidales"(100);"Prevotellaceae"(100);Prevotella(100);
175	1	1	0	0	Bacteria(100);Firmicutes(100);Bacilli(100);Lactobacillales(100);Carnobacteriaceae(92);Trichococcus(92);
176	2	2	0	0	Bacteria(100);unclassified(100);unclassified(100);unclassified(100);unclassified(100);unclassified(100);
177	2	2	0	0	Bacteria(100);"Bacteroidetes"(100);"Sphingobacteria"(100);"Sphingobacteriales"(100);Chitinophagaceae(100);unclassified(100);
178	3	3	0	0	Bacteria(100);"Bacteroidetes"(100);Flavobacteria(100);"Flavobacteriales"(100);Flavobacteriaceae(100);Epilithonimonas(100);
179	4	4	0	0	Bacteria(100);"Bacteroidetes"(100);"Bacteroidia"(100);"Bacteroidales"(100);"Prevotellaceae"(100);Prevotella(100);
180	3	3	0	0	Bacteria(100);"Proteobacteria"(100);Gammaproteobacteria(100);Xanthomonadales(100);Xanthomonadaceae(100);unclassified(100);
181	5	4	1	0	Bacteria(100);Firmicutes(100);Negativicutes(100);Selenomonadales(100);Veillonellaceae(100);Selenomonas(100);
182	2	2	0	0	Bacteria(100);"Fusobacteria"(100);"Fusobacteria"(100);"Fusobacteriales"(100);"Leptotrichiaceae"(100);Leptotrichia(100);
183	2	2	0	0	Bacteria(100);"Bacteroidetes"(100);"Bacteroidia"(100);"Bacteroidales"(100);"Prevotellaceae"(100);Prevotella(100);
184	4	3	0	1	Bacteria(100);"Actinobacteria"(100);Actinobacteria(100);Actinomycetales(100);Geodermatophilaceae(100);Blastococcus(100);
185	5	4	1	0	Bacteria(100);"Actinobacteria"(100);Actinobacteria(100);Actinomycetales(100);Microbacteriaceae(100);unclassified(82);
186	6	6	0	0	Bacteria(100);"Spirochaetes"(100);Spirochaetes(100);Spirochaetales(100);Spirochaetaceae(100);Treponema(100);
187	3	3	0	0	Bacteria(100);"Proteobacteria"(100);Alphaproteobacteria(100);Sphingomonadales(100);Sphingomonadaceae(100);Sphingomonas(100);
188	3	3	0	0	Bacteria(100);"Tenericutes"(100);Mollicutes(100);Mycoplasmatales(100);Mycoplasmataceae(100);Ureaplasma(100);
189	4	2	0	2	Bacteria(100);"Actinobacteria"(100);Actinobacteria(100);Actinomycetales(100);Micrococcaceae(100);Kocuria(70);
190	2	2	0	0	Bacteria(100);"Actinobacteria"(100);Actinobacteria(100);Coriobacteriales(100);Coriobacteriaceae(100);Collinsella(100);
191	1	1	0	0	Bacteria(100);"Proteobacteria"(100);Alphaproteobacteria(100);Rhizobiales(100);"Aurantimonadaceae"(100);Aurantimonas(100);
192	3	3	0	0	Bacteria(100);Firmicutes(100);Clostridia(100);Clostridiales(100);unclassified(100);unclassified(100);
193	5	5	0	0	Bacteria(100);"Proteobacteria"(100);Epsilonproteobacteria(100);Campylobacterales(100);Campylobacteraceae(100);Campylobacter(100);
194	2	2	0	0	Bacteria(100);"Actinobacteria"(100);Actinobacteria(100);Actinomycetales(100);Nocardioidaceae(100);Aeromicrobium(100);
195	3	3	0	0	Bacteria(100);Firmicutes(100);Clostridia(100);Clostridiales(100);Ruminococcaceae(100);unclassified(100);
196	6	6	0	0	Bacteria(100);"Proteobacteria"(100);Betaproteobacteria(53);Burkholderiales(53);Comamonadaceae(53);Rhodoferax(53);

197	5	5	0	0	Bacteria(100);"Proteobacteria"(100);Alphaproteobacteria(100);Rhizobiales(100);Rhizobiaceae(56);unclassified(56);
198	3	2	1	0	Bacteria(100);Firmicutes(100);Bacilli(100);Lactobacillales(100);Leuconostocaceae(100);Leuconostoc(100);
199	2	2	0	0	Bacteria(100);Firmicutes(100);Clostridia(100);Clostridiales(100);Lachnospiraceae(100);Roseburia(100);
200	1	1	0	0	Bacteria(100);"Bacteroidetes"(100);"Bacteroidia"(100);"Bacteroidales"(100);"Porphyromonadaceae"(100);Tannerella(100);
201	1	1	0	0	Bacteria(100);Firmicutes(100);Clostridia(100);Clostridiales(100);Lachnospiraceae(100);unclassified(100);
202	2	2	0	0	Bacteria(100);"Actinobacteria"(100);Actinobacteria(100);Actinomycetales(100);Dietziaceae(100);Dietzia(100);
203	6	2	2	2	Bacteria(100);Firmicutes(100);Clostridia(100);Clostridiales(100);Peptostreptococcaceae(100);Filifactor(100);
204	9	7	0	2	Bacteria(100);"Bacteroidetes"(100);Flavobacteria(100);"Flavobacteriales"(100);Flavobacteriaceae(100);Flavobacterium(100);
205	1	1	0	0	Bacteria(100);TM7(100);TM7_class_incertae_sedis(100);TM7_order_incertae_sedis(100);TM7_family_incertae_sedis(100);TM7_genus_incertae_sedis(100);
206	1	1	0	0	Bacteria(100);"Actinobacteria"(100);Actinobacteria(100);Actinomycetales(100);Microbacteriaceae(100);unclassified(100);
207	1	1	0	0	Bacteria(100);unclassified(100);unclassified(100);unclassified(100);unclassified(100);unclassified(100);
208	7	7	0	0	Bacteria(100);"Proteobacteria"(100);Betaproteobacteria(100);Burkholderiales(100);Burkholderiaceae(100);Cupriavidus(100);
209	9	7	0	2	Bacteria(100);"Proteobacteria"(100);Gammaproteobacteria(100);Pseudomonadales(100);Pseudomonadaceae(100);Cellvibrio(100);
210	2	2	0	0	Bacteria(100);"Bacteroidetes"(100);"Sphingobacteria"(100);"Sphingobacteriales"(100);Chitinophagaceae(100);unclassified(100);
211	2	2	0	0	Bacteria(100);"Proteobacteria"(100);Betaproteobacteria(100);Burkholderiales(100);Burkholderiaceae(100);Ralstonia(100);
212	10	10	0	0	Bacteria(100);"Proteobacteria"(100);Betaproteobacteria(100);Neisseriales(100);Neisseriaceae(100);Vogesella(100);
213	4	2	0	2	Bacteria(100);Firmicutes(100);Bacilli(100);Lactobacillales(100);Lactobacillaceae(100);Lactobacillus(100);
214	3	3	0	0	Bacteria(100);"Proteobacteria"(100);Epsilonproteobacteria(100);Campylobacterales(100);Campylobacteraceae(100);Campylobacter(100);
215	3	2	1	0	Bacteria(100);"Actinobacteria"(100);Actinobacteria(100);Actinomycetales(100);Dermacoccaceae(100);Kytococcus(100);
216	1	1	0	0	Bacteria(100);"Tenericutes"(100);Mollicutes(100);Mycoplasmatales(100);Mycoplasmataceae(100);Mycoplasma(100);
217	1	1	0	0	Bacteria(100);unclassified(100);unclassified(100);unclassified(100);unclassified(100);unclassified(100);
218	2	2	0	0	Bacteria(100);"Actinobacteria"(100);Actinobacteria(100);Actinomycetales(100);Nocardiaceae(100);Rhodococcus(100);
219	2	2	0	0	Bacteria(100);Firmicutes(100);Bacilli(100);Bacillales(100);Bacillaceae_1(84);unclassified;
220	3	3	0	0	Bacteria(100);"Actinobacteria"(100);Actinobacteria(100);Actinomycetales(100);Dermabacteraceae(100);Dermabacter(100);
221	1	1	0	0	Bacteria(100);"Spirochaetes"(100);Spirochaetes(100);Spirochaetales(100);Spirochaetaceae(100);Treponema(100);
222	3	3	0	0	Bacteria(100);"Bacteroidetes"(100);"Bacteroidia"(100);"Bacteroidales"(100);unclassified(86);unclassified(86);
223	3	1	2	0	Bacteria(100);"Proteobacteria"(100);Gammaproteobacteria(100);Xanthomonadales(100);Xanthomonadaceae(100);Luteimonas(100);

224	1	1	0	0	Bacteria(100);Firmicutes(100);Bacilli(100);unclassified(84);unclassified(84);unclassified(84);
225	2	2	0	0	Bacteria(100);"Proteobacteria"(100);Gammaproteobacteria(100);Xanthomonadales(100);Xanthomonadaceae(100);unclassified(100);
226	1	1	0	0	Bacteria(100);"Proteobacteria"(100);Gammaproteobacteria(100);unclassified(100);unclassified(100);unclassified(100);
227	12	4	0	8	Bacteria(100);"Bacteroidetes"(100);Flavobacteria(100);"Flavobacteriales"(100);Flavobacteriaceae(100);Cloacibacterium(100);
228	4	4	0	0	Bacteria(100);"Actinobacteria"(100);Actinobacteria(100);Actinomycetales(100);Dietziaceae(100);Dietzia(100);
229	1	1	0	0	Bacteria(100);Firmicutes(100);Clostridia(100);Clostridiales(100);Lachnospiraceae(100);Blautia(100);
230	1	1	0	0	Bacteria(100);"Bacteroidetes"(100);"Sphingobacteria"(100);"Sphingobacteriales"(100);Chitinophagaceae(100);Segetibacter(100);
231	4	4	0	0	Bacteria(100);"Proteobacteria"(100);Alphaproteobacteria(100);Rhizobiales(100);Methylobacteriaceae(100);Methylobacterium(100);
232	1	1	0	0	Bacteria(100);"Bacteroidetes"(100);"Sphingobacteria"(100);"Sphingobacteriales"(100);Sphingobacteriaceae(100);Sphingobacterium(100);
233	5	4	1	0	Bacteria(100);Firmicutes(100);Bacilli(100);Bacillales(100);Bacillales_Incertae_Sedis_XII(100);Exiguobacterium(100);
234	3	3	0	0	Bacteria(100);"Deinococcus-Thermus"(100);Deinococci(100);Deinococcales(100);Deinococcaceae(100);Deinococcus(100);
235	2	2	0	0	Bacteria(100);"Proteobacteria"(100);Betaproteobacteria(100);Burkholderiales(100);unclassified(67);unclassified(67);
236	4	4	0	0	Bacteria(100);"Proteobacteria"(100);Gammaproteobacteria(100);Pseudomonadales(100);Moraxellaceae(100);unclassified(100);
237	2	2	0	0	Bacteria(100);"Actinobacteria"(100);Actinobacteria(100);Actinomycetales(100);Actinomycetaceae(100);Varibaculum(100);
238	2	2	0	0	Bacteria(100);TM7(100);TM7_class_incertae_sedis(100);TM7_order_incertae_sedis(100);TM7_family_incertae_sedis(100);TM7_genus_incertae_sedis(100);
239	2	2	0	0	Bacteria(100);Firmicutes(100);Bacilli(100);Lactobacillales(100);unclassified(75);unclassified(75);
240	2	2	0	0	Bacteria(100);unclassified(100);unclassified(100);unclassified(100);unclassified(100);unclassified(100);
241	8	2	3	3	Bacteria(100);"Proteobacteria"(100);Gammaproteobacteria(100);Oceanospirillales(100);Oceanospirillaceae(100);Marinomonas(100);
242	2	2	0	0	Bacteria(100);"Proteobacteria"(100);Gammaproteobacteria(100);Pseudomonadales(75);unclassified;unclassified;
243	9	6	2	1	Bacteria(100);"Actinobacteria"(100);Actinobacteria(100);Actinomycetales(100);Actinomycetaceae(100);Actinomyces(100);
244	3	3	0	0	Bacteria(100);"Proteobacteria"(100);Alphaproteobacteria(100);Caulobacterales(100);Caulobacteraceae(100);Brevundimonas(100);
245	3	3	0	0	Bacteria(100);"Actinobacteria"(100);Actinobacteria(100);Actinomycetales(100);Micrococcaceae(100);Arthrobacter(88);
246	1	1	0	0	Bacteria(100);Firmicutes(100);Clostridia(100);Clostridiales(100);Lachnospiraceae(100);unclassified(100);
247	1	1	0	0	Bacteria(100);"Bacteroidetes"(100);"Bacteroidia"(100);"Bacteroidales"(100);"Prevotellaceae"(100);Prevotella(100);
248	3	3	0	0	Bacteria(100);"Deinococcus-Thermus"(100);Deinococci(100);Thermales(100);Thermaceae(100);Thermus(100);
249	1	1	0	0	Bacteria(100);"Bacteroidetes"(100);"Sphingobacteria"(100);"Sphingobacteriales"(100);Chitinophagaceae(100);unclassified(100);

250	1	1	0	0	Bacteria(100);unclassified(100);unclassified(100);unclassified(100);unclassified(100);unclassified(100);
251	1	1	0	0	Bacteria(100);Firmicutes(100);Bacilli(67);Bacillales(67);unclassified(67);unclassified(67);
252	2	2	0	0	Bacteria(100);Firmicutes(100);Negativicutes(100);Selenomonadales(100);Veillonellaceae(100);Selenomonas(100);
253	6	6	0	0	Bacteria(100);"Actinobacteria"(100);Actinobacteria(100);Actinomycetales(100);Corynebacteriaceae(100);Corynebacterium(100);
254	2	2	0	0	Bacteria(100);"Proteobacteria"(100);Gammaproteobacteria(100);Xanthomonadales(100);Xanthomonadaceae(100);Pseudoxanthomonas(100);
255	1	1	0	0	Bacteria(100);Firmicutes(100);Clostridia(100);Clostridiales(100);Lachnospiraceae(100);unclassified(100);
256	1	1	0	0	Bacteria(100);"Spirochaetes"(100);Spirochaetes(100);Spirochaetales(100);Spirochaetaceae(100);Treponema(100);
257	1	1	0	0	Bacteria(100);Firmicutes(100);Bacilli(100);Bacillales(100);Staphylococcaceae(100);unclassified(100);
258	1	1	0	0	Bacteria(100);"Actinobacteria"(100);Actinobacteria(100);Actinomycetales(100);Nocardiaceae(100);Rhodococcus(86);
259	3	3	0	0	Bacteria(100);"Fusobacteria"(100);"Fusobacteria"(100);"Fusobacteriales"(100);"Leptotrichiaceae"(100);Leptotrichia(72);
260	2	2	0	0	Bacteria(100);"Proteobacteria"(100);Betaproteobacteria(100);Burkholderiales(100);Comamonadaceae(100);unclassified(100);
261	2	2	0	0	Bacteria(100);"Actinobacteria"(100);Actinobacteria(100);Actinomycetales(100);Brevibacteriaceae(100);Brevibacterium(100);
262	1	1	0	0	Bacteria(100);"Actinobacteria"(100);Actinobacteria(100);Actinomycetales(100);Corynebacteriaceae(67);unclassified;
263	1	1	0	0	Bacteria(100);Firmicutes(100);Clostridia(100);Clostridiales(100);Clostridiales_Incertae_Sedis_XIII(100);Mogibacterium(100);
264	1	1	0	0	Bacteria(100);Firmicutes(100);Clostridia(100);Clostridiales(100);Clostridiaceae_1(100);unclassified(100);
265	1	1	0	0	Bacteria(100);unclassified(100);unclassified(100);unclassified(100);unclassified(100);unclassified(100);
266	2	2	0	0	Bacteria(100);"Actinobacteria"(100);Actinobacteria(100);unclassified(100);unclassified(100);unclassified(100);
267	3	3	0	0	Bacteria(100);"Proteobacteria"(100);Betaproteobacteria(100);Burkholderiales(100);Burkholderiaceae(100);Chitinimonas(100);
268	1	1	0	0	Bacteria(100);unclassified(100);unclassified(100);unclassified(100);unclassified(100);unclassified(100);
269	2	2	0	0	Bacteria(100);"Proteobacteria"(100);Alphaproteobacteria(100);Sphingomonadales(100);Sphingomonadaceae(100);Sphingomonas(100);
270	1	1	0	0	Bacteria(100);"Actinobacteria"(100);Actinobacteria(100);Actinomycetales(100);Propionibacteriaceae(100);Propionibacterium(100);
271	1	1	0	0	Bacteria(100);Firmicutes(100);Clostridia(100);Clostridiales(100);Lachnospiraceae(100);unclassified(100);
272	2	2	0	0	Bacteria(100);"Proteobacteria"(100);Gammaproteobacteria(100);Pseudomonadales(100);Moraxellaceae(100);Psychrobacter(100);
273	2	0	2	0	Bacteria(100);"Proteobacteria"(100);Alphaproteobacteria(100);Rhizobiales(100);Bradyrhizobiaceae(100);Bradyrhizobium(67);
274	2	2	0	0	Bacteria(100);"Proteobacteria"(100);Alphaproteobacteria(100);Caulobacterales(100);Caulobacteraceae(100);unclassified(100);
275	1	1	0	0	Bacteria(100);"Proteobacteria"(100);Alphaproteobacteria(100);Rhodobacterales(100);Rhodobacteraceae(100);unclassified(100);

276	2	2	0	0	Bacteria(100);"Proteobacteria"(100);Alphaproteobacteria(100);Rhizobiales(100);Methylobacteriaceae(100);Methylobacterium(100);
277	4	4	0	0	Bacteria(100);"Bacteroidetes"(100);"Bacteroidia"(100);"Bacteroidales"(100);"Prevotellaceae"(100);Prevotella(100);
278	2	2	0	0	Bacteria(100);"Actinobacteria"(100);Actinobacteria(100);Actinomycetales(100);Microbacteriaceae(100);Frondihabitans(100);
279	3	3	0	0	Bacteria(100);"Bacteroidetes"(100);"Bacteroidia"(100);"Bacteroidales"(100);"Prevotellaceae"(100);Prevotella(80);
280	1	1	0	0	Bacteria(100);"Bacteroidetes"(100);unclassified(58);unclassified(58);unclassified(58);unclassified(58);
281	1	1	0	0	Bacteria(100);"Actinobacteria"(100);Actinobacteria(100);Actinomycetales(100);Nocardioidaceae(100);Nocardioides(100);
282	2	2	0	0	Bacteria(100);"Acidobacteria"(100);Acidobacteria_Gp6(100);Acidobacteria_Gp6_order_incertae_sedis(100);Acidobacteria_Gp6_family_incertae_sedis(100);Gp6(100);
283	1	1	0	0	Bacteria(100);Firmicutes(100);Clostridia(100);Clostridiales(100);Lachnospiraceae(100);Catonella(100);
284	2	2	0	0	Bacteria(100);"Bacteroidetes"(100);Flavobacteria(100);"Flavobacteriales"(100);Flavobacteriaceae(100);Capnocytophaga(100);
285	3	3	0	0	Bacteria(100);Firmicutes(100);Bacilli(100);Bacillales(100);Paenibacillaceae_1(100);Paenibacillus(67);
286	1	1	0	0	Bacteria(100);"Bacteroidetes"(100);Flavobacteria(100);"Flavobacteriales"(100);Flavobacteriaceae(100);Flavobacterium(100);
287	2	2	0	0	Bacteria(100);unclassified(100);unclassified(100);unclassified(100);unclassified(100);unclassified(100);
288	1	1	0	0	Bacteria(100);"Bacteroidetes"(100);"Bacteroidia"(100);"Bacteroidales"(100);Bacteroidaceae(100);Bacteroides(100);
289	1	0	0	1	Bacteria(100);"Actinobacteria"(100);Actinobacteria(100);Actinomycetales(100);Propionibacteriaceae(100);unclassified(100);
290	2	1	1	0	Bacteria(100);unclassified(100);unclassified(100);unclassified(100);unclassified(100);unclassified(100);
291	1	1	0	0	Bacteria(100);"Proteobacteria"(100);Alphaproteobacteria(100);unclassified(100);unclassified(100);unclassified(100);
292	4	4	0	0	Bacteria(100);"Actinobacteria"(100);Actinobacteria(100);Actinomycetales(100);unclassified(100);unclassified(100);
293	2	2	0	0	Bacteria(100);"Proteobacteria"(100);Betaproteobacteria(100);Neisseriales(75);Neisseriaceae(75);unclassified(75);
294	5	5	0	0	Bacteria(100);"Actinobacteria"(100);Actinobacteria(100);Actinomycetales(100);Intrasporangiaceae(100);Phycicoccus(100);
295	6	3	0	3	Bacteria(100);Firmicutes(100);Clostridia(100);Clostridiales(100);Clostridiales_Incertae_Sedis_XI(100);Anaerococcus(100);
296	1	1	0	0	Bacteria(100);unclassified(100);unclassified(100);unclassified(100);unclassified(100);unclassified(100);
297	1	1	0	0	Bacteria(100);Firmicutes(100);Erysipelotrichia(100);Erysipelotrichales(100);Erysipelotrichaceae(100);Solobacterium(100);
298	2	2	0	0	Bacteria(100);"Proteobacteria"(100);Alphaproteobacteria(100);Sphingomonadales(100);Sphingomonadaceae(100);Sphingopyxis(100);
299	2	2	0	0	Bacteria(100);"Proteobacteria"(100);Betaproteobacteria(100);Burkholderiales(100);Oxalobacteraceae(100);unclassified(58);
300	1	1	0	0	Bacteria(100);"Actinobacteria"(100);Actinobacteria(100);Actinomycetales(100);Corynebacteriaceae(100);Corynebacterium(100);
301	2	2	0	0	Bacteria(100);"Bacteroidetes"(100);"Bacteroidia"(100);"Bacteroidales"(100);"Prevotellaceae"(100);Prevotella(100);

302	1	1	0	0	Bacteria(100);OD1(100);OD1_class_incertae_sedis(100);OD1_order_incertae_sedis(100);OD1_family_incertae_sedis(100);OD1_genus_incertae_sedis(100);
303	1	1	0	0	Bacteria(100);Firmicutes(100);Bacilli(100);Lactobacillales(100);Streptococcaceae(100);Streptococcus(100);
304	1	1	0	0	Bacteria(100);Firmicutes(100);Bacilli(100);Bacillales(100);unclassified(100);unclassified(100);
305	1	1	0	0	Bacteria(100);Firmicutes(100);Clostridia(100);Clostridiales(100);Lachnospiraceae(100);Blautia(100);
306	2	2	0	0	Bacteria(100);unclassified(100);unclassified(100);unclassified(100);unclassified(100);unclassified(100);
307	1	1	0	0	Bacteria(100);"Actinobacteria"(100);Actinobacteria(100);Actinomycetales(100);unclassified(67);unclassified(67);
308	1	1	0	0	Bacteria(100);"Proteobacteria"(100);Alphaproteobacteria(100);Sphingomonadales(100);Sphingomonadaceae(100);unclassified;
309	2	2	0	0	Bacteria(100);"Chloroflexi"(100);Thermomicrobia(100);Sphaerobacterales(67);Sphaerobacteraceae(67);Sphaerobacter(67);
310	1	1	0	0	Bacteria(100);"Proteobacteria"(100);Gammaproteobacteria(100);Pasteurellales(100);Pasteurellaceae(100);unclassified(100);
311	2	2	0	0	Bacteria(100);"Proteobacteria"(100);Deltaproteobacteria(100);unclassified(100);unclassified(100);unclassified(100);
312	1	1	0	0	Bacteria(100);"Proteobacteria"(100);Alphaproteobacteria(100);Rhizobiales(100);unclassified;unclassified;
313	2	2	0	0	Bacteria(100);"Actinobacteria"(100);Actinobacteria(100);Actinomycetales(100);unclassified(100);unclassified(100);
314	1	1	0	0	Bacteria(100);"Proteobacteria"(100);Alphaproteobacteria(100);Caulobacterales(100);Caulobacteraceae(100);Caulobacter(100);
315	1	1	0	0	Bacteria(100);Firmicutes(100);Negativicutes(100);Selenomonadales(100);Veillonellaceae(100);Dialister(100);
316	2	2	0	0	Bacteria(100);unclassified(100);unclassified(100);unclassified(100);unclassified(100);unclassified(100);
317	1	1	0	0	Bacteria(100);"Bacteroidetes"(100);"Bacteroidia"(100);"Bacteroidales"(100);"Porphyromonadaceae"(100);Porphyromonas(100);
318	2	2	0	0	Bacteria(100);"Proteobacteria"(100);Alphaproteobacteria(100);Rhizobiales(100);Brucellaceae(100);Ochrobactrum(100);
319	2	2	0	0	Bacteria(100);"Actinobacteria"(100);Actinobacteria(100);Coriobacteriales(100);Coriobacteriaceae(100);Olsenella(100);
320	3	3	0	0	Bacteria(100);"Bacteroidetes"(100);Flavobacteria(100);"Flavobacteriales"(100);Flavobacteriaceae(100);unclassified(100);
321	1	1	0	0	Bacteria(100);"Proteobacteria"(100);Gammaproteobacteria(100);Pseudomonadales(100);Moraxellaceae(100);Acinetobacter(100);
322	2	2	0	0	Bacteria(100);Firmicutes(100);Clostridia(100);Clostridiales(100);Lachnospiraceae(100);Blautia(100);
323	2	2	0	0	Bacteria(100);Firmicutes(100);Bacilli(100);Lactobacillales(100);Carnobacteriaceae(100);unclassified(67);
324	1	1	0	0	Bacteria(100);"Verrucomicrobia"(100);Verrucomicrobiae(100);Verrucomicrobiales(100);Verrucomicrobiaceae(100);unclassified;
325	1	1	0	0	Bacteria(100);Firmicutes(100);Clostridia(100);Clostridiales(100);Ruminococcaceae(100);unclassified(100);
326	3	2	1	0	Bacteria(100);"Bacteroidetes"(100);"Bacteroidia"(100);"Bacteroidales"(100);"Prevotellaceae"(100);Prevotella(100);
327	3	2	1	0	Bacteria(100);Firmicutes(100);Clostridia(100);Clostridiales(100);unclassified(100);unclassified(100);
328	4	4	0	0	Bacteria(100);"Proteobacteria"(100);Betaproteobacteria(100);Burkholderiales(100);Oxalobacteraceae(100);unclassified(100);

329	1	1	0	0	Bacteria(100);"Proteobacteria"(100);Betaproteobacteria(100);Burkholderiales(100);Oxalobacteraceae(100);unclassified;
330	1	1	0	0	Bacteria(100);"Proteobacteria"(100);Alphaproteobacteria(100);Sphingomonadales(100);Sphingomonadaceae(100);Novosphingobium(100);
331	1	1	0	0	Bacteria(100);"Proteobacteria"(100);Betaproteobacteria(100);unclassified(100);unclassified(100);unclassified(100);
332	3	3	0	0	Bacteria(100);unclassified(60);unclassified(60);unclassified(60);unclassified(60);unclassified(60);
333	1	1	0	0	Bacteria(100);"Bacteroidetes"(100);unclassified(100);unclassified(100);unclassified(100);unclassified(100);
334	1	1	0	0	Bacteria(100);"Proteobacteria"(100);Gammaproteobacteria(100);Pseudomonadales(100);Moraxellaceae(100);Alkanindiges(100);
335	2	2	0	0	Bacteria(100);"Bacteroidetes"(100);"Bacteroidia"(100);"Bacteroidales"(100);"Prevotellaceae"(100);Prevotella(100);
336	1	1	0	0	Bacteria(100);"Bacteroidetes"(100);"Sphingobacteria"(100);"Sphingobacteriales"(100);Cytophagaceae(100);Arcicella(100);
337	1	1	0	0	Bacteria(100);"Actinobacteria"(100);Actinobacteria(100);Actinomycetales(100);unclassified(100);unclassified(100);
338	1	1	0	0	Bacteria(100);"Actinobacteria"(100);Actinobacteria(100);Actinomycetales(100);unclassified;unclassified;
339	2	2	0	0	Bacteria(100);unclassified(100);unclassified(100);unclassified(100);unclassified(100);unclassified(100);
340	4	4	0	0	Bacteria(100);"Proteobacteria"(100);unclassified(100);unclassified(100);unclassified(100);unclassified(100);
341	1	1	0	0	Bacteria(100);"Proteobacteria"(100);unclassified(100);unclassified(100);unclassified(100);unclassified(100);
342	1	1	0	0	Bacteria(100);unclassified(100);unclassified(100);unclassified(100);unclassified(100);unclassified(100);
343	1	1	0	0	Bacteria(100);Firmicutes(100);Bacilli(100);unclassified(100);unclassified(100);unclassified(100);
344	1	1	0	0	Bacteria(100);"Proteobacteria"(100);Deltaproteobacteria(100);Bdellovibrionales(100);Bacteriovoracaceae(100);Peredibacter(100);
345	2	2	0	0	Bacteria(100);Firmicutes(100);Bacilli(100);Lactobacillales(100);Streptococcaceae(100);Streptococcus(100);
346	1	1	0	0	Bacteria(100);Firmicutes(100);Clostridia(100);Clostridiales(100);Lachnospiraceae(100);unclassified(100);
347	3	3	0	0	Bacteria(100);"Actinobacteria"(100);Actinobacteria(100);Actinomycetales(100);Brevibacteriaceae(100);Brevibacterium(100);
348	1	1	0	0	Bacteria(100);"Bacteroidetes"(100);unclassified(100);unclassified(100);unclassified(100);unclassified(100);
349	1	1	0	0	Bacteria(100);"Deinococcus-Thermus"(100);Deinococci(100);Deinococcales(100);Deinococcaceae(100);Deinococcus(100);
350	2	2	0	0	Bacteria(100);"Actinobacteria"(100);Actinobacteria(100);Coriobacteriales(100);Coriobacteriaceae(100);Olsenella(100);
351	2	2	0	0	Bacteria(100);"Proteobacteria"(100);Alphaproteobacteria(100);unclassified(100);unclassified(100);unclassified(100);
352	1	1	0	0	Bacteria(100);unclassified(100);unclassified(100);unclassified(100);unclassified(100);unclassified(100);
353	1	1	0	0	Bacteria(100);Firmicutes(100);Negativicutes(100);Selenomonadales(100);Veillonellaceae(100);Veillonella(100);
354	1	1	0	0	Bacteria(100);"Proteobacteria"(100);Epsilonproteobacteria(100);Campylobacterales(100);Campylobacteraceae(100);Arcobacter(100);
355	3	3	0	0	Bacteria(100);"Proteobacteria"(100);Alphaproteobacteria(100);Rhizobiales(100);Hyphomicrobiaceae(100);unclassified(100);
356	1	1	0	0	Bacteria(100);Firmicutes(100);Bacilli(100);Lactobacillales(100);Carnobacteriaceae(100);Atopostipes(100);
357	1	1	0	0	Bacteria(100);unclassified(100);unclassified(100);unclassified(100);unclassified(100);unclassified(100);

358	1	1	0	0	Bacteria(100);"Actinobacteria"(100);Actinobacteria(100);Actinomycetales(100);unclassified;unclassified;
359	1	1	0	0	Bacteria(100);Firmicutes(100);Clostridia(100);Clostridiales(100);Lachnospiraceae(100);Clostridium_XlVb(100);
360	1	1	0	0	Bacteria(100);unclassified(67);unclassified(67);unclassified(67);unclassified(67);unclassified(67);
361	1	1	0	0	Bacteria(100);unclassified(100);unclassified(100);unclassified(100);unclassified(100);unclassified(100);
362	1	1	0	0	Bacteria(100);Firmicutes(100);Bacilli(100);Lactobacillales(100);Lactobacillaceae(100);Lactobacillus(100);
363	1	1	0	0	Bacteria(100);"Proteobacteria"(100);Betaproteobacteria(100);Burkholderiales(100);unclassified(100);unclassified(100);
364	1	1	0	0	Bacteria(100);"Gemmatimonadetes"(100);Gemmatimonadetes(100);Gemmatimonadales(100);Gemmatimonadaceae(100);Gemmatimonas(100);
365	1	1	0	0	Bacteria(100);"Proteobacteria"(100);Betaproteobacteria(100);Burkholderiales(100);Oxalobacteraceae(100);unclassified(100);
366	1	1	0	0	Bacteria(100);"Actinobacteria"(100);Actinobacteria(100);Actinomycetales(100);Propionibacteriaceae(100);Propioniferax(100);
367	1	1	0	0	Bacteria(100);unclassified(100);unclassified(100);unclassified(100);unclassified(100);unclassified(100);
368	1	1	0	0	Bacteria(100);unclassified(100);unclassified(100);unclassified(100);unclassified(100);unclassified(100);
369	1	1	0	0	Bacteria(100);"Proteobacteria"(100);Alphaproteobacteria(100);unclassified(100);unclassified(100);unclassified(100);
370	1	1	0	0	Bacteria(100);"Proteobacteria"(100);Gammaproteobacteria(100);"Enterobacteriales"(100);Enterobacteriaceae(100);unclassified(100);
371	1	1	0	0	Bacteria(100);unclassified(100);unclassified(100);unclassified(100);unclassified(100);unclassified(100);
372	1	1	0	0	Bacteria(100);"Bacteroidetes"(100);"Bacteroidia"(100);"Bacteroidales"(100);"Prevotellaceae"(100);Prevotella(100);
373	1	1	0	0	Bacteria(100);Firmicutes(100);Bacilli(100);Lactobacillales(100);unclassified(100);unclassified(100);
374	1	1	0	0	Bacteria(100);unclassified(100);unclassified(100);unclassified(100);unclassified(100);unclassified(100);
375	1	1	0	0	Bacteria(100);unclassified(100);unclassified(100);unclassified(100);unclassified(100);unclassified(100);
376	1	1	0	0	Bacteria(100);Firmicutes(100);Bacilli(100);Lactobacillales(100);unclassified(100);unclassified(100);
377	1	1	0	0	Bacteria(100);"Bacteroidetes"(100);"Bacteroidia"(100);"Bacteroidales"(100);unclassified(100);unclassified(100);
378	1	1	0	0	Bacteria(100);unclassified(100);unclassified(100);unclassified(100);unclassified(100);unclassified(100);
379	1	1	0	0	Bacteria(100);unclassified(100);unclassified(100);unclassified(100);unclassified(100);unclassified(100);
380	1	1	0	0	Bacteria(100);"Proteobacteria"(100);Gammaproteobacteria(100);Pasteurellales(100);Pasteurellaceae(100);Mannheimia(100);
381	1	1	0	0	Bacteria(100);"Fusobacteria"(100);"Fusobacteria"(100);"Fusobacteriales"(100);"Fusobacteriaceae"(100);Fusobacterium(100);
382	1	1	0	0	Bacteria(100);unclassified(100);unclassified(100);unclassified(100);unclassified(100);unclassified(100);
383	1	1	0	0	Bacteria(100);TM7(100);TM7_class_incertae_sedis(100);TM7_order_incertae_sedis(100);TM7_family_incertae_sedis(100);TM7_genus_incertae_sedis(100);
384	1	1	0	0	Bacteria(100);Firmicutes(100);Bacilli(100);Lactobacillales(100);Streptococcaceae(100);Streptococcus(100);
385	1	1	0	0	Bacteria(100);Firmicutes(100);Negativicutes(100);Selenomonadales(100);Veillonellaceae(100);unclassified(100);
386	1	1	0	0	Bacteria(100);Firmicutes(100);Bacilli(100);Lactobacillales(100);Streptococcaceae(100);Streptococcus(100);

387	1	1	0	0	Bacteria(100);"Actinobacteria"(100);Actinobacteria(100);Actinomycetales(100);Microbacteriaceae(100);unclassified(100);
388	1	1	0	0	Bacteria(100);"Proteobacteria"(100);Betaproteobacteria(100);Neisseriales(100);Neisseriaceae(100);Neisseria(100);
389	1	1	0	0	Bacteria(100);"Proteobacteria"(100);Betaproteobacteria(100);Neisseriales(100);Neisseriaceae(100);unclassified(100);
390	1	1	0	0	Bacteria(100);"Bacteroidetes"(100);Flavobacteria(100);"Flavobacteriales"(100);Flavobacteriaceae(100);Capnocytophaga(100);
391	1	1	0	0	Bacteria(100);Firmicutes(100);Clostridia(100);Clostridiales(100);Clostridiaceae_1(100);unclassified(100);
392	1	1	0	0	Bacteria(100);"Proteobacteria"(100);Alphaproteobacteria(100);Sphingomonadales(100);unclassified(100);unclassified(100);
393	1	1	0	0	Bacteria(100);"Proteobacteria"(100);Betaproteobacteria(100);Burkholderiales(100);unclassified(100);unclassified(100);
394	1	1	0	0	Bacteria(100);"Bacteroidetes"(100);Flavobacteria(100);"Flavobacteriales"(100);Flavobacteriaceae(100);unclassified(100);
395	1	1	0	0	Bacteria(100);unclassified(100);unclassified(100);unclassified(100);unclassified(100);unclassified(100);
396	1	1	0	0	Bacteria(100);Firmicutes(100);Clostridia(100);Clostridiales(100);Clostridiales_Incertae_Sedis_XI(100);Anaerococcus(100);
397	1	1	0	0	Bacteria(100);Firmicutes(100);Clostridia(100);Clostridiales(100);Lachnospiraceae(100);unclassified(100);
398	1	1	0	0	Bacteria(100);"Proteobacteria"(100);Betaproteobacteria(100);Neisseriales(100);Neisseriaceae(100);unclassified(100);
399	1	1	0	0	Bacteria(100);"Bacteroidetes"(100);Flavobacteria(100);"Flavobacteriales"(100);Flavobacteriaceae(100);Capnocytophaga(100);
400	1	1	0	0	Bacteria(100);"Actinobacteria"(100);Actinobacteria(100);Actinomycetales(100);Nocardiaceae(100);Rhodococcus(100);
401	1	1	0	0	Bacteria(100);unclassified(100);unclassified(100);unclassified(100);unclassified(100);unclassified(100);
402	1	1	0	0	Bacteria(100);Firmicutes(100);Negativicutes(100);Selenomonadales(100);Veillonellaceae(100);Veillonella(100);
403	1	1	0	0	Bacteria(100);"Bacteroidetes"(100);"Sphingobacteria"(100);"Sphingobacteriales"(100);Chitinophagaceae(100);Hydrotalea(100);
404	1	1	0	0	Bacteria(100);"Fusobacteria"(100);"Fusobacteria"(100);"Fusobacteriales"(100);"Fusobacteriaceae"(100);unclassified(100);
405	1	1	0	0	Bacteria(100);"Proteobacteria"(100);Alphaproteobacteria(100);Rhizobiales(100);Methylobacteriaceae(100);Methylobacterium(100);
406	1	1	0	0	Bacteria(100);"Proteobacteria"(100);Gammaproteobacteria(100);"Enterobacteriales"(100);Enterobacteriaceae(100);unclassified(100);
407	1	1	0	0	Bacteria(100);"Proteobacteria"(100);Gammaproteobacteria(100);unclassified(100);unclassified(100);unclassified(100);
408	1	1	0	0	Bacteria(100);unclassified(100);unclassified(100);unclassified(100);unclassified(100);unclassified(100);
409	1	1	0	0	Bacteria(100);unclassified(100);unclassified(100);unclassified(100);unclassified(100);unclassified(100);
410	1	1	0	0	Bacteria(100);"Proteobacteria"(100);Gammaproteobacteria(100);"Enterobacteriales"(100);Enterobacteriaceae(100);unclassified(100);
411	1	1	0	0	Bacteria(100);Firmicutes(100);Bacilli(100);Bacillales(100);unclassified(100);unclassified(100);
412	1	1	0	0	Bacteria(100);Firmicutes(100);Bacilli(100);unclassified(100);unclassified(100);unclassified(100);

413	1	1	0	0	Bacteria(100);"Actinobacteria"(100);Actinobacteria(100);Actinomycetales(100);Actinomycetaceae(100);Actinomyces(100);
414	1	1	0	0	Bacteria(100);Firmicutes(100);Bacilli(100);Lactobacillales(100);Streptococcaceae(100);Streptococcus(100);
415	1	1	0	0	Bacteria(100);Firmicutes(100);Bacilli(100);Lactobacillales(100);Streptococcaceae(100);Streptococcus(100);
416	1	1	0	0	Bacteria(100);"Proteobacteria"(100);Betaproteobacteria(100);Neisseriales(100);Neisseriaceae(100);Neisseria(100);
417	1	1	0	0	Bacteria(100);"Proteobacteria"(100);unclassified(100);unclassified(100);unclassified(100);unclassified(100);
418	1	1	0	0	Bacteria(100);Firmicutes(100);Clostridia(100);Clostridiales(100);Lachnospiraceae(100);Anaerostipes(100);
419	1	1	0	0	Bacteria(100);"Proteobacteria"(100);Gammaproteobacteria(100);"Enterobacteriales"(100);Enterobacteriaceae(100);Escherichia_Shigella(100);
420	1	1	0	0	Bacteria(100);TM7(100);TM7_class_incertae_sedis(100);TM7_order_incertae_sedis(100);TM7_family_incertae_sedis(100);TM7_genus_incertae_sedis(100);
421	1	1	0	0	Bacteria(100);unclassified(100);unclassified(100);unclassified(100);unclassified(100);unclassified(100);
422	1	1	0	0	Bacteria(100);"Fusobacteria"(100);"Fusobacteria"(100);"Fusobacteriales"(100);"Leptotrichiaceae"(100);Leptotrichia(100);
423	1	0	1	0	Bacteria(100);unclassified(100);unclassified(100);unclassified(100);unclassified(100);unclassified(100);
424	1	1	0	0	Bacteria(100);"Proteobacteria"(100);Gammaproteobacteria(100);Xanthomonadales(100);Xanthomonadaceae(100);Pseudoxanthomonas(100);
425	1	1	0	0	Bacteria(100);unclassified(100);unclassified(100);unclassified(100);unclassified(100);unclassified(100);
426	1	1	0	0	Bacteria(100);"Proteobacteria"(100);Deltaproteobacteria(100);Myxococcales(100);Polyangiaceae(100);Chondromyces(100);
427	2	2	0	0	Bacteria(100);"Actinobacteria"(100);Actinobacteria(100);Actinomycetales(100);Nocardioidaceae(100);Marmoricola(100);
428	1	1	0	0	Bacteria(100);unclassified(100);unclassified(100);unclassified(100);unclassified(100);unclassified(100);
429	1	1	0	0	Bacteria(100);unclassified(100);unclassified(100);unclassified(100);unclassified(100);unclassified(100);
430	1	1	0	0	Bacteria(100);unclassified(100);unclassified(100);unclassified(100);unclassified(100);unclassified(100);
431	1	1	0	0	Bacteria(100);unclassified(100);unclassified(100);unclassified(100);unclassified(100);unclassified(100);
432	1	1	0	0	Bacteria(100);Firmicutes(100);Bacilli(100);Lactobacillales(100);Carnobacteriaceae(100);unclassified(100);
433	1	1	0	0	Bacteria(100);unclassified(100);unclassified(100);unclassified(100);unclassified(100);unclassified(100);
434	2	2	0	0	Bacteria(100);"Verrucomicrobia"(100);Subdivision5(100);Subdivision5_order_incertae_sedis(100);Subdivision5_family_incertae_sedis(100);5_genus_incertae_sedis(100);
435	1	1	0	0	Bacteria(100);"Actinobacteria"(100);Actinobacteria(100);Actinomycetales(100);unclassified(100);unclassified(100);
436	1	1	0	0	Bacteria(100);"Proteobacteria"(100);Epsilonproteobacteria(100);Campylobacterales(100);Helicobacteraceae(100);Wolinella(100);
437	1	1	0	0	Bacteria(100);Firmicutes(100);Clostridia(100);Clostridiales(100);Clostridiales_Incertae_Sedis_XI(100);Anaerococcus(100);
438	1	1	0	0	Bacteria(100);unclassified(100);unclassified(100);unclassified(100);unclassified(100);unclassified(100);
439	1	1	0	0	Bacteria(100);unclassified(100);unclassified(100);unclassified(100);unclassified(100);unclassified(100);

440	1	1	0	0	Bacteria(100);"Fusobacteria"(100);"Fusobacteria"(100);"Fusobacteriales"(100);"Fusobacteriaceae"(100);Cetobacterium(100);
441	1	1	0	0	Bacteria(100);"Fusobacteria"(100);"Fusobacteria"(100);"Fusobacteriales"(100);"Fusobacteriaceae"(100);Fusobacterium(100);
442	1	1	0	0	Bacteria(100);Firmicutes(100);Clostridia(100);Clostridiales(100);Clostridiaceae_1(100);unclassified(100);
443	1	1	0	0	Bacteria(100);"Proteobacteria"(100);unclassified(100);unclassified(100);unclassified(100);unclassified(100);
444	1	1	0	0	Bacteria(100);unclassified(100);unclassified(100);unclassified(100);unclassified(100);unclassified(100);
445	1	1	0	0	Bacteria(100);"Bacteroidetes"(100);"Sphingobacteria"(100);"Sphingobacteriales"(100);Cytophagaceae(100);Hymenobacter(100);
446	1	1	0	0	Bacteria(100);unclassified(100);unclassified(100);unclassified(100);unclassified(100);unclassified(100);
447	2	2	0	0	Bacteria(100);unclassified(100);unclassified(100);unclassified(100);unclassified(100);unclassified(100);
448	1	1	0	0	Bacteria(100);"Proteobacteria"(100);Alphaproteobacteria(100);Sphingomonadales(100);Sphingomonadaceae(100);Sphingobium(100);
449	1	1	0	0	Bacteria(100);"Proteobacteria"(100);Betaproteobacteria(100);Burkholderiales(100);Sutterellaceae(100);Sutterella(100);
450	1	1	0	0	Bacteria(100);"Actinobacteria"(100);Actinobacteria(100);Actinomycetales(100);unclassified(100);unclassified(100);
451	1	1	0	0	Bacteria(100);Firmicutes(100);Bacilli(100);Lactobacillales(100);Lactobacillaceae(100);Lactobacillus(100);
452	1	1	0	0	Bacteria(100);unclassified(100);unclassified(100);unclassified(100);unclassified(100);unclassified(100);
453	1	1	0	0	Bacteria(100);"Proteobacteria"(100);Gammaproteobacteria(100);Chromatiales(100);Halothiobacillaceae(100);Thiofaba(100);
454	1	1	0	0	Bacteria(100);unclassified(100);unclassified(100);unclassified(100);unclassified(100);unclassified(100);
455	1	1	0	0	Bacteria(100);Firmicutes(100);Bacilli(100);Lactobacillales(100);Streptococcaceae(100);Streptococcus(100);
456	1	1	0	0	Bacteria(100);"Fusobacteria"(100);"Fusobacteria"(100);"Fusobacteriales"(100);"Leptotrichiaceae"(100);Leptotrichia(100);
457	1	1	0	0	Bacteria(100);Firmicutes(100);Clostridia(100);Clostridiales(100);unclassified(100);unclassified(100);
458	1	1	0	0	Bacteria(100);Firmicutes(100);Clostridia(100);Clostridiales(100);unclassified(100);unclassified(100);
459	1	1	0	0	Bacteria(100);"Proteobacteria"(100);Alphaproteobacteria(100);Rhizobiales(100);unclassified(100);unclassified(100);
460	1	1	0	0	Bacteria(100);unclassified(100);unclassified(100);unclassified(100);unclassified(100);unclassified(100);
461	1	1	0	0	Bacteria(100);"Proteobacteria"(100);Alphaproteobacteria(100);Rhizobiales(100);"Aurantimonadaceae"(100);Aurantimonas(100);
462	1	1	0	0	Bacteria(100);unclassified(100);unclassified(100);unclassified(100);unclassified(100);unclassified(100);
463	1	1	0	0	Bacteria(100);"Proteobacteria"(100);Alphaproteobacteria(100);unclassified(100);unclassified(100);unclassified(100);
464	1	1	0	0	Bacteria(100);unclassified(100);unclassified(100);unclassified(100);unclassified(100);unclassified(100);
465	1	1	0	0	Bacteria(100);unclassified(100);unclassified(100);unclassified(100);unclassified(100);unclassified(100);
466	1	1	0	0	Bacteria(100);Firmicutes(100);Clostridia(100);Clostridiales(100);Clostridiaceae_1(100);unclassified(100);
467	1	1	0	0	Bacteria(100);unclassified(100);unclassified(100);unclassified(100);unclassified(100);unclassified(100);
468	1	1	0	0	Bacteria(100);Firmicutes(100);Clostridia(100);Clostridiales(100);Lachnospiraceae(100);unclassified(100);
469	1	1	0	0	Bacteria(100);unclassified(100);unclassified(100);unclassified(100);unclassified(100);unclassified(100);

470	1	1	0	0	Bacteria(100);"Proteobacteria"(100);Alphaproteobacteria(100);Sphingomonadales(100);Erythrobacteraceae(100);Erythrobacter(100);
471	2	2	0	0	Bacteria(100);Firmicutes(100);Erysipelotrichia(100);Erysipelotrichales(100);Erysipelotrichaceae(100);Catenibacterium(100);
472	1	1	0	0	Bacteria(100);Firmicutes(100);Negativicutes(100);Selenomonadales(100);Veillonellaceae(100);unclassified(100);
473	1	1	0	0	Bacteria(100);"Proteobacteria"(100);Betaproteobacteria(100);Neisseriales(100);Neisseriaceae(100);unclassified(100);
474	1	1	0	0	Bacteria(100);Firmicutes(100);Bacilli(100);Lactobacillales(100);Carnobacteriaceae(100);unclassified(100);
475	1	1	0	0	Bacteria(100);"Actinobacteria"(100);Actinobacteria(100);Actinomycetales(100);Microbacteriaceae(100);Leucobacter(100);
476	3	3	0	0	Bacteria(100);TM7(100);TM7_class_incertae_sedis(100);TM7_order_incertae_sedis(100);TM7_family_incertae_sedis(100);TM7_genus_incertae_sedis(100);
477	1	1	0	0	Bacteria(100);unclassified(100);unclassified(100);unclassified(100);unclassified(100);unclassified(100);
478	1	1	0	0	Bacteria(100);unclassified(100);unclassified(100);unclassified(100);unclassified(100);unclassified(100);
479	1	1	0	0	Bacteria(100);Firmicutes(100);Bacilli(100);Lactobacillales(100);Streptococcaceae(100);Streptococcus(100);
480	1	0	0	1	Bacteria(100);"Actinobacteria"(100);Actinobacteria(100);Actinomycetales(100);Nocardioidaceae(100);Nocardioides(100);
481	1	1	0	0	Bacteria(100);"Proteobacteria"(100);Betaproteobacteria(100);Neisseriales(100);Neisseriaceae(100);Neisseria(100);
482	1	1	0	0	Bacteria(100);unclassified(100);unclassified(100);unclassified(100);unclassified(100);unclassified(100);
483	1	1	0	0	Bacteria(100);"Actinobacteria"(100);Actinobacteria(100);Actinomycetales(100);unclassified(100);unclassified(100);
484	3	3	0	0	Bacteria(100);unclassified(100);unclassified(100);unclassified(100);unclassified(100);unclassified(100);
485	1	1	0	0	Bacteria(100);Firmicutes(100);Bacilli(100);Bacillales(100);Staphylococcaceae(100);Staphylococcus(100);
486	1	1	0	0	Bacteria(100);Firmicutes(100);Bacilli(100);Lactobacillales(100);unclassified(100);unclassified(100);
487	1	1	0	0	Bacteria(100);"Proteobacteria"(100);Alphaproteobacteria(100);Sphingomonadales(100);unclassified(100);unclassified(100);
488	1	1	0	0	Bacteria(100);"Actinobacteria"(100);Actinobacteria(100);Actinomycetales(100);unclassified(100);unclassified(100);
489	1	1	0	0	Bacteria(100);"Proteobacteria"(100);Alphaproteobacteria(100);Sphingomonadales(100);Erythrobacteraceae(100);unclassified(100);
490	1	1	0	0	Bacteria(100);Firmicutes(100);Clostridia(100);Clostridiales(100);Clostridiaceae_1(100);unclassified(100);
491	1	1	0	0	Bacteria(100);"Actinobacteria"(100);Actinobacteria(100);Coriobacteriales(100);Coriobacteriaceae(100);Atopobium(100);
492	1	1	0	0	Bacteria(100);"Proteobacteria"(100);Alphaproteobacteria(100);Rhizobiales(100);unclassified(100);unclassified(100);
493	1	1	0	0	Bacteria(100);"Bacteroidetes"(100);Flavobacteria(100);"Flavobacteriales"(100);Cryomorphaceae(100);Fluviicola(100);
494	1	1	0	0	Bacteria(100);"Proteobacteria"(100);Betaproteobacteria(100);Rhodocyclales(100);Rhodocyclaceae(100);unclassified(100);
495	1	1	0	0	Bacteria(100);unclassified(100);unclassified(100);unclassified(100);unclassified(100);unclassified(100);
496	2	2	0	0	Bacteria(100);"Proteobacteria"(100);Betaproteobacteria(100);Burkholderiales(100);unclassified(100);unclassified(100);
497	2	2	0	0	Bacteria(100);"Actinobacteria"(100);Actinobacteria(100);Coriobacteriales(100);Coriobacteriaceae(100);Olsenella(100);

498	1	1	0	0	Bacteria(100);"Fusobacteria"(100);"Fusobacteria"(100);"Fusobacteriales"(100);"Fusobacteriaceae"(100);Cetobacterium(100);
499	1	1	0	0	Bacteria(100);Firmicutes(100);Negativicutes(100);Selenomonadales(100);Veillonellaceae(100);unclassified(100);
500	1	1	0	0	Bacteria(100);"Actinobacteria"(100);Actinobacteria(100);Actinomycetales(100);unclassified(100);unclassified(100);
501	1	1	0	0	Bacteria(100);unclassified(100);unclassified(100);unclassified(100);unclassified(100);unclassified(100);
502	1	1	0	0	Bacteria(100);unclassified(100);unclassified(100);unclassified(100);unclassified(100);unclassified(100);
503	1	1	0	0	Bacteria(100);unclassified(100);unclassified(100);unclassified(100);unclassified(100);unclassified(100);
504	1	1	0	0	Bacteria(100);OD1(100);OD1_class_incertae_sedis(100);OD1_order_incertae_sedis(100);OD1_family_incertae_sedis(100);OD1_genus_incertae_sedis(100);
505	1	1	0	0	Bacteria(100);Firmicutes(100);Clostridia(100);Clostridiales(100);Clostridiaceae_1(100);unclassified(100);
506	1	1	0	0	Bacteria(100);unclassified(100);unclassified(100);unclassified(100);unclassified(100);unclassified(100);
507	1	1	0	0	Bacteria(100);"Proteobacteria"(100);Gammaproteobacteria(100);unclassified(100);unclassified(100);unclassified(100);
508	1	1	0	0	Bacteria(100);Firmicutes(100);Bacilli(100);Lactobacillales(100);Streptococcaceae(100);Streptococcus(100);
509	1	1	0	0	Bacteria(100);"Proteobacteria"(100);Gammaproteobacteria(100);Pasteurellales(100);Pasteurellaceae(100);unclassified(100);
510	1	1	0	0	Bacteria(100);"Actinobacteria"(100);Actinobacteria(100);Actinomycetales(100);Nocardioidaceae(100);Nocardioides(100);
511	1	1	0	0	Bacteria(100);unclassified(100);unclassified(100);unclassified(100);unclassified(100);unclassified(100);
512	3	3	0	0	Bacteria(100);"Proteobacteria"(100);Alphaproteobacteria(100);Rhizobiales(100);Rhizobiaceae(100);Rhizobium(100);
513	1	1	0	0	Bacteria(100);Firmicutes(100);Bacilli(100);Bacillales(100);Staphylococcaceae(100);Staphylococcus(100);
514	1	1	0	0	Bacteria(100);"Proteobacteria"(100);Gammaproteobacteria(100);Xanthomonadales(100);Xanthomonadaceae(100);unclassified(100);
515	1	1	0	0	Bacteria(100);"Proteobacteria"(100);Gammaproteobacteria(100);Pseudomonadales(100);Pseudomonadaceae(100);unclassified(100);
516	1	1	0	0	Bacteria(100);"Fusobacteria"(100);"Fusobacteria"(100);"Fusobacteriales"(100);"Fusobacteriaceae"(100);unclassified(100);
517	1	1	0	0	Bacteria(100);Firmicutes(100);Bacilli(100);Lactobacillales(100);unclassified(100);unclassified(100);
518	1	1	0	0	Bacteria(100);unclassified(100);unclassified(100);unclassified(100);unclassified(100);unclassified(100);
519	1	1	0	0	Bacteria(100);unclassified(100);unclassified(100);unclassified(100);unclassified(100);unclassified(100);
520	1	1	0	0	Bacteria(100);unclassified(100);unclassified(100);unclassified(100);unclassified(100);unclassified(100);
521	1	1	0	0	Bacteria(100);"Proteobacteria"(100);Alphaproteobacteria(100);unclassified(100);unclassified(100);unclassified(100);
522	1	1	0	0	Bacteria(100);"Actinobacteria"(100);Actinobacteria(100);Actinomycetales(100);unclassified(100);unclassified(100);
523	1	1	0	0	Bacteria(100);"Proteobacteria"(100);Alphaproteobacteria(100);Rhodobacterales(100);Rhodobacteraceae(100);unclassified(100);
524	1	1	0	0	Bacteria(100);"Actinobacteria"(100);Actinobacteria(100);Actinomycetales(100);Actinomycetaceae(100);Actinomyces(100);
525	1	1	0	0	Bacteria(100);Firmicutes(100);Bacilli(100);Lactobacillales(100);unclassified(100);unclassified(100);
526	1	1	0	0	Bacteria(100);Firmicutes(100);Bacilli(100);Lactobacillales(100);unclassified(100);unclassified(100);

527	1	1	0	0	Bacteria(100);unclassified(100);unclassified(100);unclassified(100);unclassified(100);unclassified(100);
528	1	1	0	0	Bacteria(100);"Proteobacteria"(100);Gammaproteobacteria(100);Pseudomonadales(100);Pseudomonadaceae(100);unclassified(100);
529	1	1	0	0	Bacteria(100);"Proteobacteria"(100);Alphaproteobacteria(100);Caulobacterales(100);Caulobacteraceae(100);Brevundimonas(100);
530	1	1	0	0	Bacteria(100);"Proteobacteria"(100);Betaproteobacteria(100);Neisseriales(100);Neisseriaceae(100);Neisseria(100);
531	1	1	0	0	Bacteria(100);"Fusobacteria"(100);"Fusobacteria"(100);"Fusobacteriales"(100);"Fusobacteriaceae"(100);unclassified(100);
532	1	1	0	0	Bacteria(100);unclassified(100);unclassified(100);unclassified(100);unclassified(100);unclassified(100);
533	1	1	0	0	Bacteria(100);"Proteobacteria"(100);Alphaproteobacteria(100);Rhodospirillales(100);Acetobacteraceae(100);Roseomonas(100);
534	1	1	0	0	Bacteria(100);unclassified(100);unclassified(100);unclassified(100);unclassified(100);unclassified(100);
535	1	1	0	0	Bacteria(100);"Actinobacteria"(100);Actinobacteria(100);Actinomycetales(100);Micrococcaceae(100);Rothia(100);
536	1	1	0	0	Bacteria(100);unclassified(100);unclassified(100);unclassified(100);unclassified(100);unclassified(100);
537	1	1	0	0	Bacteria(100);"Actinobacteria"(100);Actinobacteria(100);Acidimicrobiales(100);unclassified(100);unclassified(100);
538	1	1	0	0	Bacteria(100);"Proteobacteria"(100);Alphaproteobacteria(100);unclassified(100);unclassified(100);unclassified(100);
539	1	1	0	0	Bacteria(100);unclassified(100);unclassified(100);unclassified(100);unclassified(100);unclassified(100);
540	1	1	0	0	Bacteria(100);unclassified(100);unclassified(100);unclassified(100);unclassified(100);unclassified(100);
541	1	1	0	0	Bacteria(100);"Proteobacteria"(100);Gammaproteobacteria(100);Pasteurellales(100);Pasteurellaceae(100);unclassified(100);
542	1	1	0	0	Bacteria(100);unclassified(100);unclassified(100);unclassified(100);unclassified(100);unclassified(100);
543	1	1	0	0	Bacteria(100);unclassified(100);unclassified(100);unclassified(100);unclassified(100);unclassified(100);
544	1	1	0	0	Bacteria(100);Firmicutes(100);Clostridia(100);Clostridiales(100);unclassified(100);unclassified(100);
545	1	1	0	0	Bacteria(100);"Actinobacteria"(100);Actinobacteria(100);Actinomycetales(100);unclassified(100);unclassified(100);
546	1	1	0	0	Bacteria(100);unclassified(100);unclassified(100);unclassified(100);unclassified(100);unclassified(100);
547	1	1	0	0	Bacteria(100);Firmicutes(100);Erysipelotrichia(100);Erysipelotrichales(100);Erysipelotrichaceae(100);Bulleidia(100);
548	1	1	0	0	Bacteria(100);"Actinobacteria"(100);Actinobacteria(100);Bifidobacteriales(100);Bifidobacteriaceae(100);Gardnerella(100);
549	1	1	0	0	Bacteria(100);Firmicutes(100);Bacilli(100);Lactobacillales(100);Streptococcaceae(100);Streptococcus(100);
550	1	1	0	0	Bacteria(100);"Proteobacteria"(100);Betaproteobacteria(100);Rhodocyclales(100);Rhodocyclaceae(100);Zoogloea(100);
551	1	1	0	0	Bacteria(100);Firmicutes(100);Clostridia(100);Clostridiales(100);unclassified(100);unclassified(100);
552	1	1	0	0	Bacteria(100);"Proteobacteria"(100);Gammaproteobacteria(100);"Enterobacteriales"(100);Enterobacteriaceae(100);Escherichia_Shigella(100);
553	1	1	0	0	Bacteria(100);"Bacteroidetes"(100);Flavobacteria(100);"Flavobacteriales"(100);Flavobacteriaceae(100);Chryseobacterium(100);
554	1	1	0	0	Bacteria(100);unclassified(100);unclassified(100);unclassified(100);unclassified(100);unclassified(100);
555	1	1	0	0	Bacteria(100);"Proteobacteria"(100);Alphaproteobacteria(100);unclassified(100);unclassified(100);unclassified(100);

556	1	1	0	0	Bacteria(100);"Proteobacteria"(100);Alphaproteobacteria(100);Rhizobiales(100);unclassified(100);unclassified(100);
557	1	1	0	0	Bacteria(100);Firmicutes(100);Bacilli(100);Lactobacillales(100);Streptococcaceae(100);Streptococcus(100);
558	1	1	0	0	Bacteria(100);Firmicutes(100);unclassified(100);unclassified(100);unclassified(100);unclassified(100);
559	1	1	0	0	Bacteria(100);unclassified(100);unclassified(100);unclassified(100);unclassified(100);unclassified(100);
560	1	1	0	0	Bacteria(100);"Proteobacteria"(100);Gammaproteobacteria(100);unclassified(100);unclassified(100);unclassified(100);
561	1	1	0	0	Bacteria(100);"Proteobacteria"(100);Alphaproteobacteria(100);Rhizobiales(100);unclassified(100);unclassified(100);
562	1	1	0	0	Bacteria(100);"Proteobacteria"(100);Epsilonproteobacteria(100);Campylobacterales(100);Campylobacteraceae(100);Campylobacter(100);
563	1	1	0	0	Bacteria(100);"Bacteroidetes"(100);"Bacteroidia"(100);"Bacteroidales"(100);"Prevotellaceae"(100);Prevotella(100);
564	1	1	0	0	Bacteria(100);"Bacteroidetes"(100);Flavobacteria(100);"Flavobacteriales"(100);Flavobacteriaceae(100);Flavobacterium(100);
565	1	1	0	0	Bacteria(100);Firmicutes(100);Bacilli(100);Lactobacillales(100);Streptococcaceae(100);Streptococcus(100);
566	1	1	0	0	Bacteria(100);"Chloroflexi"(100);unclassified(100);unclassified(100);unclassified(100);unclassified(100);
567	1	1	0	0	Bacteria(100);"Proteobacteria"(100);Alphaproteobacteria(100);Rhodospirillales(100);Acetobacteraceae(100);unclassified(100);
568	1	1	0	0	Bacteria(100);unclassified(100);unclassified(100);unclassified(100);unclassified(100);unclassified(100);
569	1	1	0	0	Bacteria(100);unclassified(100);unclassified(100);unclassified(100);unclassified(100);unclassified(100);
570	2	2	0	0	Bacteria(100);"Proteobacteria"(100);Gammaproteobacteria(100);"Enterobacteriales"(100);Enterobacteriaceae(100);unclassified(100);
571	1	1	0	0	Bacteria(100);"Proteobacteria"(100);Deltaproteobacteria(100);Bdellovibrionales(100);Bacteriovoracaceae(100);Peredibacter(100);
572	1	1	0	0	Bacteria(100);"Bacteroidetes"(100);"Sphingobacteria"(100);"Sphingobacteriales"(100);Sphingobacteriaceae(100);unclassified(100);
573	1	1	0	0	Bacteria(100);Firmicutes(100);Bacilli(100);Bacillales(100);Staphylococcaceae(100);Staphylococcus(100);
574	1	1	0	0	Bacteria(100);"Proteobacteria"(100);Betaproteobacteria(100);Neisseriales(100);Neisseriaceae(100);Bergeriella(100);
575	4	1	0	3	Bacteria(100);Firmicutes(100);Bacilli(100);Bacillales(100);Listeriaceae(100);Brochothrix(100);
576	1	1	0	0	Bacteria(100);unclassified(100);unclassified(100);unclassified(100);unclassified(100);unclassified(100);
577	1	1	0	0	Bacteria(100);Firmicutes(100);Clostridia(100);Clostridiales(100);Lachnospiraceae(100);unclassified(100);
578	1	1	0	0	Bacteria(100);"Bacteroidetes"(100);unclassified(100);unclassified(100);unclassified(100);unclassified(100);
579	1	1	0	0	Bacteria(100);"Fusobacteria"(100);"Fusobacteria"(100);"Fusobacteriales"(100);"Leptotrichiaceae"(100);Leptotrichia(100);
580	1	1	0	0	Bacteria(100);"Proteobacteria"(100);Gammaproteobacteria(100);unclassified(100);unclassified(100);unclassified(100);
581	1	1	0	0	Bacteria(100);"Proteobacteria"(100);Gammaproteobacteria(100);"Enterobacteriales"(100);Enterobacteriaceae(100);unclassified(100);
582	1	1	0	0	Bacteria(100);"Bacteroidetes"(100);Flavobacteria(100);"Flavobacteriales"(100);Flavobacteriaceae(100);Wautersiella(100);
583	1	1	0	0	Bacteria(100);Firmicutes(100);Bacilli(100);Bacillales(100);unclassified(100);unclassified(100);

584	1	1	0	0	Bacteria(100);"Proteobacteria"(100);Alphaproteobacteria(100);Sphingomonadales(100);Sphingomonadaceae(100);Blastomonas(100);
585	1	1	0	0	Bacteria(100);Firmicutes(100);Clostridia(100);Clostridiales(100);Ruminococcaceae(100);Ruminococcus(100);
586	1	1	0	0	Bacteria(100);Firmicutes(100);Bacilli(100);unclassified(100);unclassified(100);unclassified(100);
587	1	1	0	0	Bacteria(100);"Actinobacteria"(100);Actinobacteria(100);Actinomycetales(100);Actinomycetaceae(100);Actinomyces(100);
588	2	2	0	0	Bacteria(100);Firmicutes(100);Clostridia(100);Clostridiales(100);Ruminococcaceae(100);unclassified(100);
589	1	1	0	0	Bacteria(100);unclassified(100);unclassified(100);unclassified(100);unclassified(100);unclassified(100);
590	1	1	0	0	Bacteria(100);Firmicutes(100);Bacilli(100);unclassified(100);unclassified(100);unclassified(100);
591	1	1	0	0	Bacteria(100);Firmicutes(100);Bacilli(100);Lactobacillales(100);Carnobacteriaceae(100);Granulicatella(100);
592	1	1	0	0	Bacteria(100);Firmicutes(100);Bacilli(100);Lactobacillales(100);Streptococcaceae(100);Streptococcus(100);
593	1	1	0	0	Bacteria(100);"Bacteroidetes"(100);"Sphingobacteria"(100);"Sphingobacteriales"(100);Sphingobacteriaceae(100);unclassified(100);
594	1	1	0	0	Bacteria(100);unclassified(100);unclassified(100);unclassified(100);unclassified(100);unclassified(100);
595	2	2	0	0	Bacteria(100);Firmicutes(100);Bacilli(100);Lactobacillales(100);Streptococcaceae(100);Streptococcus(100);
596	1	1	0	0	Bacteria(100);"Fusobacteria"(100);"Fusobacteria"(100);"Fusobacteriales"(100);"Fusobacteriaceae"(100);Fusobacterium(100);
597	1	1	0	0	Bacteria(100);TM7(100);TM7_class_incertae_sedis(100);TM7_order_incertae_sedis(100);TM7_family_incertae_sedis(100);TM7_genus_incertae_sedis(100);
598	1	1	0	0	Bacteria(100);"Proteobacteria"(100);Betaproteobacteria(100);Neisseriales(100);Neisseriaceae(100);Neisseria(100);
599	1	1	0	0	Bacteria(100);"Proteobacteria"(100);unclassified(100);unclassified(100);unclassified(100);unclassified(100);
600	1	1	0	0	Bacteria(100);Firmicutes(100);Bacilli(100);Bacillales(100);Staphylococcaceae(100);unclassified(100);
601	1	1	0	0	Bacteria(100);"Actinobacteria"(100);Actinobacteria(100);Actinomycetales(100);unclassified(100);unclassified(100);
602	1	1	0	0	Bacteria(100);"Proteobacteria"(100);Betaproteobacteria(100);Burkholderiales(100);Comamonadaceae(100);unclassified(100);
603	1	1	0	0	Bacteria(100);Firmicutes(100);Clostridia(100);Clostridiales(100);Lachnospiraceae(100);Anaerostipes(100);
604	1	1	0	0	Bacteria(100);"Proteobacteria"(100);Betaproteobacteria(100);Neisseriales(100);Neisseriaceae(100);unclassified(100);
605	3	3	0	0	Bacteria(100);"Proteobacteria"(100);Gammaproteobacteria(100);"Enterobacteriales"(100);Enterobacteriaceae(100);Escherichia_Shigella(100);
606	1	1	0	0	Bacteria(100);"Proteobacteria"(100);Betaproteobacteria(100);Burkholderiales(100);Comamonadaceae(100);unclassified(100);
607	1	1	0	0	Bacteria(100);Firmicutes(100);Bacilli(100);unclassified(100);unclassified(100);unclassified(100);
608	1	1	0	0	Bacteria(100);TM7(100);TM7_class_incertae_sedis(100);TM7_order_incertae_sedis(100);TM7_family_incertae_sedis(100);TM7_genus_incertae_sedis(100);
609	1	1	0	0	Bacteria(100);unclassified(100);unclassified(100);unclassified(100);unclassified(100);unclassified(100);
610	1	1	0	0	Bacteria(100);Firmicutes(100);Bacilli(100);Lactobacillales(100);Streptococcaceae(100);Streptococcus(100);
611	1	1	0	0	Bacteria(100);unclassified(100);unclassified(100);unclassified(100);unclassified(100);unclassified(100);
612	1	1	0	0	Bacteria(100);"Actinobacteria"(100);Actinobacteria(100);Actinomycetales(100);Nocardioidaceae(100);unclassified(100);

613	1	1	0	0	Bacteria(100);"Fusobacteria"(100);"Fusobacteria"(100);"Fusobacteriales"(100);"Leptotrichiaceae"(100);Leptotrichia(100);
614	2	1	1	0	Bacteria(100);unclassified(100);unclassified(100);unclassified(100);unclassified(100);unclassified(100);
615	1	1	0	0	Bacteria(100);Firmicutes(100);Clostridia(100);Clostridiales(100);Clostridiales_Incertae_Sedis_XI(100);Anaerococcus(100);
616	1	1	0	0	Bacteria(100);"Proteobacteria"(100);Gammaproteobacteria(100);Xanthomonadales(100);Xanthomonadaceae(100);Pseudoxanthomonas(100);
617	1	1	0	0	Bacteria(100);Firmicutes(100);Bacilli(100);Lactobacillales(100);unclassified(100);unclassified(100);
618	1	1	0	0	Bacteria(100);"Fusobacteria"(100);"Fusobacteria"(100);"Fusobacteriales"(100);"Fusobacteriaceae"(100);Fusobacterium(100);
619	1	1	0	0	Bacteria(100);"Bacteroidetes"(100);Flavobacteria(100);"Flavobacteriales"(100);Flavobacteriaceae(100);Chryseobacterium(100);
620	1	1	0	0	Bacteria(100);"Proteobacteria"(100);Alphaproteobacteria(100);Sphingomonadales(100);Sphingomonadaceae(100);unclassified(100);
621	1	1	0	0	Bacteria(100);unclassified(100);unclassified(100);unclassified(100);unclassified(100);unclassified(100);
622	1	1	0	0	Bacteria(100);"Proteobacteria"(100);Gammaproteobacteria(100);Pseudomonadales(100);Pseudomonadaceae(100);unclassified(100);
623	1	1	0	0	Bacteria(100);"Proteobacteria"(100);Gammaproteobacteria(100);unclassified(100);unclassified(100);unclassified(100);
624	1	1	0	0	Bacteria(100);unclassified(100);unclassified(100);unclassified(100);unclassified(100);unclassified(100);
625	1	1	0	0	Bacteria(100);Firmicutes(100);Clostridia(100);Clostridiales(100);Peptostreptococcaceae(100);Clostridium_XI(100);
626	1	1	0	0	Bacteria(100);"Fusobacteria"(100);"Fusobacteria"(100);"Fusobacteriales"(100);"Fusobacteriaceae"(100);unclassified(100);
627	1	1	0	0	Bacteria(100);"Proteobacteria"(100);Betaproteobacteria(100);Burkholderiales(100);Alcaligenaceae(100);unclassified(100);
628	1	1	0	0	Bacteria(100);"Proteobacteria"(100);Deltaproteobacteria(100);Myxococcales(100);Polyangiaceae(100);Chondromyces(100);
629	1	1	0	0	Bacteria(100);unclassified(100);unclassified(100);unclassified(100);unclassified(100);unclassified(100);
630	1	1	0	0	Bacteria(100);Firmicutes(100);Clostridia(100);Clostridiales(100);unclassified(100);unclassified(100);
631	1	1	0	0	Bacteria(100);unclassified(100);unclassified(100);unclassified(100);unclassified(100);unclassified(100);
632	1	1	0	0	Bacteria(100);unclassified(100);unclassified(100);unclassified(100);unclassified(100);unclassified(100);
633	1	1	0	0	Bacteria(100);unclassified(100);unclassified(100);unclassified(100);unclassified(100);unclassified(100);
634	2	2	0	0	Bacteria(100);"Actinobacteria"(100);Actinobacteria(100);Actinomycetales(100);Nocardioidaceae(100);Marmoricola(100);
635	1	1	0	0	Bacteria(100);"Proteobacteria"(100);Alphaproteobacteria(100);unclassified(100);unclassified(100);unclassified(100);
636	1	1	0	0	Bacteria(100);"Proteobacteria"(100);Alphaproteobacteria(100);Rhodobacterales(100);Rhodobacteraceae(100);Haematobacter(100);
637	1	1	0	0	Bacteria(100);"Actinobacteria"(100);Actinobacteria(100);Actinomycetales(100);unclassified(100);unclassified(100);
638	1	1	0	0	Bacteria(100);unclassified(100);unclassified(100);unclassified(100);unclassified(100);unclassified(100);

639	1	1	0	0	Bacteria(100);"Proteobacteria"(100);Alphaproteobacteria(100);Sphingomonadales(100);unclassified(100);unclassified(100);
640	1	1	0	0	Bacteria(100);Firmicutes(100);Bacilli(100);Lactobacillales(100);Streptococcaceae(100);Streptococcus(100);
641	1	1	0	0	Bacteria(100);unclassified(100);unclassified(100);unclassified(100);unclassified(100);unclassified(100);
642	1	1	0	0	Bacteria(100);"Proteobacteria"(100);Alphaproteobacteria(100);unclassified(100);unclassified(100);unclassified(100);
643	1	1	0	0	Bacteria(100);unclassified(100);unclassified(100);unclassified(100);unclassified(100);unclassified(100);
644	1	1	0	0	Bacteria(100);Firmicutes(100);Clostridia(100);Clostridiales(100);Ruminococcaceae(100);unclassified(100);
645	1	1	0	0	Bacteria(100);"Proteobacteria"(100);Alphaproteobacteria(100);Sphingomonadales(100);Sphingomonadaceae(100);Novosphingobium(100);
646	1	1	0	0	Bacteria(100);"Proteobacteria"(100);Gammaproteobacteria(100);unclassified(100);unclassified(100);unclassified(100);
647	3	3	0	0	Bacteria(100);"Gemmatimonadetes"(100);Gemmatimonadetes(100);Gemmatimonadales(100);Gemmatimonadaceae(100);Gemmatimonas(100);
648	1	1	0	0	Bacteria(100);unclassified(100);unclassified(100);unclassified(100);unclassified(100);unclassified(100);
649	1	1	0	0	Bacteria(100);unclassified(100);unclassified(100);unclassified(100);unclassified(100);unclassified(100);
650	1	1	0	0	Bacteria(100);Firmicutes(100);Bacilli(100);Lactobacillales(100);Streptococcaceae(100);Streptococcus(100);
651	1	1	0	0	Bacteria(100);Firmicutes(100);Clostridia(100);Clostridiales(100);unclassified(100);unclassified(100);
652	1	1	0	0	Bacteria(100);unclassified(100);unclassified(100);unclassified(100);unclassified(100);unclassified(100);
653	1	1	0	0	Bacteria(100);Firmicutes(100);Bacilli(100);Lactobacillales(100);Carnobacteriaceae(100);unclassified(100);
654	1	1	0	0	Bacteria(100);OD1(100);OD1_class_incertae_sedis(100);OD1_order_incertae_sedis(100);OD1_family_incertae_sedis(100);OD1_genus_incertae_sedis(100);
655	1	1	0	0	Bacteria(100);unclassified(100);unclassified(100);unclassified(100);unclassified(100);unclassified(100);
656	2	2	0	0	Bacteria(100);"Verrucomicrobia"(100);Subdivision5(100);Subdivision5_order_incertae_sedis(100);Subdivision5_family_incertae_sedis(100);5_genus_incertae_sedis(100);
657	1	1	0	0	Bacteria(100);"Actinobacteria"(100);Actinobacteria(100);Actinomycetales(100);unclassified(100);unclassified(100);
658	1	1	0	0	Bacteria(100);unclassified(100);unclassified(100);unclassified(100);unclassified(100);unclassified(100);
659	1	1	0	0	Bacteria(100);"Proteobacteria"(100);Epsilonproteobacteria(100);Campylobacterales(100);Helicobacteraceae(100);Wolinella(100);
660	1	1	0	0	Bacteria(100);unclassified(100);unclassified(100);unclassified(100);unclassified(100);unclassified(100);
661	1	1	0	0	Bacteria(100);"Acidobacteria"(100);Acidobacteria_Gp4(100);Acidobacteria_Gp4_order_incertae_sedis(100);Acidobacteria_Gp4_family_incertae_sedis(100);Gp4(100);
662	1	1	0	0	Bacteria(100);"Fusobacteria"(100);"Fusobacteria"(100);"Fusobacteriales"(100);"Fusobacteriaceae"(100);unclassified(100);
663	1	1	0	0	Bacteria(100);"Actinobacteria"(100);Actinobacteria(100);Actinomycetales(100);Propionibacteriaceae(100);Aestuariimicrobium(100);
664	1	1	0	0	Bacteria(100);Firmicutes(100);Clostridia(100);Clostridiales(100);Clostridiales_Incertae_Sedis_XI(100);Anaerococcus(100);
665	1	1	0	0	Bacteria(100);unclassified(100);unclassified(100);unclassified(100);unclassified(100);unclassified(100);
666	1	1	0	0	Bacteria(100);Firmicutes(100);Bacilli(100);Lactobacillales(100);unclassified(100);unclassified(100);

667	1	1	0	0	Bacteria(100);"Fusobacteria"(100);"Fusobacteria"(100);"Fusobacteriales"(100);"Fusobacteriaceae"(100);Fusobacterium(100);<br>Bacteria(100);"Proteobacteria"(100);Gammaproteobacteria(100);Pseudomonadales(100);Pseudomonadaceae(100);unclassified(100)
668	1	1	0	0	;
669	1	1	0	0	Bacteria(100);unclassified(100);unclassified(100);unclassified(100);unclassified(100);unclassified(100);
670	1	1	0	0	Bacteria(100);unclassified(100);unclassified(100);unclassified(100);unclassified(100);unclassified(100);
671	1	1	0	0	Bacteria(100);"Actinobacteria"(100);Actinobacteria(100);Actinomycetales(100);Nocardiaceae(100);Rhodococcus(100);
672	1	1	0	0	Bacteria(100);"Bacteroidetes"(100);"Bacteroidia"(100);"Bacteroidales"(100);"Prevotellaceae"(100);Prevotella(100);
673	1	1	0	0	Bacteria(100);Firmicutes(100);Bacilli(100);unclassified(100);unclassified(100);unclassified(100);
674	1	1	0	0	Bacteria(100);"Fusobacteria"(100);"Fusobacteria"(100);"Fusobacteriales"(100);"Fusobacteriaceae"(100);Cetobacterium(100);
675	1	1	0	0	Bacteria(100);"Fusobacteria"(100);"Fusobacteria"(100);"Fusobacteriales"(100);"Fusobacteriaceae"(100);Fusobacterium(100);
676	1	1	0	0	Bacteria(100);Firmicutes(100);Negativicutes(100);Selenomonadales(100);Veillonellaceae(100);Veillonella(100);
677	1	1	0	0	Bacteria(100);"Bacteroidetes"(100);Flavobacteria(100);"Flavobacteriales"(100);Flavobacteriaceae(100);Capnocytophaga(100);
678	1	1	0	0	Bacteria(100);"Proteobacteria"(100);Betaproteobacteria(100);Neisseriales(100);Neisseriaceae(100);unclassified(100);
679	1	1	0	0	Bacteria(100);Firmicutes(100);Bacilli(100);Lactobacillales(100);Streptococcaceae(100);Streptococcus(100);
680	1	1	0	0	Bacteria(100);Firmicutes(100);Clostridia(100);Clostridiales(100);Clostridiaceae_1(100);unclassified(100);
681	3	3	0	0	Bacteria(100);Firmicutes(100);Bacilli(100);Lactobacillales(100);Carnobacteriaceae(100);unclassified(100);
682	1	1	0	0	Bacteria(100);"Proteobacteria"(100);unclassified(100);unclassified(100);unclassified(100);unclassified(100);
683	1	1	0	0	Bacteria(100);"Actinobacteria"(100);Actinobacteria(100);unclassified(100);unclassified(100);unclassified(100);
684	1	1	0	0	Bacteria(100);"Fusobacteria"(100);"Fusobacteria"(100);"Fusobacteriales"(100);"Fusobacteriaceae"(100);unclassified(100);
685	2	2	0	0	Bacteria(100);Firmicutes(100);Bacilli(100);Lactobacillales(100);Streptococcaceae(100);Streptococcus(100);
686	1	1	0	0	Bacteria(100);"Proteobacteria"(100);Betaproteobacteria(100);Neisseriales(100);Neisseriaceae(100);unclassified(100);
687	1	1	0	0	Bacteria(100);unclassified(100);unclassified(100);unclassified(100);unclassified(100);unclassified(100);
688	1	1	0	0	Bacteria(100);"Proteobacteria"(100);unclassified(100);unclassified(100);unclassified(100);unclassified(100);
689	1	1	0	0	Bacteria(100);"Bacteroidetes"(100);"Sphingobacteria"(100);"Sphingobacteriales"(100);Cytophagaceae(100);Hymenobacter(100);
690	1	1	0	0	Bacteria(100);Firmicutes(100);Bacilli(100);Lactobacillales(100);Carnobacteriaceae(100);Catellicoccus(100);
691	1	1	0	0	Bacteria(100);unclassified(100);unclassified(100);unclassified(100);unclassified(100);unclassified(100);
692	1	1	0	0	Bacteria(100);unclassified(100);unclassified(100);unclassified(100);unclassified(100);unclassified(100);
693	1	1	0	0	Bacteria(100);"Proteobacteria"(100);Gammaproteobacteria(100);Legionellales(100);Legionellaceae(100);Legionella(100);
694	1	1	0	0	Bacteria(100);"Actinobacteria"(100);Actinobacteria(100);Actinomycetales(100);unclassified(100);unclassified(100);
695	2	2	0	0	Bacteria(100);unclassified(100);unclassified(100);unclassified(100);unclassified(100);unclassified(100);

696	1	1	0	0	Bacteria(100);unclassified(100);unclassified(100);unclassified(100);unclassified(100);unclassified(100);
697	1	1	0	0	Bacteria(100);"Proteobacteria"(100);Alphaproteobacteria(100);Rhizobiales(100);unclassified(100);unclassified(100);
698	1	1	0	0	Bacteria(100);unclassified(100);unclassified(100);unclassified(100);unclassified(100);unclassified(100);
699	1	1	0	0	Bacteria(100);"Proteobacteria"(100);Alphaproteobacteria(100);Sphingomonadales(100);Sphingomonadaceae(100);Sphingobium(100);
700	1	1	0	0	Bacteria(100);"Proteobacteria"(100);Gammaproteobacteria(100);unclassified(100);unclassified(100);unclassified(100);
701	2	2	0	0	Bacteria(100);"Proteobacteria"(100);Betaproteobacteria(100);Burkholderiales(100);Sutterellaceae(100);Sutterella(100);
702	1	1	0	0	Bacteria(100);"Actinobacteria"(100);Actinobacteria(100);Actinomycetales(100);unclassified(100);unclassified(100);
703	1	1	0	0	Bacteria(100);unclassified(100);unclassified(100);unclassified(100);unclassified(100);unclassified(100);
704	1	1	0	0	Bacteria(100);"Bacteroidetes"(100);"Bacteroidia"(100);"Bacteroidales"(100);"Prevotellaceae"(100);Prevotella(100);
705	1	1	0	0	Bacteria(100);"Fusobacteria"(100);"Fusobacteria"(100);"Fusobacteriales"(100);"Leptotrichiaceae"(100);unclassified(100);
706	1	1	0	0	Bacteria(100);"Proteobacteria"(100);Betaproteobacteria(100);Neisseriales(100);Neisseriaceae(100);Neisseria(100);
707	1	1	0	0	Bacteria(100);"Actinobacteria"(100);Actinobacteria(100);Actinomycetales(100);unclassified(100);unclassified(100);
708	1	1	0	0	Bacteria(100);"Fusobacteria"(100);"Fusobacteria"(100);"Fusobacteriales"(100);"Fusobacteriaceae"(100);Cetobacterium(100);
709	1	1	0	0	Bacteria(100);"Proteobacteria"(100);Betaproteobacteria(100);Neisseriales(100);Neisseriaceae(100);unclassified(100);
710	1	1	0	0	Bacteria(100);Firmicutes(100);Bacilli(100);Lactobacillales(100);Lactobacillaceae(100);Lactobacillus(100);
711	1	1	0	0	Bacteria(100);"Proteobacteria"(100);Betaproteobacteria(100);Neisseriales(100);Neisseriaceae(100);unclassified(100);
712	1	1	0	0	Bacteria(100);"Fusobacteria"(100);"Fusobacteria"(100);"Fusobacteriales"(100);"Fusobacteriaceae"(100);Fusobacterium(100);
713	1	1	0	0	Bacteria(100);unclassified(100);unclassified(100);unclassified(100);unclassified(100);unclassified(100);
714	1	1	0	0	Bacteria(100);"Proteobacteria"(100);Gammaproteobacteria(100);Chromatiales(100);Halothiobacillaceae(100);Thiofaba(100);
715	1	1	0	0	Bacteria(100);Firmicutes(100);Bacilli(100);Lactobacillales(100);Streptococcaceae(100);Streptococcus(100);
716	1	1	0	0	Bacteria(100);unclassified(100);unclassified(100);unclassified(100);unclassified(100);unclassified(100);
717	1	1	0	0	Bacteria(100);Firmicutes(100);Bacilli(100);Lactobacillales(100);unclassified(100);unclassified(100);
718	1	1	0	0	Bacteria(100);Firmicutes(100);Bacilli(100);Lactobacillales(100);Streptococcaceae(100);Streptococcus(100);
719	1	1	0	0	Bacteria(100);unclassified(100);unclassified(100);unclassified(100);unclassified(100);unclassified(100);
720	1	1	0	0	Bacteria(100);unclassified(100);unclassified(100);unclassified(100);unclassified(100);unclassified(100);
721	1	1	0	0	Bacteria(100);"Fusobacteria"(100);"Fusobacteria"(100);"Fusobacteriales"(100);"Leptotrichiaceae"(100);Leptotrichia(100);
722	1	1	0	0	Bacteria(100);unclassified(100);unclassified(100);unclassified(100);unclassified(100);unclassified(100);
723	2	2	0	0	Bacteria(100);"Bacteroidetes"(100);"Bacteroidia"(100);"Bacteroidales"(100);Bacteroidaceae(100);Bacteroides(100);
724	1	1	0	0	Bacteria(100);"Fusobacteria"(100);"Fusobacteria"(100);"Fusobacteriales"(100);"Fusobacteriaceae"(100);unclassified(100);
725	1	1	0	0	Bacteria(100);unclassified(100);unclassified(100);unclassified(100);unclassified(100);unclassified(100);

726	2	2	0	0	Bacteria(100);unclassified(100);unclassified(100);unclassified(100);unclassified(100);unclassified(100);
727	1	1	0	0	Bacteria(100);Firmicutes(100);Bacilli(100);Bacillales(100);Staphylococcaceae(100);unclassified(100);
728	1	1	0	0	Bacteria(100);"Actinobacteria"(100);Actinobacteria(100);Actinomycetales(100);Actinomycetaceae(100);Actinomyces(100);
729	1	1	0	0	Bacteria(100);Firmicutes(100);Negativicutes(100);Selenomonadales(100);Veillonellaceae(100);Veillonella(100);
730	1	1	0	0	Bacteria(100);unclassified(100);unclassified(100);unclassified(100);unclassified(100);unclassified(100);
731	1	1	0	0	Bacteria(100);"Proteobacteria"(100);Gammaproteobacteria(100);Xanthomonadales(100);Xanthomonadaceae(100);Frateuria(100);
732	1	1	0	0	Bacteria(100);"Fusobacteria"(100);"Fusobacteria"(100);"Fusobacteriales"(100);"Fusobacteriaceae"(100);Fusobacterium(100);
733	1	1	0	0	Bacteria(100);Firmicutes(100);Bacilli(100);Lactobacillales(100);Streptococcaceae(100);Streptococcus(100);
734	1	1	0	0	Bacteria(100);Firmicutes(100);Clostridia(100);Clostridiales(100);unclassified(100);unclassified(100);
735	1	1	0	0	Bacteria(100);"Proteobacteria"(100);Gammaproteobacteria(100);Pseudomonadales(100);Moraxellaceae(100);Acinetobacter(100);
736	1	1	0	0	Bacteria(100);Firmicutes(100);Clostridia(100);Clostridiales(100);unclassified(100);unclassified(100);
737	1	1	0	0	Bacteria(100);"Proteobacteria"(100);Betaproteobacteria(100);Neisseriales(100);Neisseriaceae(100);unclassified(100);
738	2	2	0	0	Bacteria(100);OD1(100);OD1_class_incertae_sedis(100);OD1_order_incertae_sedis(100);OD1_family_incertae_sedis(100);OD1_genus_incertae_sedis(100);
739	2	2	0	0	Bacteria(100);"Proteobacteria"(100);Alphaproteobacteria(100);Rhizobiales(100);unclassified(100);unclassified(100);
740	1	1	0	0	Bacteria(100);Firmicutes(100);Bacilli(100);Bacillales(100);Staphylococcaceae(100);Staphylococcus(100);
741	1	1	0	0	Bacteria(100);unclassified(100);unclassified(100);unclassified(100);unclassified(100);unclassified(100);
742	2	2	0	0	Bacteria(100);"Proteobacteria"(100);Gammaproteobacteria(100);"Enterobacteriales"(100);Enterobacteriaceae(100);unclassified(100);
743	1	1	0	0	Bacteria(100);unclassified(100);unclassified(100);unclassified(100);unclassified(100);unclassified(100);
744	1	1	0	0	Bacteria(100);unclassified(100);unclassified(100);unclassified(100);unclassified(100);unclassified(100);
745	1	1	0	0	Bacteria(100);"Verrucomicrobia"(100);Verrucomicrobiae(100);Verrucomicrobiales(100);Verrucomicrobiaceae(100);Prosthecobacter(100);
746	1	1	0	0	Bacteria(100);"Proteobacteria"(100);Alphaproteobacteria(100);Rhizobiales(100);"Aurantimonadaceae"(100);Aurantimonas(100);
747	1	1	0	0	Bacteria(100);unclassified(100);unclassified(100);unclassified(100);unclassified(100);unclassified(100);
748	1	1	0	0	Bacteria(100);unclassified(100);unclassified(100);unclassified(100);unclassified(100);unclassified(100);
749	1	1	0	0	Bacteria(100);unclassified(100);unclassified(100);unclassified(100);unclassified(100);unclassified(100);
750	1	1	0	0	Bacteria(100);unclassified(100);unclassified(100);unclassified(100);unclassified(100);unclassified(100);
751	1	1	0	0	Bacteria(100);"Proteobacteria"(100);Alphaproteobacteria(100);unclassified(100);unclassified(100);unclassified(100);
752	1	1	0	0	Bacteria(100);unclassified(100);unclassified(100);unclassified(100);unclassified(100);unclassified(100);
753	1	1	0	0	Bacteria(100);unclassified(100);unclassified(100);unclassified(100);unclassified(100);unclassified(100);
754	1	1	0	0	Bacteria(100);Firmicutes(100);Clostridia(100);Clostridiales(100);Clostridiaceae_1(100);unclassified(100);

755	1	1	0	0	Bacteria(100);Firmicutes(100);Clostridia(100);Clostridiales(100);Peptostreptococcaceae(100);unclassified(100);
756	1	1	0	0	Bacteria(100);unclassified(100);unclassified(100);unclassified(100);unclassified(100);unclassified(100);
757	1	1	0	0	Bacteria(100);unclassified(100);unclassified(100);unclassified(100);unclassified(100);unclassified(100);
758	1	1	0	0	Bacteria(100);unclassified(100);unclassified(100);unclassified(100);unclassified(100);unclassified(100);
759	1	1	0	0	Bacteria(100);Firmicutes(100);Clostridia(100);Clostridiales(100);Lachnospiraceae(100);unclassified(100);
760	1	1	0	0	Bacteria(100);unclassified(100);unclassified(100);unclassified(100);unclassified(100);unclassified(100);
761	1	1	0	0	Bacteria(100);unclassified(100);unclassified(100);unclassified(100);unclassified(100);unclassified(100);
762	1	1	0	0	Bacteria(100);unclassified(100);unclassified(100);unclassified(100);unclassified(100);unclassified(100);
763	1	1	0	0	Bacteria(100);unclassified(100);unclassified(100);unclassified(100);unclassified(100);unclassified(100);
764	1	1	0	0	Bacteria(100);"Actinobacteria"(100);Actinobacteria(100);Actinomycetales(100);Dermabacteraceae(100);unclassified(100);
765	1	1	0	0	Bacteria(100);Firmicutes(100);Bacilli(100);Lactobacillales(100);Streptococcaceae(100);Streptococcus(100);
766	1	1	0	0	Bacteria(100);Firmicutes(100);unclassified(100);unclassified(100);unclassified(100);unclassified(100);
767	1	1	0	0	Bacteria(100);"Fusobacteria"(100);"Fusobacteria"(100);"Fusobacteriales"(100);"Fusobacteriaceae"(100);Fusobacterium(100);
768	1	1	0	0	Bacteria(100);"Bacteroidetes"(100);Flavobacteria(100);"Flavobacteriales"(100);Flavobacteriaceae(100);Capnocytophaga(100);
769	1	1	0	0	Bacteria(100);unclassified(100);unclassified(100);unclassified(100);unclassified(100);unclassified(100);
770	1	1	0	0	Bacteria(100);"Proteobacteria"(100);Betaproteobacteria(100);unclassified(100);unclassified(100);unclassified(100);
771	1	1	0	0	Bacteria(100);"Proteobacteria"(100);Alphaproteobacteria(100);Sphingomonadales(100);Erythrobacteraceae(100);Erythrobacter(100) ;
772	2	2	0	0	Bacteria(100);"Actinobacteria"(100);Actinobacteria(100);Actinomycetales(100);Microbacteriaceae(100);Rathayibacter(100);
773	1	1	0	0	Bacteria(100);"Actinobacteria"(100);Actinobacteria(100);Actinomycetales(100);Micrococcaceae(100);Rothia(100);
774	1	1	0	0	Bacteria(100);"Actinobacteria"(100);Actinobacteria(100);Actinomycetales(100);Pseudonocardiaceae(100);unclassified(100);
775	5	5	0	0	Bacteria(100);Firmicutes(100);Erysipelotrichia(100);Erysipelotrichales(100);Erysipelotrichaceae(100);Catenibacterium(100);
776	1	1	0	0	Bacteria(100);"Actinobacteria"(100);Actinobacteria(100);Actinomycetales(100);Micrococcaceae(100);Rothia(100);
777	1	1	0	0	Bacteria(100);Firmicutes(100);Bacilli(100);Lactobacillales(100);Streptococcaceae(100);Streptococcus(100);
778	1	1	0	0	Bacteria(100);"Fusobacteria"(100);"Fusobacteria"(100);"Fusobacteriales"(100);"Fusobacteriaceae"(100);Fusobacterium(100);
779	1	1	0	0	Bacteria(100);unclassified(100);unclassified(100);unclassified(100);unclassified(100);unclassified(100);
780	1	1	0	0	Bacteria(100);Firmicutes(100);Bacilli(100);Lactobacillales(100);unclassified(100);unclassified(100);
781	1	1	0	0	Bacteria(100);unclassified(100);unclassified(100);unclassified(100);unclassified(100);unclassified(100);
782	1	1	0	0	Bacteria(100);Firmicutes(100);Negativicutes(100);Selenomonadales(100);Veillonellaceae(100);unclassified(100);
783	1	1	0	0	Bacteria(100);unclassified(100);unclassified(100);unclassified(100);unclassified(100);unclassified(100);

784	1	1	0	0	Bacteria(100);"Proteobacteria"(100);Betaproteobacteria(100);Neisseriales(100);Neisseriaceae(100);unclassified(100);
785	1	1	0	0	Bacteria(100);"Proteobacteria"(100);unclassified(100);unclassified(100);unclassified(100);unclassified(100);
786	1	1	0	0	Bacteria(100);Firmicutes(100);Bacilli(100);Lactobacillales(100);Carnobacteriaceae(100);unclassified(100);
787	1	1	0	0	Bacteria(100);"Fusobacteria"(100);"Fusobacteria"(100);"Fusobacteriales"(100);"Fusobacteriaceae"(100);unclassified(100);
788	1	1	0	0	Bacteria(100);"Actinobacteria"(100);Actinobacteria(100);Actinomycetales(100);Microbacteriaceae(100);Leucobacter(100);
789	1	1	0	0	Bacteria(100);"Fusobacteria"(100);"Fusobacteria"(100);"Fusobacteriales"(100);"Leptotrichiaceae"(100);Leptotrichia(100);
790	1	1	0	0	Bacteria(100);Firmicutes(100);Bacilli(100);Lactobacillales(100);Streptococcaceae(100);Streptococcus(100);
791	1	1	0	0	Bacteria(100);unclassified(100);unclassified(100);unclassified(100);unclassified(100);unclassified(100);
792	1	1	0	0	Bacteria(100);unclassified(100);unclassified(100);unclassified(100);unclassified(100);unclassified(100);
793	1	1	0	0	Bacteria(100);"Bacteroidetes"(100);"Bacteroidia"(100);"Bacteroidales"(100);"Prevotellaceae"(100);Prevotella(100);
794	4	4	0	0	Bacteria(100);TM7(100);TM7_class_incertae_sedis(100);TM7_order_incertae_sedis(100);TM7_family_incertae_sedis(100);TM7_genus_incertae_sedis(100);
795	1	1	0	0	Bacteria(100);unclassified(100);unclassified(100);unclassified(100);unclassified(100);unclassified(100);
796	1	1	0	0	Bacteria(100);"Actinobacteria"(100);Actinobacteria(100);Actinomycetales(100);unclassified(100);unclassified(100);
797	1	1	0	0	Bacteria(100);unclassified(100);unclassified(100);unclassified(100);unclassified(100);unclassified(100);
798	1	1	0	0	Bacteria(100);unclassified(100);unclassified(100);unclassified(100);unclassified(100);unclassified(100);
799	1	1	0	0	Bacteria(100);"Proteobacteria"(100);Gammaproteobacteria(100);"Enterobacteriales"(100);Enterobacteriaceae(100);unclassified(100);
800	1	1	0	0	Bacteria(100);Firmicutes(100);Bacilli(100);Lactobacillales(100);Streptococcaceae(100);Streptococcus(100);
801	7	2	0	5	Bacteria(100);"Actinobacteria"(100);Actinobacteria(100);Actinomycetales(100);Nocardioidaceae(100);Nocardioides(100);
802	1	1	0	0	Bacteria(100);Firmicutes(100);Bacilli(100);unclassified(100);unclassified(100);unclassified(100);
803	1	1	0	0	Bacteria(100);Firmicutes(100);Clostridia(100);Clostridiales(100);unclassified(100);unclassified(100);
804	1	1	0	0	Bacteria(100);Firmicutes(100);Bacilli(100);Lactobacillales(100);unclassified(100);unclassified(100);
805	1	1	0	0	Bacteria(100);"Fusobacteria"(100);"Fusobacteria"(100);"Fusobacteriales"(100);"Fusobacteriaceae"(100);Fusobacterium(100);
806	1	1	0	0	Bacteria(100);unclassified(100);unclassified(100);unclassified(100);unclassified(100);unclassified(100);
807	1	1	0	0	Bacteria(100);"Proteobacteria"(100);Betaproteobacteria(100);Neisseriales(100);Neisseriaceae(100);Neisseria(100);
808	1	1	0	0	Bacteria(100);"Actinobacteria"(100);Actinobacteria(100);Bifidobacteriales(100);Bifidobacteriaceae(100);Gardnerella(100);
809	1	1	0	0	Bacteria(100);unclassified(100);unclassified(100);unclassified(100);unclassified(100);unclassified(100);
810	1	1	0	0	Bacteria(100);unclassified(100);unclassified(100);unclassified(100);unclassified(100);unclassified(100);
811	1	1	0	0	Bacteria(100);unclassified(100);unclassified(100);unclassified(100);unclassified(100);unclassified(100);
812	1	1	0	0	Bacteria(100);"Proteobacteria"(100);Gammaproteobacteria(100);unclassified(100);unclassified(100);unclassified(100);

813	2	2	0	0	Bacteria(100);"Actinobacteria"(100);Actinobacteria(100);Actinomycetales(100);unclassified(100);unclassified(100);
814	1	1	0	0	Bacteria(100);"Proteobacteria"(100);Gammaproteobacteria(100);Pseudomonadales(100);Pseudomonadaceae(100);Cellvibrio(100);
815	1	1	0	0	Bacteria(100);unclassified(100);unclassified(100);unclassified(100);unclassified(100);unclassified(100);
816	1	1	0	0	Bacteria(100);Firmicutes(100);unclassified(100);unclassified(100);unclassified(100);unclassified(100);
817	1	1	0	0	Bacteria(100);unclassified(100);unclassified(100);unclassified(100);unclassified(100);unclassified(100);
818	1	1	0	0	Bacteria(100);"Proteobacteria"(100);Betaproteobacteria(100);Neisseriales(100);Neisseriaceae(100);unclassified(100);
819	1	1	0	0	Bacteria(100);unclassified(100);unclassified(100);unclassified(100);unclassified(100);unclassified(100);
820	1	1	0	0	Bacteria(100);unclassified(100);unclassified(100);unclassified(100);unclassified(100);unclassified(100);
821	1	1	0	0	Bacteria(100);"Bacteroidetes"(100);"Sphingobacteria"(100);"Sphingobacteriales"(100);Chitinophagaceae(100);unclassified(100);
822	1	1	0	0	Bacteria(100);unclassified(100);unclassified(100);unclassified(100);unclassified(100);unclassified(100);
823	1	1	0	0	Bacteria(100);"Proteobacteria"(100);Gammaproteobacteria(100);Pseudomonadales(100);Pseudomonadaceae(100);unclassified(100);
824	1	1	0	0	Bacteria(100);"Fusobacteria"(100);"Fusobacteria"(100);"Fusobacteriales"(100);"Fusobacteriaceae"(100);unclassified(100);
825	1	1	0	0	Bacteria(100);Firmicutes(100);Bacilli(100);Lactobacillales(100);Streptococcaceae(100);Streptococcus(100);
826	3	3	0	0	Bacteria(100);unclassified(100);unclassified(100);unclassified(100);unclassified(100);unclassified(100);
827	1	1	0	0	Bacteria(100);"Bacteroidetes"(100);"Bacteroidia"(100);"Bacteroidales"(100);"Prevotellaceae"(100);unclassified(100);
828	1	1	0	0	Bacteria(100);unclassified(100);unclassified(100);unclassified(100);unclassified(100);unclassified(100);
829	1	1	0	0	Bacteria(100);Firmicutes(100);Clostridia(100);Clostridiales(100);Peptostreptococcaceae(100);unclassified(100);
830	2	2	0	0	Bacteria(100);"Proteobacteria"(100);Epsilonproteobacteria(100);Campylobacterales(100);Campylobacteraceae(100);Campylobacter(100);
831	2	2	0	0	Bacteria(100);unclassified(100);unclassified(100);unclassified(100);unclassified(100);unclassified(100);
832	1	1	0	0	Bacteria(100);unclassified(100);unclassified(100);unclassified(100);unclassified(100);unclassified(100);
833	1	1	0	0	Bacteria(100);Firmicutes(100);Bacilli(100);Bacillales(100);Staphylococcaceae(100);Staphylococcus(100);
834	1	1	0	0	Bacteria(100);unclassified(100);unclassified(100);unclassified(100);unclassified(100);unclassified(100);
835	1	1	0	0	Bacteria(100);Firmicutes(100);Bacilli(100);unclassified(100);unclassified(100);unclassified(100);
836	1	1	0	0	Bacteria(100);unclassified(100);unclassified(100);unclassified(100);unclassified(100);unclassified(100);
837	1	1	0	0	Bacteria(100);"Proteobacteria"(100);Betaproteobacteria(100);Neisseriales(100);Neisseriaceae(100);unclassified(100);
838	1	1	0	0	Bacteria(100);unclassified(100);unclassified(100);unclassified(100);unclassified(100);unclassified(100);
839	1	1	0	0	Bacteria(100);Firmicutes(100);Bacilli(100);Lactobacillales(100);unclassified(100);unclassified(100);
840	1	1	0	0	Bacteria(100);Firmicutes(100);unclassified(100);unclassified(100);unclassified(100);unclassified(100);
841	1	1	0	0	Bacteria(100);"Proteobacteria"(100);Alphaproteobacteria(100);Sphingomonadales(100);unclassified(100);unclassified(100);
842	1	1	0	0	Bacteria(100);"Actinobacteria"(100);Actinobacteria(100);Actinomycetales(100);unclassified(100);unclassified(100);

843	1	1	0	0	Bacteria(100);"Proteobacteria"(100);Alphaproteobacteria(100);Sphingomonadales(100);Erythrobacteraceae(100);unclassified(100);
844	1	1	0	0	Bacteria(100);Firmicutes(100);Clostridia(100);Clostridiales(100);Clostridiaceae_1(100);unclassified(100);
845	1	1	0	0	Bacteria(100);"Actinobacteria"(100);Actinobacteria(100);Acidimicrobiales(100);unclassified(100);unclassified(100);
846	1	1	0	0	Bacteria(100);unclassified(100);unclassified(100);unclassified(100);unclassified(100);unclassified(100);
847	1	1	0	0	Bacteria(100);Firmicutes(100);Bacilli(100);Lactobacillales(100);unclassified(100);unclassified(100);
848	1	1	0	0	Bacteria(100);"Proteobacteria"(100);Gammaproteobacteria(100);Cardiobacteriales(100);Cardiobacteriaceae(100);Cardiobacterium(100);
849	1	1	0	0	Bacteria(100);Firmicutes(100);Clostridia(100);Clostridiales(100);unclassified(100);unclassified(100);
850	1	1	0	0	Bacteria(100);unclassified(100);unclassified(100);unclassified(100);unclassified(100);unclassified(100);
851	1	1	0	0	Bacteria(100);"Actinobacteria"(100);Actinobacteria(100);Actinomycetales(100);Micrococcaceae(100);Rothia(100);
852	1	1	0	0	Bacteria(100);"Fusobacteria"(100);"Fusobacteria"(100);"Fusobacteriales"(100);"Leptotrichiaceae"(100);Leptotrichia(100);
853	1	1	0	0	Bacteria(100);"Actinobacteria"(100);Actinobacteria(100);Coriobacteriales(100);Coriobacteriaceae(100);Atopobium(100);
854	1	1	0	0	Bacteria(100);"Proteobacteria"(100);Alphaproteobacteria(100);Rhizobiales(100);unclassified(100);unclassified(100);
855	1	1	0	0	Bacteria(100);"Proteobacteria"(100);Betaproteobacteria(100);Neisseriales(100);Neisseriaceae(100);unclassified(100);
856	1	1	0	0	Bacteria(100);"Proteobacteria"(100);Gammaproteobacteria(100);Acidithiobacillales(100);Acidithiobacillaceae(100);Acidithiobacillus(100);
857	1	1	0	0	Bacteria(100);"Fusobacteria"(100);"Fusobacteria"(100);"Fusobacteriales"(100);"Fusobacteriaceae"(100);Fusobacterium(100);
858	1	1	0	0	Bacteria(100);unclassified(100);unclassified(100);unclassified(100);unclassified(100);unclassified(100);
859	1	1	0	0	Bacteria(100);"Bacteroidetes"(100);Flavobacteria(100);"Flavobacteriales"(100);Cryomorphaceae(100);Fluviicola(100);
860	1	1	0	0	Bacteria(100);unclassified(100);unclassified(100);unclassified(100);unclassified(100);unclassified(100);
861	1	1	0	0	Bacteria(100);"Proteobacteria"(100);Betaproteobacteria(100);Rhodocyclales(100);Rhodocyclaceae(100);unclassified(100);
862	1	1	0	0	Bacteria(100);"Actinobacteria"(100);Actinobacteria(100);Actinomycetales(100);unclassified(100);unclassified(100);
863	1	1	0	0	Bacteria(100);unclassified(100);unclassified(100);unclassified(100);unclassified(100);unclassified(100);
864	2	2	0	0	Bacteria(100);Firmicutes(100);Clostridia(100);Clostridiales(100);unclassified(100);unclassified(100);
865	1	1	0	0	Bacteria(100);"Proteobacteria"(100);Gammaproteobacteria(100);"Enterobacteriales"(100);Enterobacteriaceae(100);unclassified(100);
866	1	1	0	0	Bacteria(100);unclassified(100);unclassified(100);unclassified(100);unclassified(100);unclassified(100);
867	1	1	0	0	Bacteria(100);Firmicutes(100);Negativicutes(100);Selenomonadales(100);Veillonellaceae(100);Dialister(100);
868	3	3	0	0	Bacteria(100);"Proteobacteria"(100);Betaproteobacteria(100);Burkholderiales(100);unclassified(100);unclassified(100);
869	1	1	0	0	Bacteria(100);unclassified(100);unclassified(100);unclassified(100);unclassified(100);unclassified(100);
870	1	1	0	0	Bacteria(100);unclassified(100);unclassified(100);unclassified(100);unclassified(100);unclassified(100);
871	1	1	0	0	Bacteria(100);"Actinobacteria"(100);Actinobacteria(100);Actinomycetales(100);Micrococcaceae(100);Rothia(100);

872	1	1	0	0	Bacteria(100);"Bacteroidetes"(100);"Bacteroidia"(100);"Bacteroidales"(100);"Prevotellaceae"(100);Prevotella(100);
873	1	1	0	0	Bacteria(100);"Actinobacteria"(100);Actinobacteria(100);Actinomycetales(100);Actinomycetaceae(100);Actinomyces(100);
874	2	2	0	0	Bacteria(100);"Actinobacteria"(100);Actinobacteria(100);Coriobacteriales(100);Coriobacteriaceae(100);Olsenella(100);
875	1	1	0	0	Bacteria(100);"Fusobacteria"(100);"Fusobacteria"(100);"Fusobacteriales"(100);"Fusobacteriaceae"(100);Cetobacterium(100);
876	1	1	0	0	Bacteria(100);"Actinobacteria"(100);Actinobacteria(100);Actinomycetales(100);Actinomycetaceae(100);Actinomyces(100);
877	1	1	0	0	Bacteria(100);Firmicutes(100);Negativicutes(100);Selenomonadales(100);Veillonellaceae(100);unclassified(100);
878	1	1	0	0	Bacteria(100);unclassified(100);unclassified(100);unclassified(100);unclassified(100);unclassified(100);
879	1	1	0	0	Bacteria(100);"Actinobacteria"(100);Actinobacteria(100);Actinomycetales(100);unclassified(100);unclassified(100);
880	1	1	0	0	Bacteria(100);"Actinobacteria"(100);Actinobacteria(100);Actinomycetales(100);unclassified(100);unclassified(100);
881	1	1	0	0	Bacteria(100);unclassified(100);unclassified(100);unclassified(100);unclassified(100);unclassified(100);
882	1	1	0	0	Bacteria(100);"Actinobacteria"(100);Actinobacteria(100);Actinomycetales(100);Microbacteriaceae(100);unclassified(100);
883	1	1	0	0	Bacteria(100);Firmicutes(100);Clostridia(100);Clostridiales(100);Clostridiaceae_1(100);unclassified(100);
884	1	1	0	0	Bacteria(100);unclassified(100);unclassified(100);unclassified(100);unclassified(100);unclassified(100);
885	1	1	0	0	Bacteria(100);unclassified(100);unclassified(100);unclassified(100);unclassified(100);unclassified(100);
886	1	1	0	0	Bacteria(100);unclassified(100);unclassified(100);unclassified(100);unclassified(100);unclassified(100);
887	1	1	0	0	Bacteria(100);unclassified(100);unclassified(100);unclassified(100);unclassified(100);unclassified(100);
888	1	1	0	0	Bacteria(100);OD1(100);OD1_class_incertae_sedis(100);OD1_order_incertae_sedis(100);OD1_family_incertae_sedis(100);OD1_genus_incertae_sedis(100);
889	1	1	0	0	Bacteria(100);Firmicutes(100);Bacilli(100);Lactobacillales(100);unclassified(100);unclassified(100);
890	1	1	0	0	Bacteria(100);Firmicutes(100);Clostridia(100);Clostridiales(100);Lachnospiraceae(100);Clostridium_XlVb(100);
891	1	1	0	0	Bacteria(100);"Actinobacteria"(100);Actinobacteria(100);Actinomycetales(100);Propionibacteriaceae(100);Friedmanniella(100);
892	1	1	0	0	Bacteria(100);unclassified(100);unclassified(100);unclassified(100);unclassified(100);unclassified(100);
893	1	1	0	0	Bacteria(100);"Fusobacteria"(100);"Fusobacteria"(100);"Fusobacteriales"(100);"Fusobacteriaceae"(100);Cetobacterium(100);
894	1	1	0	0	Bacteria(100);"Proteobacteria"(100);unclassified(100);unclassified(100);unclassified(100);unclassified(100);
895	1	1	0	0	Bacteria(100);Firmicutes(100);Clostridia(100);Clostridiales(100);Clostridiaceae_1(100);unclassified(100);
896	1	1	0	0	Bacteria(100);unclassified(100);unclassified(100);unclassified(100);unclassified(100);unclassified(100);
897	1	1	0	0	Bacteria(100);"Proteobacteria"(100);Gammaproteobacteria(100);unclassified(100);unclassified(100);unclassified(100);
898	1	1	0	0	Bacteria(100);Firmicutes(100);Bacilli(100);Lactobacillales(100);Streptococcaceae(100);Streptococcus(100);
899	1	1	0	0	Bacteria(100);"Actinobacteria"(100);Actinobacteria(100);Actinomycetales(100);Actinomycetaceae(100);unclassified(100);
900	1	1	0	0	Bacteria(100);"Proteobacteria"(100);Deltaproteobacteria(100);Myxococcales(100);unclassified(100);unclassified(100);

901	1	1	0	0	Bacteria(100);unclassified(100);unclassified(100);unclassified(100);unclassified(100);unclassified(100);
902	1	1	0	0	Bacteria(100);"Proteobacteria"(100);Gammaproteobacteria(100);Pasteurellales(100);Pasteurellaceae(100);unclassified(100);
903	1	1	0	0	Bacteria(100);unclassified(100);unclassified(100);unclassified(100);unclassified(100);unclassified(100);
904	1	1	0	0	Bacteria(100);"Actinobacteria"(100);Actinobacteria(100);Actinomycetales(100);unclassified(100);unclassified(100);
905	1	1	0	0	Bacteria(100);"Actinobacteria"(100);Actinobacteria(100);Actinomycetales(100);Nocardioidaceae(100);Nocardioides(100);
906	1	1	0	0	Bacteria(100);unclassified(100);unclassified(100);unclassified(100);unclassified(100);unclassified(100);
907	1	1	0	0	Bacteria(100);unclassified(100);unclassified(100);unclassified(100);unclassified(100);unclassified(100);
908	4	4	0	0	Bacteria(100);"Proteobacteria"(100);Alphaproteobacteria(100);Rhizobiales(100);Rhizobiaceae(100);Rhizobium(100);
909	1	1	0	0	Bacteria(100);Firmicutes(100);Clostridia(100);Clostridiales(100);Lachnospiraceae(100);unclassified(100);
910	1	1	0	0	Bacteria(100);"Actinobacteria"(100);Actinobacteria(100);Actinomycetales(100);Propionibacteriaceae(100);Propionibacterium(100);
911	1	1	0	0	Bacteria(100);Firmicutes(100);Bacilli(100);Lactobacillales(100);Streptococcaceae(100);Streptococcus(100);
912	1	1	0	0	Bacteria(100);Firmicutes(100);Bacilli(100);Bacillales(100);Staphylococcaceae(100);Staphylococcus(100);
913	1	1	0	0	Bacteria(100);"Proteobacteria"(100);Gammaproteobacteria(100);unclassified(100);unclassified(100);unclassified(100);
914	1	1	0	0	Bacteria(100);"Proteobacteria"(100);Gammaproteobacteria(100);Pseudomonadales(100);Pseudomonadaceae(100);unclassified(100);
915	1	1	0	0	Bacteria(100);"Fusobacteria"(100);"Fusobacteria"(100);"Fusobacteriales"(100);"Fusobacteriaceae"(100);Fusobacterium(100);
916	1	1	0	0	Bacteria(100);Firmicutes(100);Bacilli(100);Lactobacillales(100);unclassified(100);unclassified(100);
917	1	1	0	0	Bacteria(100);unclassified(100);unclassified(100);unclassified(100);unclassified(100);unclassified(100);
918	1	1	0	0	Bacteria(100);Firmicutes(100);Bacilli(100);Lactobacillales(100);Aerococcaceae(100);Dolosicoccus(100);
919	1	1	0	0	Bacteria(100);"Proteobacteria"(100);Betaproteobacteria(100);Neisseriales(100);Neisseriaceae(100);unclassified(100);
920	1	1	0	0	Bacteria(100);TM7(100);TM7_class_incertae_sedis(100);TM7_order_incertae_sedis(100);TM7_family_incertae_sedis(100);TM7_genus_incertae_sedis(100);
921	1	1	0	0	Bacteria(100);Firmicutes(100);Bacilli(100);Lactobacillales(100);Streptococcaceae(100);Streptococcus(100);
922	1	1	0	0	Bacteria(100);"Proteobacteria"(100);Betaproteobacteria(100);Neisseriales(100);Neisseriaceae(100);unclassified(100);
923	1	1	0	0	Bacteria(100);"Proteobacteria"(100);Alphaproteobacteria(100);Rhodospirillales(100);Acetobacteraceae(100);unclassified(100);
924	1	1	0	0	Bacteria(100);"Proteobacteria"(100);Betaproteobacteria(100);Burkholderiales(100);unclassified(100);unclassified(100);
925	1	1	0	0	Bacteria(100);"Proteobacteria"(100);Gammaproteobacteria(100);Xanthomonadales(100);Xanthomonadaceae(100);unclassified(100);
926	1	1	0	0	Bacteria(100);unclassified(100);unclassified(100);unclassified(100);unclassified(100);unclassified(100);
927	1	1	0	0	Bacteria(100);unclassified(100);unclassified(100);unclassified(100);unclassified(100);unclassified(100);
928	1	1	0	0	Bacteria(100);Firmicutes(100);Bacilli(100);Lactobacillales(100);unclassified(100);unclassified(100);
929	1	1	0	0	Bacteria(100);"Actinobacteria"(100);Actinobacteria(100);Actinomycetales(100);unclassified(100);unclassified(100);

930	1	1	0	0	Bacteria(100);"Proteobacteria"(100);Gammaproteobacteria(100);Pseudomonadales(100);Pseudomonadaceae(100);unclassified(100);
931	1	1	0	0	Bacteria(100);"Proteobacteria"(100);Gammaproteobacteria(100);"Enterobacteriales"(100);Enterobacteriaceae(100);unclassified(100);
932	1	1	0	0	Bacteria(100);"Proteobacteria"(100);Alphaproteobacteria(100);Sphingomonadales(100);unclassified(100);unclassified(100);
933	1	1	0	0	Bacteria(100);unclassified(100);unclassified(100);unclassified(100);unclassified(100);unclassified(100);
934	1	1	0	0	Bacteria(100);"Fusobacteria"(100);"Fusobacteria"(100);"Fusobacteriales"(100);"Fusobacteriaceae"(100);unclassified(100);
935	1	1	0	0	Bacteria(100);Firmicutes(100);Bacilli(100);Lactobacillales(100);unclassified(100);unclassified(100);
936	1	1	0	0	Bacteria(100);unclassified(100);unclassified(100);unclassified(100);unclassified(100);unclassified(100);
937	1	1	0	0	Bacteria(100);unclassified(100);unclassified(100);unclassified(100);unclassified(100);unclassified(100);
938	1	1	0	0	Bacteria(100);unclassified(100);unclassified(100);unclassified(100);unclassified(100);unclassified(100);
939	1	1	0	0	Bacteria(100);unclassified(100);unclassified(100);unclassified(100);unclassified(100);unclassified(100);
940	1	1	0	0	Bacteria(100);"Actinobacteria"(100);Actinobacteria(100);Actinomycetales(100);Actinomycetaceae(100);Actinomyces(100);
941	1	1	0	0	Bacteria(100);unclassified(100);unclassified(100);unclassified(100);unclassified(100);unclassified(100);
942	1	1	0	0	Bacteria(100);unclassified(100);unclassified(100);unclassified(100);unclassified(100);unclassified(100);
943	1	1	0	0	Bacteria(100);Firmicutes(100);Bacilli(100);Lactobacillales(100);Streptococcaceae(100);Streptococcus(100);
944	1	1	0	0	Bacteria(100);"Proteobacteria"(100);Alphaproteobacteria(100);unclassified(100);unclassified(100);unclassified(100);
945	1	1	0	0	Bacteria(100);"Actinobacteria"(100);Actinobacteria(100);Actinomycetales(100);unclassified(100);unclassified(100);
946	2	2	0	0	Bacteria(100);"Fusobacteria"(100);"Fusobacteria"(100);"Fusobacteriales"(100);"Leptotrichiaceae"(100);unclassified(100);
947	1	1	0	0	Bacteria(100);Firmicutes(100);Negativicutes(100);Selenomonadales(100);Veillonellaceae(100);Megamonas(100);
948	1	1	0	0	Bacteria(100);"Proteobacteria"(100);Alphaproteobacteria(100);Rhodobacterales(100);Rhodobacteraceae(100);unclassified(100);
949	2	2	0	0	Bacteria(100);"Actinobacteria"(100);Actinobacteria(100);Actinomycetales(100);Actinomycetaceae(100);Actinomyces(100);
950	2	2	0	0	Bacteria(100);Firmicutes(100);Bacilli(100);Lactobacillales(100);unclassified(100);unclassified(100);
951	1	1	0	0	Bacteria(100);"Actinobacteria"(100);Actinobacteria(100);Actinomycetales(100);Micrococcaceae(100);Rothia(100);
952	1	1	0	0	Bacteria(100);Firmicutes(100);Bacilli(100);Lactobacillales(100);unclassified(100);unclassified(100);
953	1	1	0	0	Bacteria(100);unclassified(100);unclassified(100);unclassified(100);unclassified(100);unclassified(100);
954	2	2	0	0	Bacteria(100);Firmicutes(100);Negativicutes(100);Selenomonadales(100);Veillonellaceae(100);unclassified(100);
955	1	1	0	0	Bacteria(100);unclassified(100);unclassified(100);unclassified(100);unclassified(100);unclassified(100);
956	1	1	0	0	Bacteria(100);"Proteobacteria"(100);Betaproteobacteria(100);Neisseriales(100);Neisseriaceae(100);unclassified(100);
957	1	1	0	0	Bacteria(100);"Fusobacteria"(100);"Fusobacteria"(100);"Fusobacteriales"(100);"Fusobacteriaceae"(100);Fusobacterium(100);

958	1	1	0	0	Bacteria(100);"Proteobacteria"(100);Gammaproteobacteria(100);Pseudomonadales(100);Pseudomonadaceae(100);unclassified(100);
959	1	1	0	0	Bacteria(100);Firmicutes(100);Clostridia(100);Clostridiales(100);Clostridiaceae_1(100);unclassified(100);
960	1	1	0	0	Bacteria(100);Firmicutes(100);Clostridia(100);Clostridiales(100);Clostridiaceae_1(100);unclassified(100);
961	1	1	0	0	Bacteria(100);"Proteobacteria"(100);Alphaproteobacteria(100);Caulobacterales(100);Caulobacteraceae(100);Brevundimonas(100);
962	1	1	0	0	Bacteria(100);"Proteobacteria"(100);Gammaproteobacteria(100);Pasteurellales(100);Pasteurellaceae(100);unclassified(100);
963	1	1	0	0	Bacteria(100);"Proteobacteria"(100);Betaproteobacteria(100);Neisseriales(100);Neisseriaceae(100);Neisseria(100);
964	1	1	0	0	Bacteria(100);"Fusobacteria"(100);"Fusobacteria"(100);"Fusobacteriales"(100);"Fusobacteriaceae"(100);unclassified(100);
965	1	1	0	0	Bacteria(100);unclassified(100);unclassified(100);unclassified(100);unclassified(100);unclassified(100);
966	1	1	0	0	Bacteria(100);"Proteobacteria"(100);Betaproteobacteria(100);Neisseriales(100);Neisseriaceae(100);unclassified(100);
967	1	1	0	0	Bacteria(100);unclassified(100);unclassified(100);unclassified(100);unclassified(100);unclassified(100);
968	1	1	0	0	Bacteria(100);"Proteobacteria"(100);Alphaproteobacteria(100);Rhodobacterales(100);Rhodobacteraceae(100);unclassified(100);
969	1	1	0	0	Bacteria(100);Firmicutes(100);Bacilli(100);Lactobacillales(100);unclassified(100);unclassified(100);
970	1	1	0	0	Bacteria(100);unclassified(100);unclassified(100);unclassified(100);unclassified(100);unclassified(100);
971	2	2	0	0	Bacteria(100);"Proteobacteria"(100);Alphaproteobacteria(100);Rhodospirillales(100);Acetobacteraceae(100);Roseomonas(100);
972	2	2	0	0	Bacteria(100);unclassified(100);unclassified(100);unclassified(100);unclassified(100);unclassified(100);
973	1	1	0	0	Bacteria(100);"Actinobacteria"(100);Actinobacteria(100);Actinomycetales(100);Micrococcaceae(100);Rothia(100);
974	1	1	0	0	Bacteria(100);"Proteobacteria"(100);Epsilonproteobacteria(100);Campylobacterales(100);Campylobacteraceae(100);Campylobacter(100);
975	1	1	0	0	Bacteria(100);"Proteobacteria"(100);Alphaproteobacteria(100);Rhizobiales(100);Bradyrhizobiaceae(100);unclassified(100);
976	2	2	0	0	Bacteria(100);unclassified(100);unclassified(100);unclassified(100);unclassified(100);unclassified(100);
977	2	2	0	0	Bacteria(100);Firmicutes(100);Clostridia(100);Clostridiales(100);Lachnospiraceae(100);Lachnospiracea_incertae_sedis(100);
978	2	2	0	0	Bacteria(100);Firmicutes(100);Bacilli(100);Lactobacillales(100);Aerococcaceae(100);unclassified(100);
979	1	1	0	0	Bacteria(100);Firmicutes(100);Bacilli(100);unclassified(100);unclassified(100);unclassified(100);
980	1	1	0	0	Bacteria(100);unclassified(100);unclassified(100);unclassified(100);unclassified(100);unclassified(100);
981	1	1	0	0	Bacteria(100);"Proteobacteria"(100);Betaproteobacteria(100);Neisseriales(100);Neisseriaceae(100);unclassified(100);
982	1	1	0	0	Bacteria(100);Firmicutes(100);Bacilli(100);Lactobacillales(100);Streptococcaceae(100);Streptococcus(100);
983	1	1	0	0	Bacteria(100);Firmicutes(100);Bacilli(100);Lactobacillales(100);unclassified(100);unclassified(100);
984	1	1	0	0	Bacteria(100);"Actinobacteria"(100);Actinobacteria(100);Acidimicrobiales(100);unclassified(100);unclassified(100);
985	1	1	0	0	Bacteria(100);"Actinobacteria"(100);Actinobacteria(100);Actinomycetales(100);Corynebacteriaceae(100);Corynebacterium(100);

986	1	1	0	0	Bacteria(100);"Proteobacteria"(100);Alphaproteobacteria(100);unclassified(100);unclassified(100);unclassified(100);
987	1	1	0	0	Bacteria(100);unclassified(100);unclassified(100);unclassified(100);unclassified(100);unclassified(100);
988	1	1	0	0	Bacteria(100);Firmicutes(100);Clostridia(100);Clostridiales(100);Lachnospiraceae(100);Syntrophococcus(100);
989	1	1	0	0	Bacteria(100);unclassified(100);unclassified(100);unclassified(100);unclassified(100);unclassified(100);
990	1	1	0	0	Bacteria(100);unclassified(100);unclassified(100);unclassified(100);unclassified(100);unclassified(100);
991	1	1	0	0	Bacteria(100);"Actinobacteria"(100);Actinobacteria(100);Actinomycetales(100);unclassified(100);unclassified(100);
992	1	1	0	0	Bacteria(100);unclassified(100);unclassified(100);unclassified(100);unclassified(100);unclassified(100);
993	1	1	0	0	Bacteria(100);"Proteobacteria"(100);Gammaproteobacteria(100);Pseudomonadales(100);Moraxellaceae(100);Acinetobacter(100);
994	1	1	0	0	Bacteria(100);"Proteobacteria"(100);Gammaproteobacteria(100);Gammaproteobacteria_order_incertae_sedis(100);Gammaproteobacteria_family_incertae_sedis(100);unclassified(100);
995	1	1	0	0	Bacteria(100);"Proteobacteria"(100);Alphaproteobacteria(100);Rhizobiales(100);unclassified(100);unclassified(100);
996	1	1	0	0	Bacteria(100);"Deinococcus-Thermus"(100);Deinococci(100);Deinococcales(100);Deinococcaceae(100);Deinococcus(100);
997	1	1	0	0	Bacteria(100);"Proteobacteria"(100);Gammaproteobacteria(100);Pasteurellales(100);Pasteurellaceae(100);unclassified(100);
998	1	0	1	0	Bacteria(100);"Actinobacteria"(100);Actinobacteria(100);Actinomycetales(100);Micrococcaceae(100);Rothia(100);
999	1	1	0	0	Bacteria(100);unclassified(100);unclassified(100);unclassified(100);unclassified(100);unclassified(100);
###	1	1	0	0	Bacteria(100);"Bacteroidetes"(100);"Bacteroidia"(100);"Bacteroidales"(100);"Porphyromonadaceae"(100);unclassified(100);
###	1	1	0	0	Bacteria(100);"Actinobacteria"(100);Actinobacteria(100);unclassified(100);unclassified(100);unclassified(100);
###	1	1	0	0	Bacteria(100);"Proteobacteria"(100);Alphaproteobacteria(100);unclassified(100);unclassified(100);unclassified(100);
###	2	2	0	0	Bacteria(100);unclassified(100);unclassified(100);unclassified(100);unclassified(100);unclassified(100);
###	2	2	0	0	Bacteria(100);Firmicutes(100);Clostridia(100);Clostridiales(100);unclassified(100);unclassified(100);
###	1	1	0	0	Bacteria(100);"Actinobacteria"(100);Actinobacteria(100);Actinomycetales(100);unclassified(100);unclassified(100);
###	1	1	0	0	Bacteria(100);"Proteobacteria"(100);Deltaproteobacteria(100);Desulfovibrionales(100);Desulfovibrionaceae(100);Desulfovibrio(100);
###	1	1	0	0	Bacteria(100);unclassified(100);unclassified(100);unclassified(100);unclassified(100);unclassified(100);
###	1	1	0	0	Bacteria(100);Firmicutes(100);Clostridia(100);Clostridiales(100);Peptostreptococcaceae(100);Clostridium_XI(100);
###	1	1	0	0	Bacteria(100);"Proteobacteria"(100);Gammaproteobacteria(100);Gammaproteobacteria_order_incertae_sedis(100);Gammaproteobacteria_family_incertae_sedis(100);unclassified(100);
###	3	3	0	0	Bacteria(100);unclassified(100);unclassified(100);unclassified(100);unclassified(100);unclassified(100);
###	1	1	0	0	Bacteria(100);"Proteobacteria"(100);Gammaproteobacteria(100);unclassified(100);unclassified(100);unclassified(100);
###	1	1	0	0	Bacteria(100);Firmicutes(100);Clostridia(100);Clostridiales(100);Clostridiales_Incertae_Sedis_XI(100);Peptoniphilus(100);
###	1	1	0	0	Bacteria(100);Firmicutes(100);Bacilli(100);Lactobacillales(100);Streptococcaceae(100);Streptococcus(100);
###	1	1	0	0	Bacteria(100);Firmicutes(100);Erysipelotrichia(100);Erysipelotrichales(100);Erysipelotrichaceae(100);Bulleidia(100);

###	1	1	0	0	Bacteria(100);"Proteobacteria"(100);Gammaproteobacteria(100);Legionellales(100);Legionellaceae(100);Legionella(100);
###	1	1	0	0	Bacteria(100);"Proteobacteria"(100);Gammaproteobacteria(100);Pseudomonadales(100);Pseudomonadaceae(100);unclassified(100);
###	1	1	0	0	Bacteria(100);"Actinobacteria"(100);Actinobacteria(100);Bifidobacteriales(100);Bifidobacteriaceae(100);Gardnerella(100);
###	1	1	0	0	Bacteria(100);Firmicutes(100);Bacilli(100);Lactobacillales(100);Streptococcaceae(100);Streptococcus(100);
###	1	1	0	0	Bacteria(100);"Bacteroidetes"(100);"Sphingobacteria"(100);"Sphingobacteriales"(100);Chitinophagaceae(100);unclassified(100);
###	1	1	0	0	Bacteria(100);unclassified(100);unclassified(100);unclassified(100);unclassified(100);unclassified(100);
###	1	1	0	0	Bacteria(100);"Actinobacteria"(100);Actinobacteria(100);Rubrobacterales(100);Rubrobacteraceae(100);Rubrobacter(100);
###	1	1	0	0	Bacteria(100);"Proteobacteria"(100);Alphaproteobacteria(100);Sphingomonadales(100);Sphingomonadaceae(100);unclassified(100);
###	1	1	0	0	Bacteria(100);unclassified(100);unclassified(100);unclassified(100);unclassified(100);unclassified(100);
###	1	1	0	0	Bacteria(100);"Proteobacteria"(100);Betaproteobacteria(100);Neisseriales(100);Neisseriaceae(100);unclassified(100);
###	2	2	0	0	Bacteria(100);"Proteobacteria"(100);Betaproteobacteria(100);Rhodocyclales(100);Rhodocyclaceae(100);Zoogloea(100);
###	1	1	0	0	Bacteria(100);"Proteobacteria"(100);Epsilonproteobacteria(100);Campylobacterales(100);Helicobacteraceae(100);unclassified(100);
###	1	1	0	0	Bacteria(100);"Proteobacteria"(100);Alphaproteobacteria(100);Sphingomonadales(100);Sphingomonadaceae(100);unclassified(100);
###	1	1	0	0	Bacteria(100);unclassified(100);unclassified(100);unclassified(100);unclassified(100);unclassified(100);
###	2	2	0	0	Bacteria(100);Firmicutes(100);Bacilli(100);Lactobacillales(100);Streptococcaceae(100);Streptococcus(100);
###	3	3	0	0	Bacteria(100);Firmicutes(100);Clostridia(100);Clostridiales(100);unclassified(100);unclassified(100);
###	2	2	0	0	Bacteria(100);"Proteobacteria"(100);Gammaproteobacteria(100);"Enterobacteriales"(100);Enterobacteriaceae(100);Escherichia_Shigella(100);
###	1	1	0	0	Bacteria(100);"Proteobacteria"(100);Betaproteobacteria(100);Neisseriales(100);Neisseriaceae(100);unclassified(100);
###	2	2	0	0	Bacteria(100);"Bacteroidetes"(100);"Bacteroidia"(100);"Bacteroidales"(100);"Prevotellaceae"(100);Prevotella(100);
###	1	1	0	0	Bacteria(100);"Proteobacteria"(100);Alphaproteobacteria(100);Rhodospirillales(100);Acetobacteraceae(100);Rhodovarius(100);
###	1	1	0	0	Bacteria(100);"Proteobacteria"(100);Betaproteobacteria(100);Neisseriales(100);Neisseriaceae(100);unclassified(100);
###	1	1	0	0	Bacteria(100);"Fusobacteria"(100);"Fusobacteria"(100);"Fusobacteriales"(100);"Fusobacteriaceae"(100);Fusobacterium(100);
###	1	1	0	0	Bacteria(100);Firmicutes(100);Bacilli(100);unclassified(100);unclassified(100);unclassified(100);
###	2	2	0	0	Bacteria(100);"Bacteroidetes"(100);Flavobacteria(100);"Flavobacteriales"(100);Flavobacteriaceae(100);Chryseobacterium(100);
###	1	1	0	0	Bacteria(100);"Proteobacteria"(100);Gammaproteobacteria(100);Pseudomonadales(100);Moraxellaceae(100);Moraxella(100);
###	1	1	0	0	Bacteria(100);unclassified(100);unclassified(100);unclassified(100);unclassified(100);unclassified(100);

###	1	1	0	0	Bacteria(100);unclassified(100);unclassified(100);unclassified(100);unclassified(100);unclassified(100);
###	1	1	0	0	Bacteria(100);"Proteobacteria"(100);Deltaproteobacteria(100);Bdellovibrionales(100);Bacteriovoracaceae(100);Peredibacter(100);
###	1	1	0	0	Bacteria(100);Firmicutes(100);Clostridia(100);Clostridiales(100);unclassified(100);unclassified(100);
###	1	1	0	0	Bacteria(100);"Proteobacteria"(100);Alphaproteobacteria(100);unclassified(100);unclassified(100);unclassified(100);
###	1	1	0	0	Bacteria(100);Firmicutes(100);Clostridia(100);Clostridiales(100);Peptostreptococcaceae(100);unclassified(100);
###	1	1	0	0	Bacteria(100);unclassified(100);unclassified(100);unclassified(100);unclassified(100);unclassified(100);
###	2	2	0	0	Bacteria(100);"Proteobacteria"(100);Alphaproteobacteria(100);Rhodospirillales(100);Acetobacteraceae(100);unclassified(100);
###	1	1	0	0	Bacteria(100);"Proteobacteria"(100);Alphaproteobacteria(100);Rhizobiales(100);unclassified(100);unclassified(100);
###	1	1	0	0	Bacteria(100);Firmicutes(100);Clostridia(100);Clostridiales(100);unclassified(100);unclassified(100);
###	1	1	0	0	Bacteria(100);"Bacteroidetes"(100);Flavobacteria(100);"Flavobacteriales"(100);Flavobacteriaceae(100);Flavobacterium(100);
###	1	1	0	0	Bacteria(100);Firmicutes(100);Bacilli(100);Lactobacillales(100);Streptococcaceae(100);Streptococcus(100);
###	1	1	0	0	Bacteria(100);Firmicutes(100);unclassified(100);unclassified(100);unclassified(100);unclassified(100);
###	1	1	0	0	Bacteria(100);"Bacteroidetes"(100);"Bacteroidia"(100);"Bacteroidales"(100);"Rikenellaceae"(100);Alistipes(100);
###	1	1	0	0	Bacteria(100);unclassified(100);unclassified(100);unclassified(100);unclassified(100);unclassified(100);
###	1	1	0	0	Bacteria(100);"Proteobacteria"(100);Gammaproteobacteria(100);Pasteurellales(100);Pasteurellaceae(100);unclassified(100);
###	1	1	0	0	Bacteria(100);"Proteobacteria"(100);Gammaproteobacteria(100);unclassified(100);unclassified(100);unclassified(100);
###	1	1	0	0	Bacteria(100);"Proteobacteria"(100);Alphaproteobacteria(100);Rhizobiales(100);unclassified(100);unclassified(100);
###	2	2	0	0	Bacteria(100);Firmicutes(100);Bacilli(100);Bacillales(100);Paenibacillaceae_1(100);Paenibacillus(100);
###	1	1	0	0	Bacteria(100);Firmicutes(100);unclassified(100);unclassified(100);unclassified(100);unclassified(100);
###	1	1	0	0	Bacteria(100);"Bacteroidetes"(100);unclassified(100);unclassified(100);unclassified(100);unclassified(100);
###	1	1	0	0	Bacteria(100);"Proteobacteria"(100);Epsilonproteobacteria(100);Campylobacterales(100);Campylobacteraceae(100);Campylobacter(100);
###	1	1	0	0	Bacteria(100);"Proteobacteria"(100);Alphaproteobacteria(100);unclassified(100);unclassified(100);unclassified(100);
###	1	1	0	0	Bacteria(100);unclassified(100);unclassified(100);unclassified(100);unclassified(100);unclassified(100);
###	2	2	0	0	Bacteria(100);"Bacteroidetes"(100);"Bacteroidia"(100);"Bacteroidales"(100);"Prevotellaceae"(100);Prevotella(100);
###	2	2	0	0	Bacteria(100);"Bacteroidetes"(100);Flavobacteria(100);"Flavobacteriales"(100);Flavobacteriaceae(100);Flavobacterium(100);
###	1	1	0	0	Bacteria(100);"Proteobacteria"(100);Betaproteobacteria(100);Burkholderiales(100);Comamonadaceae(100);unclassified(100);
###	2	2	0	0	Bacteria(100);Firmicutes(100);Bacilli(100);Lactobacillales(100);Streptococcaceae(100);Streptococcus(100);
###	2	2	0	0	Bacteria(100);"Proteobacteria"(100);Epsilonproteobacteria(100);Campylobacterales(100);Helicobacteraceae(100);Helicobacter(100);
###	1	1	0	0	Bacteria(100);"Chloroflexi"(100);unclassified(100);unclassified(100);unclassified(100);unclassified(100);

###	1	1	0	0	Bacteria(100);"Proteobacteria"(100);Betaproteobacteria(100);Burkholderiales(100);unclassified(100);unclassified(100);
###	1	1	0	0	Bacteria(100);"Proteobacteria"(100);Alphaproteobacteria(100);Rhodospirillales(100);Acetobacteraceae(100);unclassified(100);
###	1	1	0	0	Bacteria(100);unclassified(100);unclassified(100);unclassified(100);unclassified(100);unclassified(100);
###	2	2	0	0	Bacteria(100);unclassified(100);unclassified(100);unclassified(100);unclassified(100);unclassified(100);
###	1	1	0	0	Bacteria(100);"Actinobacteria"(100);Actinobacteria(100);Bifidobacteriales(100);Bifidobacteriaceae(100);Gardnerella(100);
###	3	3	0	0	Bacteria(100);"Proteobacteria"(100);Gammaproteobacteria(100);"Enterobacteriales"(100);Enterobacteriaceae(100);unclassified(100<br>);
###	1	1	0	0	Bacteria(100);unclassified(100);unclassified(100);unclassified(100);unclassified(100);unclassified(100);
###	1	1	0	0	Bacteria(100);"Fusobacteria"(100);"Fusobacteria"(100);"Fusobacteriales"(100);"Fusobacteriaceae"(100);unclassified(100);
###	1	1	0	0	Bacteria(100);unclassified(100);unclassified(100);unclassified(100);unclassified(100);unclassified(100);
###	2	2	0	0	Bacteria(100);"Proteobacteria"(100);Deltaproteobacteria(100);Bdellovibrionales(100);Bacteriovoracaceae(100);Peredibacter(100);
###	1	1	0	0	Bacteria(100);unclassified(100);unclassified(100);unclassified(100);unclassified(100);unclassified(100);
###	1	1	0	0	Bacteria(100);"Bacteroidetes"(100);"Sphingobacteria"(100);"Sphingobacteriales"(100);Sphingobacteriaceae(100);unclassified(100);
###	1	1	0	0	Bacteria(100);Firmicutes(100);Bacilli(100);Lactobacillales(100);Streptococcaceae(100);Streptococcus(100);
###	1	1	0	0	Bacteria(100);Firmicutes(100);Bacilli(100);Bacillales(100);Staphylococcaceae(100);Staphylococcus(100);
###	1	1	0	0	Bacteria(100);"Bacteroidetes"(100);Flavobacteria(100);"Flavobacteriales"(100);Flavobacteriaceae(100);unclassified(100);
###	2	2	0	0	Bacteria(100);Firmicutes(100);Clostridia(100);Clostridiales(100);Clostridiales_Incertae_Sedis_XI(100);Parvimonas(100);
###	1	1	0	0	Bacteria(100);"Proteobacteria"(100);Gammaproteobacteria(100);unclassified(100);unclassified(100);unclassified(100);
###	1	1	0	0	Bacteria(100);"Proteobacteria"(100);Betaproteobacteria(100);Neisseriales(100);Neisseriaceae(100);Bergeriella(100);
###	1	1	0	0	Bacteria(100);"Fusobacteria"(100);"Fusobacteria"(100);"Fusobacteriales"(100);"Fusobacteriaceae"(100);Fusobacterium(100);
###	1	1	0	0	Bacteria(100);"Bacteroidetes"(100);"Bacteroidia"(100);"Bacteroidales"(100);"Prevotellaceae"(100);Prevotella(100);
###	1	1	0	0	Bacteria(100);"Actinobacteria"(100);Actinobacteria(100);Actinomycetales(100);unclassified(100);unclassified(100);
###	1	1	0	0	Bacteria(100);"Proteobacteria"(100);Gammaproteobacteria(100);unclassified(100);unclassified(100);unclassified(100);
###	1	1	0	0	Bacteria(100);"Proteobacteria"(100);Betaproteobacteria(100);Neisseriales(100);Neisseriaceae(100);unclassified(100);
###	1	1	0	0	Bacteria(100);"Proteobacteria"(100);Alphaproteobacteria(100);unclassified(100);unclassified(100);unclassified(100);
###	6	2	0	4	Bacteria(100);Firmicutes(100);Bacilli(100);Bacillales(100);Listeriaceae(100);Brochothrix(100);
###	2	2	0	0	Bacteria(100);unclassified(100);unclassified(100);unclassified(100);unclassified(100);unclassified(100);
###	1	1	0	0	Bacteria(100);Firmicutes(100);Clostridia(100);Clostridiales(100);Lachnospiraceae(100);unclassified(100);
###	4	4	0	0	Bacteria(100);"Actinobacteria"(100);Actinobacteria(100);Actinomycetales(100);Kineosporiaceae(100);unclassified(100);
###	1	1	0	0	Bacteria(100);unclassified(100);unclassified(100);unclassified(100);unclassified(100);unclassified(100);

###	1	1	0	0	Bacteria(100);Firmicutes(100);Clostridia(100);Clostridiales(100);Clostridiales_Incertae_Sedis_XI(100);Anaerococcus(100);
###	1	1	0	0	Bacteria(100);Firmicutes(100);Clostridia(100);Clostridiales(100);Clostridiales_Incertae_Sedis_XI(100);Anaerococcus(100);
###	2	2	0	0	Bacteria(100);"Bacteroidetes"(100);unclassified(100);unclassified(100);unclassified(100);unclassified(100);

Table S2: Genera taxonomic classifications and their abundance within each plant type

Legend: irPMT represent transformed *Nicotiana attenuata* samples, WT represent *Nicotiana attenuata* normal samples, ISWT represent *N. glauca* samples.

	irPMT	WT	ISWT
Abiotrophia	0.03%		
Acidithiobacillus	0.31%	1.11%	0.58%
Acidovorax		0.01%	
Actinomyces	0.02%		
Aeromonas	0.14%	0.03%	0.05%
Aggregatibacter	0.15%	0.06%	0.04%
Albidiferax	0.01%	0.01%	0.01%
Alistipes	0.02%		
Alkanindiges	0.13%	0.20%	1.05%
Anaerobacter	0.02%		0.03%
Anaerococcus	1.87%	1.38%	2.94%
Anaerostipes	0.04%		
Arcobacter	0.42%	0.14%	
Arsenicicoccus	0.04%	0.02%	0.02%
Arthrobacter	0.01%		0.01%
Atopobium	0.02%		
Bacteroides	0.17%	0.12%	0.05%
Balneimonas	0.07%	0.01%	0.07%
Bergeriella	0.21%	0.15%	
Bifidobacterium	1.52%	0.29%	
Blastococcus	0.05%		
Blastomonas	0.02%		
Blautia	0.01%		
Bosea	0.01%		
Brachybacterium	0.01%		
Brevibacterium	7.02%	7.73%	5.19%
Brevundimonas	0.01%		
Brochothrix	0.01%		
Bulleidia	0.01%		
Burkholderia	0.02%		
Campylobacter	0.01%		
Capnocytophaga	0.38%	0.00%	0.05%
Cardiobacterium	0.01%		
Carnobacterium	0.01%		
Catellicoccus	0.79%	0.87%	0.38%
Catenibacterium	0.02%		
Caulobacter	0.01%		
Cellvibrio	1.15%	0.38%	1.18%
Centipeda	0.16%	0.15%	0.03%
Cetobacterium	3.24%	2.87%	0.94%
Chitinimonas	0.03%		
Chryseobacterium	0.02%		0.07%
Cloacibacterium	0.04%		
Clostridium_sensu_stricto	0.05%		
Clostridium_XI	0.02%	0.02%	0.01%

Clostridium_XlVb	0.01%		
Collinsella	0.01%		
Comamonas	0.01%		
Corynebacterium	0.01%		
Cupriavidus	0.02%		
Curvibacter	0.02%		
Deinococcus			
Delftia	0.31%	0.26%	0.46%
Desulfovibrio	0.01%		
Devosia	0.01%		
Dietzia	0.02%		0.02%
Enhydrobacter	0.06%	0.01%	
Enterococcus	0.02%		
Epilithonimonas	0.06%		0.02%
Eubacterium	0.10%	0.05%	
Exiguobacterium	0.00%		0.01%
Facklamia	0.01%		
Filifactor	0.09%	0.02%	
Finegoldia	0.05%	0.04%	
Fluviicola	0.00%		
Frateuria	0.02%	0.01%	
Friedmanniella	0.37%	0.34%	0.23%
Fusobacterium	0.07%		
Gardnerella	0.01%		
Gemella	0.02%		
Gemmatimonas	0.24%	0.02%	0.06%
Gordonia	0.04%		
Gp4	0.01%		
Gp6	0.01%		
Granulicatella	0.02%		
Haematobacter	0.16%	0.75%	0.15%
Haemophilus	0.01%		
Halomonas	0.05%		
Herbaspirillum	0.04%		0.02%
Howardella	0.03%		
Hydrotalea	0.02%		
Idiomarina	0.44%	0.18%	0.02%
Ilumatobacter	0.01%		
Janibacter	0.02%		
Johnsonella	0.01%		
Kingella	8.02%	4.61%	1.12%
Kocuria	1.29%	1.06%	1.25%
Kytococcus	0.23%	0.17%	0.04%
Lachnospiracea_incertae_sedis	0.03%		
Lactobacillus	0.03%		
Lactococcus	20.68%	24.51%	8.21%
Legionella	0.54%	0.39%	0.64%
Leptotrichia	0.03%		
Leuconostoc	0.07%	0.28%	
Lysobacter	0.03%		

Mannheimia	1.85%	1.42%	1.34%
Marinobacter	0.01%		
Marmoricola	0.05%		0.02%
Massilia	0.04%	0.01%	0.08%
Megamonas	0.05%	0.01%	0.01%
Meiothermus	0.01%		
Methylobacterium	0.01%		
Micrococcus			0.01%
Mobiluncus	0.01%		
Modestobacter	0.01%		
Mogibacterium	0.07%	0.02%	
Moraxella	0.02%		
Mycobacterium	0.03%		
Mycoplasma	0.02%		
Naxibacter	0.07%		0.01%
Negativicoccus	0.32%	0.51%	0.03%
Neisseria	0.04%		
Nesterenkonia	4.18%	7.69%	1.35%
Nocardioides	0.03%		
Nubsella	0.06%		
Ochrobactrum	0.03%		
Olsenella	0.01%		
OP11_genus_incertae_sedis	0.09%	0.02%	0.01%
Paenibacillus	0.00%		
Paracoccus	0.15%	0.06%	0.01%
Paraprevotella	1.36%	0.61%	0.08%
Parvimonas	1.06%	0.64%	0.62%
Pedobacter	0.04%		
Pelomonas	0.02%		
Peptococcus	0.02%		
Peptoniphilus	0.03%	0.01%	
Peptostreptococcaceae_incertae_sedis	0.28%	0.19%	0.21%
Peptostreptococcus	0.01%		
Phycicoccus	0.05%		
Porphyromonas	0.08%	0.03%	
Prevotella	0.03%	0.00%	0.01%
Propioniferax	0.01%		
Prosthecobacter	0.00%		
Pseudolabrys	0.06%		
Pseudomonas	0.27%	0.03%	0.06%
Psychrobacter	0.02%		
Ralstonia	0.01%		
Rheinheimera	0.01%		
Rhizobium	0.01%		
Rhodanobacter	0.01%		
Rhodoferax	0.08%	0.02%	0.07%
Roseomonas	0.01%		
Rothia	0.18%	0.04%	0.07%
Rubrobacter	0.02%		
Ruminococcus	0.06%		

Sediminibacterium	0.01%		
Segetibacter	0.07%		
Selenomonas	0.98%	0.48%	0.57%
Serratia	0.03%	0.02%	0.01%
Sphingobacterium	0.01%		
Sphingobium	0.20%	0.01%	0.04%
Sphingomonas	0.02%		
Sphingopyxis	0.02%		
SR1_genus_incertae_sedis	0.29%	0.03%	
Staphylococcus	0.07%		
Stenotrophomonas	0.01%		
Streptophyta	0.02%		
Sulfurospirillum	0.02%		
Sutterella	0.12%	0.01%	0.13%
Syntrophococcus	0.48%	0.10%	0.03%
Tannerella	0.15%	0.03%	0.02%
Thiofaba	0.00%		
TM7_genus_incertae_sedis	1.45%	0.63%	0.13%
Treponema	0.03%		
Trichococcus	0.39%	0.43%	0.13%
Turicella	0.09%		
unclassified	7.70%	8.24%	3.27%
unclassified	4.48%	4.39%	1.35%
unclassified	1.86%	1.87%	0.33%
unclassified	1.75%	0.65%	1.45%
unclassified	1.35%	1.00%	1.07%
unclassified	1.29%	0.90%	0.45%
unclassified	1.29%	1.52%	0.77%
unclassified	1.17%	0.57%	0.07%
unclassified	1.12%	0.34%	0.49%
unclassified	0.90%	0.85%	0.45%
unclassified	0.87%	0.38%	0.30%
unclassified	0.53%	0.21%	
unclassified	0.50%	0.29%	0.54%
unclassified	0.39%	0.19%	0.34%
unclassified	0.35%	0.20%	0.12%
unclassified	0.20%	1.65%	
unclassified	0.18%	0.09%	0.06%
unclassified	0.15%	0.04%	0.03%
unclassified	0.10%	0.02%	0.06%
unclassified	0.10%		
unclassified	0.08%	0.01%	
unclassified	0.07%		
unclassified	0.07%		
unclassified	0.06%		
unclassified	0.06%		0.15%
unclassified	0.06%	0.04%	
unclassified	0.05%		
unclassified	0.05%		
unclassified	0.04%	0.19%	1.09%

unclassified	0.04%		0.01%
unclassified	0.04%		
unclassified	0.04%		
unclassified	0.04%		
unclassified	0.03%		
unclassified	0.03%		
unclassified	0.03%		
unclassified	0.03%	0.01%	
unclassified	0.03%		
unclassified	0.03%		
unclassified	0.03%	0.01%	0.01%
unclassified	0.03%		
unclassified	0.02%	0.01%	
unclassified	0.02%		
unclassified	0.02%		
unclassified	0.02%	0.01%	
unclassified	0.02%		
unclassified	0.02%		
unclassified	0.02%	0.01%	0.02%
unclassified	0.02%	0.00%	0.01%
unclassified	0.01%		
unclassified	0.01%		
unclassified	0.01%		
unclassified	0.01%		
unclassified	0.01%		
unclassified	0.01%		
unclassified	0.01%		
unclassified	0.01%		
unclassified	0.01%		
unclassified	0.01%		
unclassified	0.01%		
unclassified	0.01%		
unclassified	0.01%		
unclassified	0.01%		
unclassified	0.01%		
unclassified	0.01%		
unclassified	0.01%		
unclassified	0.01%		
Ureaplasma	0.00%		
Varibaculum	0.01%		
Veillonella	0.23%	0.02%	0.01%
Verrucomicrobium	0.02%		
Vibrio	0.06%		
Vogesella	0.94%	0.41%	0.15%
Wautersiella	0.02%		
Weissella	0.45%	0.22%	0.16%
Xanthomonas	0.05%		
Yersinia	3.28%	14.36%	57.25%
Zimmermannella	0.05%		
